# SurGen: 1020 H&E-stained whole-slide images with survival and genetic markers

**DOI:** 10.1093/gigascience/giaf086

**Published:** 2025-10-08

**Authors:** Craig Myles, In Hwa Um, Craig Marshall, David Harris-Birtill, David J Harrison

**Affiliations:** School of Computer Science, University of St Andrews, St Andrews KY16 9SX, UK; School of Medicine, University of St Andrews, St Andrews KY16 9TF, UK; Lothian Biorepository, NHS Lothian, Edinburgh EH16 4SA, UK; School of Computer Science, University of St Andrews, St Andrews KY16 9SX, UK; School of Medicine, University of St Andrews, St Andrews KY16 9TF, UK; NHS Lothian Pathology, Division of Laboratory Medicine, Royal Infirmary of Edinburgh, Edinburgh EH16 4SA, UK

**Keywords:** whole-slide image (WSI), hematoxylin and eosin (H&E) stain, mismatch repair (MMR), microsatellite instability (MSI), KRAS mutation, NRAS mutation, BRAF mutation, colorectal cancer, digital pathology, dataset

## Abstract

**Background:**

Cancer remains one of the leading causes of morbidity and mortality worldwide. Comprehensive datasets that combine histopathological images with genetic and survival data across various tumour sites are essential for advancing computational pathology and personalised medicine.

**Results:**

We present SurGen, a dataset comprising 1,020 H&E-stained whole-slide images (WSIs) from 843 colorectal cancer cases. The dataset includes detailed annotations for key genetic mutations (*KRAS, NRAS, BRAF*) and mismatch repair status, as well as survival data for 426 cases. We illustrate SurGen’s utility with a proof-of-concept model that predicts mismatch repair status directly from WSIs, achieving a test area under the receiver operating characteristic curve of 0.8273. These preliminary results underscore the dataset’s potential to facilitate research in biomarker discovery, prognostic modelling, and advanced machine learning applications in colorectal cancer and beyond.

**Conclusions:**

SurGen offers a valuable resource for the scientific community, enabling studies that require high-quality WSIs linked with comprehensive clinical and genetic information on colorectal cancer. Our initial findings affirm the dataset’s capacity to advance diagnostic precision and foster the development of personalised treatment strategies in colorectal oncology. Data available online: https://doi.org/10.6019/S-BIAD1285.

## Background

Colorectal cancer is among the most common and lethal cancers worldwide, with over 900,000 deaths occurring each year [1, 2]. Advances in computational pathology and machine learning have the potential to revolutionize cancer diagnosis and treatment by enabling the analysis of complex histopathological and genetic data across various tumour types [3, 4].

High-quality datasets that combine whole-slide images (WSIs) with detailed clinical and genetic annotations are crucial for developing and validating computational models. However, the field currently faces significant limitations due to the scarcity of publicly available annotated datasets that integrate both imaging and nonimaging patient data [5]. Existing datasets often focus on specific cancer sites, such as breast [6–8], gastric and colorectal [9, 10], and lung [11], or lack comprehensive annotations necessary for advanced computational pathology research. Additionally, the quality of publicly available samples can be highly variable, potentially hindering the development of robust and generalisable models [5]. The SurGen dataset addresses these gaps by providing a diverse and high-quality collection of WSIs linked with genetic mutations, mismatch repair status, and cancer staging across colorectal and neighboring sites (See Table 1). Additionally, it includes survival data specifically for the primary colorectal cancer cohort, enhancing its value for prognostic studies in this prevalent cancer.

This article reports on the composition, collection, and potential applications of the SurGen dataset, highlighting its utility for both focused studies specific to primary colorectal cancer and broader investigations into metastatic tumour sites. This is particularly pertinent given that up to 50% of patients with localized disease eventually develop metastases [12].

The SurGen dataset is a comprehensive digital pathology resource designed to support a wide range of cancer and computational pathology research initiatives. It consists of WSIs coupled with detailed clinical and genetic data, spanning colorectal regions as well as neighboring metastatic sites. Table 2 provides a breakdown of tumour sites across the SurGen dataset. The SurGen dataset is divided into 2 distinct subsets:

1. **SR386** (**Colorectal Cohort with Survival Data**) focuses on primary colorectal cancer, consisting of 427 WSIs from 427 cases with a focus on colorectal tumour sites. This subset includes survival data in addition to biomarker labels, such as mutation status in the *KRAS, NRAS*, and *BRAF* genes, as well as mismatch repair (MMR) status. This makes it particularly valuable for research aimed at understanding the genetic and biomarker properties of colorectal cancer for the exploration and prediction of its clinical outcomes.
2. **SR1482** (**Colorectal Cancer with Metastatic Sites**) is a subset that contains 593 WSIs from 416 colorectal cancer cases. This cohort includes WSIs from both primary colorectal tumours and metastatic lesions in sites such as the liver, lung, peritoneum, and others. While it does not include survival data, it offers extensive biomarker information, making it valuable for studies on genetic and molecular characteristics of colorectal cancer and its metastatic behaviour.

**Table 1:** Comparative overview of publicly available formalin-fixed, paraffin-embedded (FFPE) H&E-stained colorectal whole-slide image datasets with relevant biomarker labels.

Dataset	Access	Origin	Cases	WSIs	Magnification	MPP	KRAS	NRAS	BRAF	MSI/MMR	Survival	Staging	Pathological	Segmentation
SurGen (Ours)	Public	GBR	843	1020	40×	0.1112	✓	✓	✓	✓	✓	✓	✓	✗
PAIP [10]	Upon Req.	KOR	118	118	40×	0.2522	✗	✗	✗	✓	✗	✗	✗	✓
TCGA-COAD [13]	Public	USA	451	459	20× or 40×	*0.2436	✓	✓	✓	✓	✓	✓	✓	✗
TCGA-READ [13]	Public	USA	164	165	20× or 40×	*0.2427	✓	✓	✓	✓	✓	✓	✓	✗
CPTAC-COAD [14]	Public	USA	105	220	40×	0.2501	✓	✓	✓	✓	✗	✓	✓	✗
CRC-Orion [15]	Public	USA	40	42	20×	0.3250	✓	✓	✓	✓	✓	✓	✓	✓

Note: Cases are only counted if at least 1 diagnostic WSI is available per clinical record. Note that reported case counts may differ across publications due to varying inclusion criteria and filtering methods. This table does not include any tumour microarray or patch-based datasets. MPP: Microns per pixel. MPP values marked with * are mean values across the cohort, with ranges: TCGA-COAD (0.2325–0.2527), TCGA-READ (0.2325–0.2520).

**Table 2:**
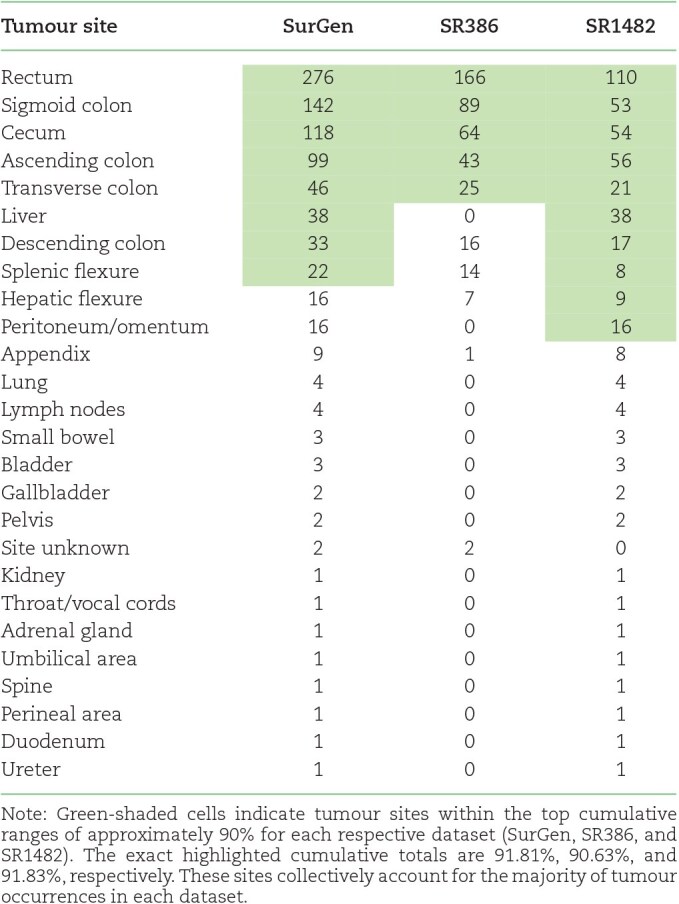
Tumour site counts for SurGen, SR386, and SR1482.

The SurGen dataset aims to facilitate research in oncology and digital pathology by providing high-quality, labelled WSIs that can be used for training and validating computational models, investigating tumour and oncological properties, and exploring biomarker-driven stratification in colorectal cancer. This article reports on the composition, collection, and potential applications of the SurGen dataset, highlighting its utility for both focused studies on colorectal cancer and broader investigations into neighboring metastatic sites and generalised oncological understanding.

To highlight the comprehensive nature of the SurGen dataset, we compare it with several publicly available colorectal cancer datasets. Table 1 summarises key attributes such as the inclusion of genetic markers, survival data, and tumour staging.

Other digital pathology collections also exist. For instance, the PLCO Cancer Screening Trial [16] offers controlled access to colorectal WSIs (approximately 2,800 images from 749 cases) but does not provide tumour-level molecular annotation, while the open-access HunCRC [17] biopsy dataset contains 200 annotated slides yet lacks molecular and survival data.

As shown in Table 1, the SurGen dataset provides a valuable addition to publicly available resources, uniquely integrating high-resolution WSIs with detailed genetic, clinical, and survival data. While datasets such as TCGA-COAD, TCGA-READ, and CPTAC-CRC offer comprehensive genomic sequencing data, SurGen complements these resources by focusing on key colorectal cancer biomarkers (*KRAS, NRAS, BRAF*, MSI/MMR) and survival outcomes. Moreover, its consistent high-resolution scanning at 40$\times$ magnification across all slides ensures uniform image quality, addressing variability seen in some datasets, such as TCGA.

SurGen is among the largest publicly available colorectal cancer WSI datasets, with 1,020 slides from 843 cases, exceeding the combined slide count of TCGA-COAD, TCGA-READ, and CPTAC-CRC. While SurGen’s genomic annotation is focused on specific biomarkers, its scale, resolution, and inclusion of survival data make it particularly well suited for computational pathology research, prognostic modelling, and biomarker classification in colorectal cancer.

## Data Description

This section provides an overview of the SurGen dataset, which includes WSIs and corresponding clinical and genetic data. The dataset is intended to support research in cancer and computational pathology, offering a resource for studying genetic mutations, mismatch repair status, and patient survival outcomes. Below is a detailed description of the data and its collection process.

**Figure 1:**
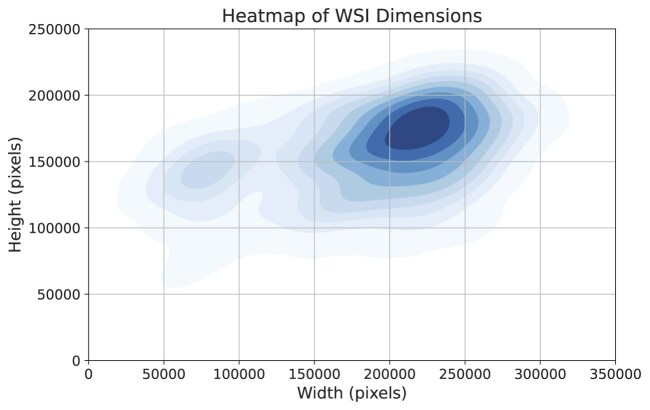
Heatmap of WSI dimensions (in pixels) across the SurGen dataset, illustrating the variability in image sizes due to differing tissue sample areas.

**Figure 2:**
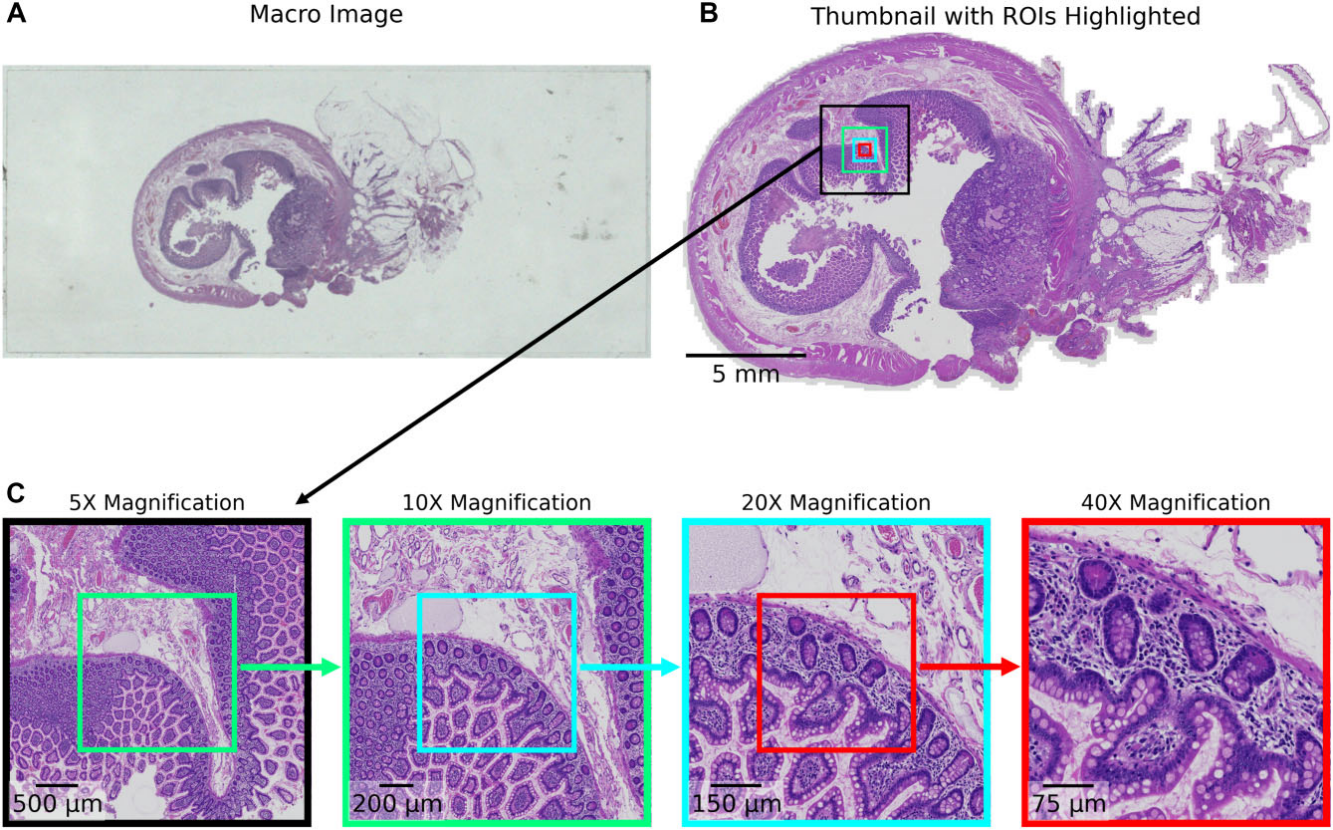
Hierarchical zoom visualisation of case SR1482_T412 with dimensions $242{,}506 \times 134{,}026$ pixels, corresponding to $26{,}974.20 \times 14{,}907.85 \, \, \mu \mathrm{m}$. (A) A low-resolution macro image of the whole slide, providing full anatomical context. (B) Digitised whole-slide image viewed at low magnification. (C) Successive zoom-ins of the selected region from (B), providing increased granularity, enabling detailed examination of tissue structures while retaining the broader context. This hierarchical approach allows comprehensive visual exploration of tissue characteristics at varying scales. In practice, the pyramid levels are typically generated via Gaussian down-sampling to simulate various levels of magnification but enable an immediate interface for retrieving images at varying resolutions.

Each WSI in the SurGen dataset is scanned at 40$\times$ (0.1112 $\, \mu \mathrm{m}$ per pixel) magnification, resulting in ultra-high-resolution images with pixel dimensions averaging $189{,}662\times 156{,}059$ pixels. Figure 1 illustrates the spread of WSI dimensions across the SurGen dataset. The digital pathology images are stored in the CZI file format, which supports hierarchical pyramidal data structures for efficient storage and retrieval. Figure 2 demonstrates the level of granularity accessible via the ultra-high-resolution WSIs.

### Patient demographic

**Figure 3:**
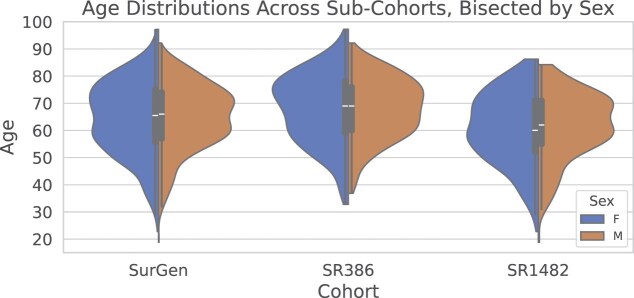
Illustration of the age distributions (in years) of patients in each cohort, split by sex. The width of each violin represents the density of data points at different ages, highlighting the distributions within and across the cohorts.

The SurGen dataset comprises clinical information from 843 cases, with patients ranging from 19 to 97 years of age (mean age = 64.58, SD = $\pm$12.73), as illustrated in Fig. 3. The cohort comprises 46% females and 54% males.

### Patient survival

Survival data are available for the SR386 cohort, providing insights into patient outcomes over a 5-year period following diagnosis. The dataset includes binary labels indicating whether a patient survived beyond the duration of the study, as well as the number of days until death for those who did not. For patients who outlived the study period or whose survival extends beyond the recorded date, their exact number of days until death is not captured, resulting in right-censoring.

**Figure 4:**
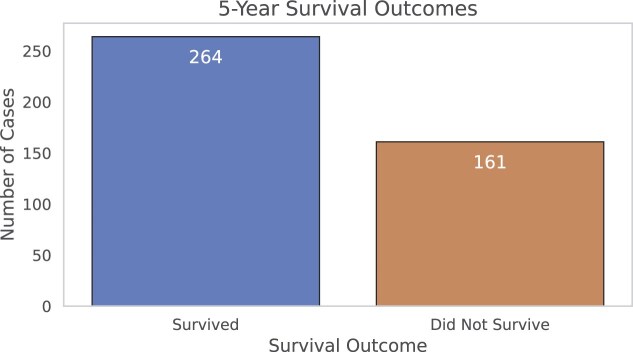
Bar chart depicting the 5-year survival outcomes of the SR386 cohort. The chart shows the number of individuals who survived (*n* = 264) versus those who did not survive (*n* = 161) within the 5-year period following diagnosis. The data exclude instances where survival status was not recorded (NULL values).

Within the SR386 cohort, 161 patients (38%) died during the study period, while 264 patients (62%) were alive at the end of the study period. This distribution provides a general overview of patient outcomes in the cohort. An overview of the binarised 5-year survival outcomes is presented in Fig. 4. CRC was the primary cause of death in 67 out of 161 deceased patients, accounting for 41.61% of all deaths in the cohort.

**Figure 5:**
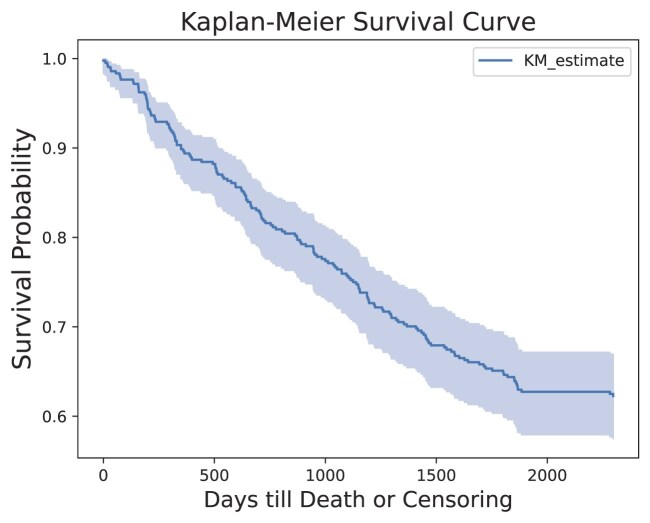
Kaplan–Meier survival curve illustrating estimated survival probabilities over time. Censoring occurred for patients who survived beyond the 5-year study duration, as they were not followed further. The curve reflects the proportion of individuals surviving at each time point, with 95% confidence intervals representing the uncertainty in these estimates.

To visualise the survival probabilities over time, a Kaplan–Meier survival curve was constructed for the SR386 cohort, as shown in Fig. 5. This curve illustrates the proportion of patients surviving at each time point during the study period. The gradual decline in the curve represents the decreasing number of patients alive as time progresses.

**Figure 6:**
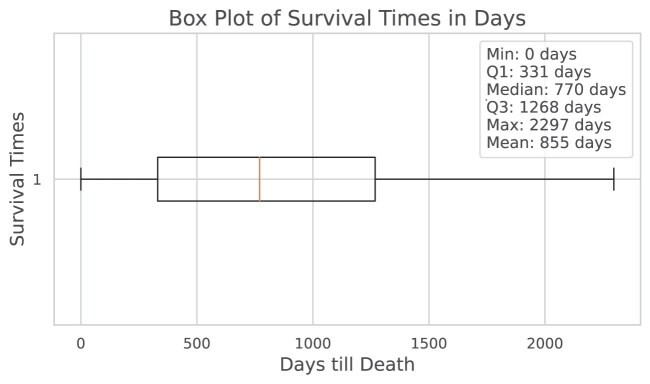
Boxplot showing the distribution of survival times (in days) for cases in the SR386 cohort with recorded days until death. The plot illustrates key summary statistics, including the mean, minimum, first quartile (Q1), median, third quartile (Q3), and maximum survival times.

For the patients who did not survive beyond the study period, we analysed the distribution of their survival times. Figure 6 presents a boxplot summarising key statistics of these survival times in days. The plot shows the minimum, first quartile (Q1), median, third quartile (Q3), maximum, and mean survival times. Specifically, the median survival time was 770 days, indicating that half of the patients who died did so within this number of days postdiagnosis.

**Figure 7:**
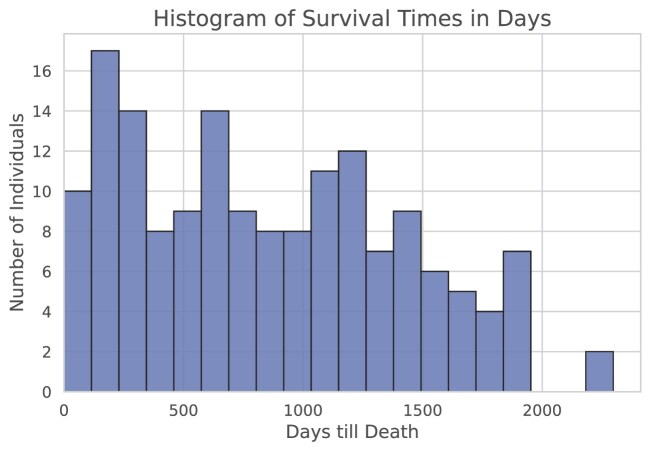
Histogram showing the distribution of survival times (in days) for cases in the SR386 cohort with recorded days until death. The x-axis represents the number of days until death, and the y-axis indicates the number of individuals who died within each time interval.

Additionally, Fig. 7 displays a histogram of the survival times for patients who died within the study period. The histogram shows how many patients died within specific time intervals, providing an overview of the distribution of survival times among these patients.

Due to quality control measures, missing information, or data inconsistencies, certain cases (i.e., 004, 208, 430) have been redacted or marked as “*NULL*” with respect to survival. However, these cases remain in the dataset as they contain valuable genetic information that can be utilised for separate predictive tasks.

### Genetic mutations

**Figure 8:**
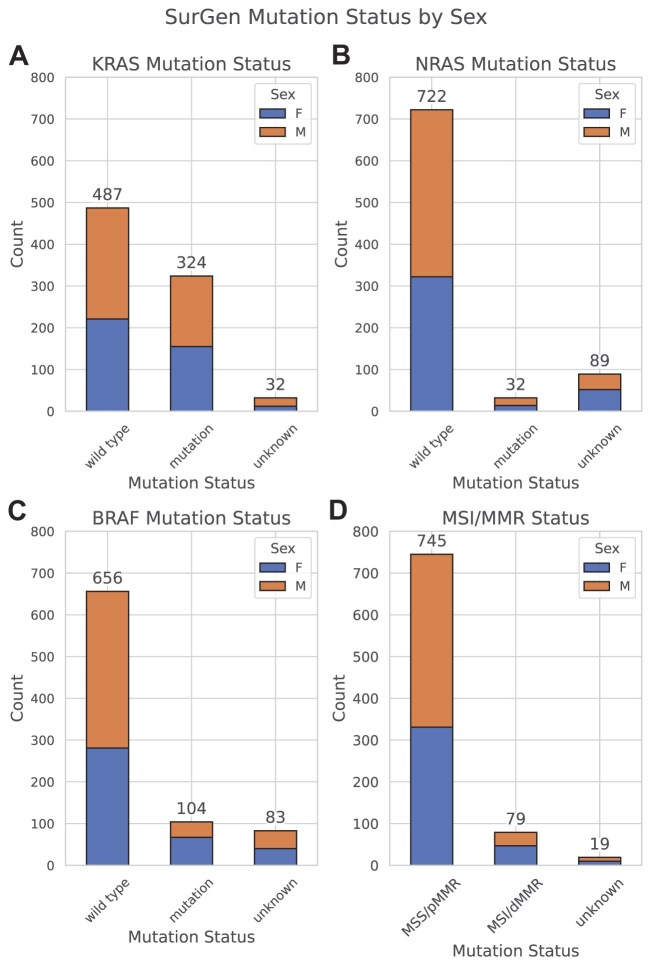
Bar chart depicting mutation prevalence across the SurGen dataset, highlighting mutation status of patients across (A) KRAS, (B) NRAS, (C) BRAF, and (D) MSI/MMR.

The SurGen dataset includes ground-truth labels for key genetic mutations in the *KRAS, NRAS*, and *BRAF* genes, as well as MMR status and/or microsatellite instability (MSI). Figure 8 presents the distribution of these genetic mutations by sex. These genetic markers are crucial for understanding the molecular characteristics of tumours and their potential response to targeted therapies. Below, each mutation is discussed in detail.

#### BRAF mutation

Present in 12.34% of SurGen cases, this aligns with frequencies reported in the literature, which range from 3.5% to 13% [18–22]. BRAF mutations are critical in the MAPK/ERK signaling pathway and are significant targets for therapeutic intervention [23, 24].

#### KRAS mutation

Present in 38.43% of SurGen cases, this is consistent with the range reported in other studies, from 37% to 46.4% [18–21, 25]. KRAS is a proto-oncogene involved in cell signaling pathways that regulate cell growth and death. Mutations in KRAS are often linked to resistance to specific therapies, highlighting the importance of their identification for effective treatment planning [25].

#### NRAS mutation

Observed in 3.80% of SurGen cases, this falls within the range of 2.6% to 9% reported across various studies [19–22]. Like KRAS, NRAS mutations can influence treatment options and prognosis, though NRAS mutations are less common.

### Mismatch repair deficiency and microsatellite instability

Mismatch repair deficiency (dMMR) and MSI are critical genetic features in many cancers, particularly colorectal cancer [26]. dMMR occurs when the mismatch repair system, which normally corrects DNA replication errors, is compromised. This deficiency leads to an accumulation of mutations, particularly in regions of repetitive DNA known as microsatellites. When these microsatellites become unstable due to dMMR, the condition is termed MSI [26, 27].

MSI is a key biomarker used to assess cancer prognosis and predict responses to certain therapies, such as immunotherapy. Tumours exhibiting high levels of MSI (MSI-high) are often associated with a better prognosis and may respond favorably to immune checkpoint inhibitors [28, 29]. Identifying MMR and MSI status is essential for developing targeted treatment strategies and improving patient outcomes.

Importantly, dMMR and MSI are hallmark features of Lynch syndrome (LS), the most common hereditary colorectal cancer predisposition syndrome, accounting for approximately 3% of all colorectal cancers [30, 31]. LS, also known as hereditary nonpolyposis colorectal cancer (HNPCC), is caused by germline mutations in the MMR genes (*MLH1, MSH2, MSH6*, and *PMS2*) [32], leading to a higher risk of developing colorectal cancer and other cancers at a younger age. Identifying patients with dMMR/MSI can therefore aid in diagnosing Lynch syndrome and facilitating genetic counseling [33].

In our study, the assessment of MMR status and MSI status differed between the SR386 and SR1482 cohorts.

#### Assessment of MMR and MSI status in cohorts

While the SR386 cohort reports MMR status assessed through immunohistochemistry (IHC) for key MMR proteins, the SR1482 cohort reports both MMR and MSI status.

##### SR386 cohort

In the SR386 cohort, MMR status was assessed exclusively using immunohistochemistry (IHC) for 2 key MMR proteins: MLH1 and PMS2. Cases were labelled according to the specific loss of expression observed. Primary antibodies against MLH1 and PMS2 were applied, and loss of nuclear staining in tumour cells for any of these MMR proteins was recorded.

##### SR1482 cohort

In the SR1482 cohort, MSI status was determined using either IHC for MMR proteins (MLH1, MSH2, MSH6, PMS2) or PCR-based fragment analysis. For the PCR-based approach, the Promega Oncomate kit was utilised according to the manufacturer’s recommended protocol. Cases were classified as MSI/dMMR if they showed evidence of microsatellite instability through PCR analysis or a loss of protein expression by IHC.

#### Mismatch repair

MMR status is available for most cases, with a distinction between microsatellite-stable (MSS/pMMR) and microsatellite-unstable (MSI/dMMR) tumours within the SR1482 dataset. This information is crucial for identifying patients who might benefit from immunotherapy [27, 28].

#### Microsatellites

MSI is a condition of genetic hypermutability that results from impaired DNA MMR. Identifying MSI is important as it has implications for the prognosis and treatment of cancer.

### Staging

Tumour staging is a critical aspect of cancer diagnosis and treatment planning, providing a framework for assessing the extent of cancer spread within the body. Staging systems help in predicting patient prognosis, guiding treatment decisions, and enabling comparisons across clinical studies and populations [34]. Two widely used staging systems in colorectal cancer are the Dukes’ staging system [35] and the TNM (Tumour, Node, Metastasis) staging system [36], each offering distinct advantages and serving different clinical needs.

The Dukes’ staging system is one of the earliest methods used to classify the extent of colorectal cancer. It is relatively simple and easy to apply, making it useful for broad clinical assessments. However, although it includes stages for lymph node involvement (stage C) and distant metastasis (stage D), its simplicity limits its ability to provide more detailed, granular information on tumour characteristics [37].

The TNM staging system, in contrast, is more detailed and widely applicable across various cancer types. It provides a comprehensive classification based on the size and extent of the primary tumour (T), the involvement of regional lymph nodes (N), and the presence of distant metastasis (M). This system is advantageous for its specificity and adaptability to different cancers, though it can be more complex to use compared to the Dukes’ system.

These staging systems are integral to clinical guidelines, informing treatment strategies such as surgical intervention, chemotherapy, and targeted therapies based on the stage of cancer.

The SurGen dataset includes tumour staging information using both the Dukes’ and TNM staging systems, which are essential for correlating clinical outcomes with tumour progression. Understanding the distribution of these stages across the cohort can offer valuable insights into the disease dynamics within the study population.

#### Tumour staging with Dukes’

The Dukes’ staging system classifies colorectal cancer into 4 stages (A, B, C, and D), based on the extent of tumour invasion and the presence of lymph node involvement or distant metastasis [35]. Stage A represents the earliest form of cancer, confined to the mucosa, while stage D indicates advanced disease with distant metastasis. This system, though less detailed than TNM, provides a quick and accessible way to gauge tumour progression and patient prognosis.

#### TNM staging

The TNM staging system is a more granular approach that classifies cancer based on 3 key components: the size and extent of the primary tumour (T), the involvement of regional lymph nodes (N), and the presence of distant metastasis (M) [38]. Each of these components is assigned a score, and the combination of these scores determines the overall stage of the cancer, ranging from stage 0 (in situ, noninvasive cancer) to stage IV (advanced cancer with distant metastasis).

A comprehensive summary of the SurGen including survival data, genetic mutations, and image properties for both the SR386 and SR1482 subcohorts is provided in Table 3.

**Table 3:** Overview of the SurGen dataset with respective technical, clinical, and mutational characteristic breakdown of the subsets SR386 and SR1482. MSI/MMR ground truth was determined using IHC or PCR.

	SurGen dataset	SR386	SR1482
Origin	Scotland	Scotland	Scotland
Number of cases	843	427	416
Number of WSIs	1020	427	593
WSI file format	.CZI	.CZI	.CZI
Magnification	40×	40×	40×
Microns per pixel (pixel width)	0.1112 $\, \mu \mathrm{m}$	0.1112 $\, \mu \mathrm{m}$	0.1112 $\, \mu \mathrm{m}$
Mean age (SD)	64.58 ($\pm$ 12.73)	67.89 ($\pm$ 12.00)	61.20 ($\pm$ 12.59)
Female, *n* (%)	388 (46.03%)	197 (46.14%)	191 (45.91%)
Male, *n* (%)	455 (53.97%)	230 (53.86%)	225 (54.09%)
MSI/MMR ground truth	PCR/IHC	IHC	PCR/IHC
MSI/dMMR, *n* (%)	79 (9.37%)	32 (7.49%)	47 (11.30%)
MSS/pMMR, *n* (%)	745 (88.37%)	395 (92.51%)	350 (84.13%)
MSI/MMR status unknown, *n* (%)	19 (2.25%)	0 (0%)	19 (4.57%)
Five-year survival (true), *n* (%)	264 (31.32%)	264 (61.83%)	0 (0%)
Five-year survival (false), *n* (%)	162 (19.22%)	162 (37.94%)	0 (0%)
Five-year survival (unreported), *n* (%)	417 (49.47%)	1 (0.23%)	416 (100%)
BRAF mutation, *n* (%)	104 (12.34%)	47 (11.00%)	57 (13.70%)
BRAF wild type, *n* (%)	656 (77.82%)	379 (88.76%)	277 (66.59%)
BRAF status unknown, *n* (%)	83 (9.85%)	1 (0.23%)	82 (19.71%)
KRAS mutation, *n* (%)	324 (38.43%)	147 (34.43%)	177 (42.55%)
KRAS wild type, *n* (%)	487 (57.77%)	266 (62.30%)	221 (53.12%)
KRAS status unknown, *n* (%)	32 (3.80%)	14 (3.26)	18 (4.33%)
NRAS mutation, *n* (%)	32 (3.80%)	16 (3.75%)	16 (3.85%)
NRAS wild type, *n* (%)	722 (85.65%)	399 (93.44%)	323 (77.64%)
NRAS status unknown, *n* (%)	89 (10.56%)	12 (2.81%)	77 (18.51%)

### Data collection

#### Tissue sample preparation

Samples underwent formalin-fixed, paraffin-embedding (FFPE) processing. This involved fixing tissue specimen in formalin to preserve cellular structures and proteins, followed by embedding the samples in paraffin wax.

Once FFPE samples were prepared, they were processed using a microtome set to section at 5 $\, \mu \mathrm{m}$ before being laid onto a glass slide. These slides were then subjected to routine hematoxylin and eosin (H&E) staining prior to their digitisation.

Slides were first immersed in hematoxylin, which stains the cell nuclei blue-purple. Following a rinse, slides were stained with eosin, which stains the cytoplasm and extracellular matrix pink. After staining, the slides underwent a dehydration process involving graded alcohols and xylene. Coverslips were subsequently applied with a mounting medium to preserve the stained sections.

#### Tissue sample digitisation

**Figure 9:**
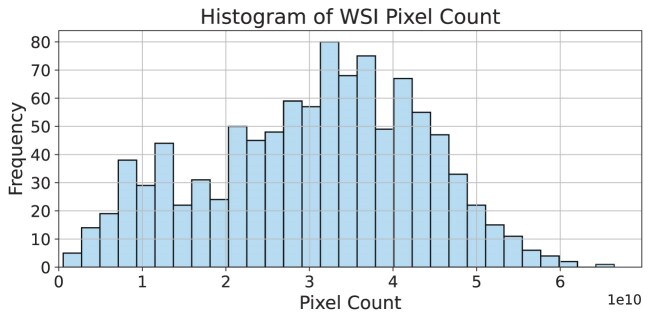
Histogram illustrating the scale of SurGen whole-slide images with respect to the number of pixels per image. The x-axis represents the total number of pixels in each image (in tens of billions, $1\times 10^{10}$), while the y-axis indicates the frequency of occurrence of images within each bin. The distribution shows the variability in the sizes of whole-slide images across the dataset.

Prepared slides were digitised on-site using a ZEISS Axio Scan.Z1 Microscopy Slide Scanner at 40$\times$ magnification, equipped with a Plan-Apochromat 40×/0.95 Korr M27 objective lens. This combination produces a pixel size of $0.1112\, \mu \mathrm{m}$. The scans were performed using ZEN 2.6 (blue edition) software, capturing brightfield images with controlled transmitted light illumination. Digitised images were saved in 24-bit BGR format (BGR24) with a pixel size of $0.1112\, \mu \mathrm{m}$. A multiresolution pyramidal image structure was generated, with each subsequent layer down-sampled by a factor of 2 relative to the previous layer, using Gaussian filtering to maintain image quality. Figure 9 illustrates the WSI pixel counts across the SurGen dataset.

While this imaging setup yields an ultra-high resolution of $0.1112\, \, \mu \mathrm{m}$ per pixel, it is important to note that pixel size is not standardised across all digital pathology platforms. Scanners from other vendors (e.g., Philips, Hamamatsu, Leica) may report the same nominal magnification (e.g., 40$\times$), yet produce images with coarser resolutions, typically around $0.25\, \, \mu \mathrm{m}$ per pixel, due to differences in objective lens, camera sensors, and internal optics. As such, magnification alone is an imprecise descriptor of image resolution. Reporting the true microns-per-pixel (MPP) value provides a more objective and reproducible measure of resolution, enabling proper normalisation across datasets acquired using differing hardware configurations.

For both cohorts, each whole-slide image was digitised from the same FFPE tissue block used for biomarker assessment, ensuring correspondence between the histological and molecular data.

#### DNA sequencing

Next-generation sequencing (NGS) was performed to determine the mutation status of KRAS, NRAS, and BRAF using the Ion Torrent Cancer Hotspot Panel v2 (Thermo Fisher Scientific), following the manufacturer’s protocol.

### Data curation and quality control

To ensure the quality and reliability of the SurGen dataset, we implemented several data curation and quality control measures.

#### Slide quality assessment

All WSIs were reviewed by specialised laboratory personnel trained in the preparation of tissue samples for microscopic examination. Each slide was assessed for staining quality, focus, and absence of artifacts. Slides that did not meet acceptable standards were rescanned or reprepared to improve image quality.

#### Data alignment and consistency

To maintain data integrity and maximise the utility of the dataset, we carefully matched each WSI with its corresponding clinical and genetic data. WSIs without any matching clinical data were excluded from the dataset, as clinical context is essential for meaningful analyses. However, clinical data entries were retained even if some fields were incomplete, provided they had a corresponding WSI. This approach ensured that all included WSIs had associated clinical information, enhancing the dataset’s applicability while acknowledging that some clinical records might have missing data points.

#### Anonymisation and ethical considerations

Patient confidentiality was prioritized throughout the curation of the SurGen dataset. In line with contemporary data ethics in computational pathology [5, 39], we implemented deidentification protocols to ensure privacy while maximising data utility. Recognise that medical images potentially carry the risk of reidentification when combined with external data sources, our anonymisation strategy involved the removal or redaction of potentially identifiable information, including dates of diagnosis, date of death, and treatment details.

### Data use

Researchers can interact with the WSIs using tools such as OpenSlide [40], pylibCZIrw [41], and Bioformats [42]. The images are saved in a hierarchical pyramidal format, facilitating efficient viewing and processing at multiple resolutions. Software such as QuPath [43], Fiji [44], ImageJ [45], and others can be used to visualise and analyse these images.

**Figure 10:**
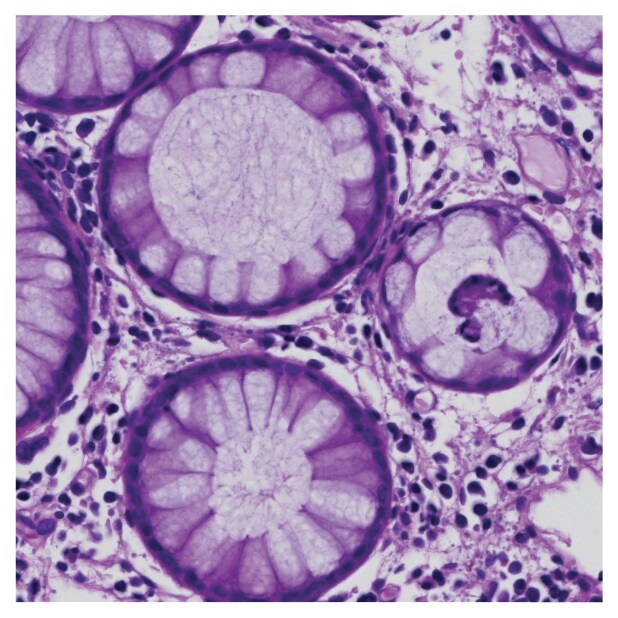
Example 2,048 $\times$ 2,048-pixel tile extracted from the centre of a whole-slide image (WSI) using pylibCZIrw. This patch, from case SR1482_T232, illustrates the fine detail captured at 40× (0.1112$\, \mu \mathrm{m}$ per pixel) resolution. Extraction of patches, as demonstrated here, is an essential step in SOTA preprocessing pipelines.

To illustrate SurGen’s practical utility, we provide a simple Python example for extracting a region of interest from a whole-slide image. A Python script was implemented using pylibCZIrw (see Supplementary File S1). The script illustrates the process of identifying the centre of the WSI and extracting a 2,048 $\times$ 2,048-pixel region of interest (ROI) at full resolution. The extracted tile (Fig. 10) provides a high-resolution view from the WSI, showcasing the potential for downstream analyses or tasks, such as patch-level feature extraction or visualisation.

### Data reuse potential

The SurGen dataset offers extensive opportunities for researchers in computational pathology and oncology. Its comprehensive collection of WSIs, coupled with genetic and other clinical annotations, makes it a valuable resource for various applications.

First, the dataset can be utilised to train machine learning models for predicting MMR status and MSI. Given that existing publicly available datasets focusing on MSI/MMR prediction are limited, SurGen fills a crucial gap. Researchers can leverage this dataset to develop and validate models that may enhance diagnostic accuracy and inform treatment strategies, particularly in colorectal cancer, in which MSI status is a key prognostic and therapeutic marker.

Second, SurGen provides a rich resource for training models aimed at genomic mutation prediction, specifically for mutations in the KRAS, NRAS, and BRAF genes. Expanding the quantity of publicly available datasets with such detailed genetic information is immensely valuable, as it enables the development of models that can predict genetic mutations from histopathological images. This can potentially streamline the diagnostic process by reducing the need for costly and time-consuming genetic testing.

Furthermore, the high-quality WSIs in the SurGen dataset make it suitable for training foundation models in digital pathology. Existing works have demonstrated that the performance of these models improves with the availability of larger and more diverse datasets [46–48]. By contributing to the training of such models, SurGen can aid in advancing the field of computational pathology, facilitating the development of algorithms that are more robust and generalisable.

The dataset’s versatility allows it to be used in multiple ways:

- Researchers may choose to utilise the SR386 or SR1482 subsets independently, depending on their specific research questions. For instance, studies focusing on primary tumour characteristics and survival can benefit from the SR386 cohort’s valuable genetic and survival data.
- Alternatively, the entire SurGen dataset can be employed collectively as a larger cohort for tasks such as staging or genetic slide-level classification, benefiting from the increased sample size and additional diversity from metastatic tumour sites.
- SurGen also holds significant potential as an external validation set for existing studies and algorithms. External validation is essential for assessing the generalisability of predictive models, and the dataset’s comprehensive annotations make it particularly suitable for this purpose [49].

To support systematic benchmarking and methodological comparisons, we provide example stratified data-splits for the SR386 subset (see Table 4), as well as for the SR1482 subset and the combined SurGen dataset. Although detailed stratifications are only presented here for SR386, equivalent splits for the full SurGen dataset and the SR1482 subset are available in the accompanying GitHub repository. Each split is stratified to ensure balanced distributions of key variables such as genetic mutations, MMR/MSI status, and survival metrics. These data partitions establish a standardised, transparent framework for evaluating model performance and reproducibility when utilising the SurGen dataset.

**Table 4:** Breakdown of SR386 SurGen colorectal cohort data distribution for train, validate, and test sets. This stratification may act as an effective starting point for future analysis. Each patient has precisely 1 associated whole-slide image. This breakdown was stratified by age, sex, MSI/MMR, RAS (KRAS or NRAS), and BRAF mutation.

Category	Total (SR386)	Train	Validate	Test
Origin	Scotland	Scotland	Scotland	Scotland
WSI file format	CZI	CZI	CZI	CZI
Magnification	$\times$ 40	$\times$ 40	$\times$ 40	$\times$ 40
Microns per pixel (pixel width)	0.1112$\, \mu \mathrm{m}$	0.1112$\, \mu \mathrm{m}$	0.1112$\, \mu \mathrm{m}$	0.1112$\, \mu \mathrm{m}$
Number of patients	423 (100%)	255 (60%)	84 (20%)	84 (20%)
Mean age at diagnosis (SD)	67.89 ($\pm$ 11.97)	67.98 ($\pm$ 12.12)	67.71 ($\pm$ 11.40)	67.80 ($\pm$ 12.20)
Male, *n* (%)	228 (54%)	138 (54.1%)	46 (54.7%)	44 (52.3%)
Female, *n* (%)	195 (46.0%)	117 (45.8%)	38 (45.2%)	40 (47.6%)
MSS/pMMR, *n* (%)	391 (92%)	235 (92%)	78 (93%)	78 (93%)
MSI/dMMR, *n* (%)	32 (8%)	20 (8%)	6 (7%)	6 (7%)
Five-year survival (true), *n* (%)	159 (38%)	100 ( 39%)	30 (36%)	29 (35%)
Five-year survival (false), *n* (%)	264 (62%)	155 (61%)	54 (64%)	55 (65%)
RAS mutation, *n* (%)	158 (37%)	97 (38%)	31 (37%)	30 (36%)
RAS wild type, *n* (%)	265 (63%)	158 (62%)	53 (63%)	54 (64%)
BRAF mutation, *n* (%)	47 (11.1%)	29 (11.4%)	9 (10.7%)	9 (10.7%)
BRAF wild type, *n* (%)	375 (88.6%)	225 (88.2%)	75 (89.2%)	75 (89.2%)
BRAF fail, *n* (%)	1 (0.2%)	1 (0.4%)	0 (0%)	0 (0%)

An example of the dataset’s utility is demonstrated in a study that explored the feasibility of digital pathology foundation models on the SR386 cohort. Using the UNI model [46], which was benchmarked against various other pathology-pretrained foundation models and an ImageNet-pretrained ResNet-50 [50], this work achieved a test area under the receiver operating characteristic (AUROC) curve of 0.7136 for slide-level classification of MMR status [51]. This underscores the dataset’s potential in facilitating advanced machine learning applications.

## Analysis

To further demonstrate the utility of the SurGen dataset, we conducted an experiment combining the SR386 and SR1482 cohorts to predict MMR status using a machine learning model. We utilised the existing training, validation, and test splits from each cohort and merged them to form unified training, validation, and test sets. This approach ensured that the combined SurGen dataset adhered to the 60:20:20 ratio for training, validation, and testing, respectively, while maintaining a balanced representation of mutation statuses across each split. By leveraging the predefined splits from both cohorts, we eliminated the need to generate a separate third split. The splits used in this experiment are provided in CSV format to ensure reproducibility.

### Feature extraction

A range of pretrained foundation models have been developed for histopathological image analysis, each leveraging diverse self-supervised learning techniques and trained on extensive collections of WSIs. These models have demonstrated considerable success in capturing nuanced histopathological features [46, 52–73].

For this study, we employed the UNI foundation model [46] for feature extraction from WSIs. UNI was selected due to its robust performance in representing histopathological features relevant to microsatellite instability (MMR status) within the SR386 cohort [51]. The model is a self-supervised vision encoder trained on over 100,000 H&E-stained WSIs across a wide variety of tumour sites, thereby providing a comprehensive representation of tissue morphology.

**Figure 11:**
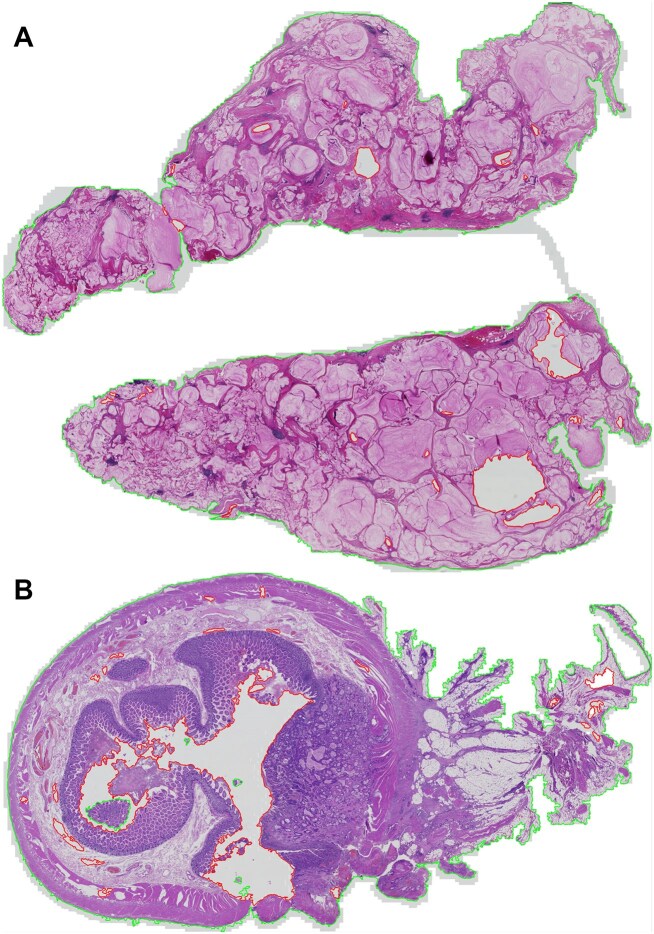
Background subtraction from (A) case SR148_T230, peritoneal biopsy and (B) case SR148_T412, small bowel resection. Tissue area is outlined in green with holes and background delineated in red.

Feature extraction was performed on nonoverlapping 224 × 224 tissue patches at a scale of 1.0 MPP, yielding a 1,024-dimensional embedding for each patch. Background subtraction was applied, as illustrated in Fig. 11. The entire process of patch extraction and feature embedding required 110.55 hours, utilising a single NVIDIA V100 32 GB GPU. For convenience and reproducibility, these embeddings are made available online.

### Model training and evaluation

**Figure 12:**
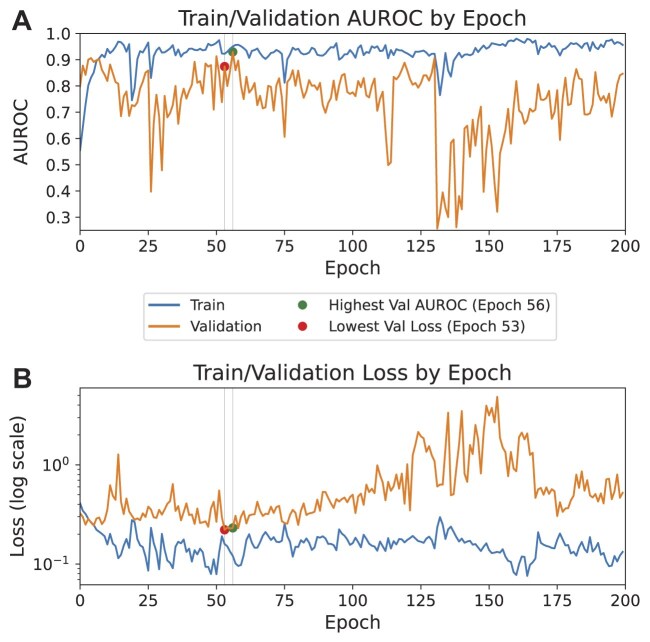
Train/validation AUROC curve and loss by epoch. (A) The train and validation AUROC curve progression over 200 epochs, with markers indicating the highest validation AUROC curve and the epoch with the lowest validation loss. (B) The train and validation loss on a log scale, highlighting the convergence and divergence trends, with markers indicating key performance metrics such as the lowest validation loss and the epoch with the highest AUROC curve.

**Figure 13:**
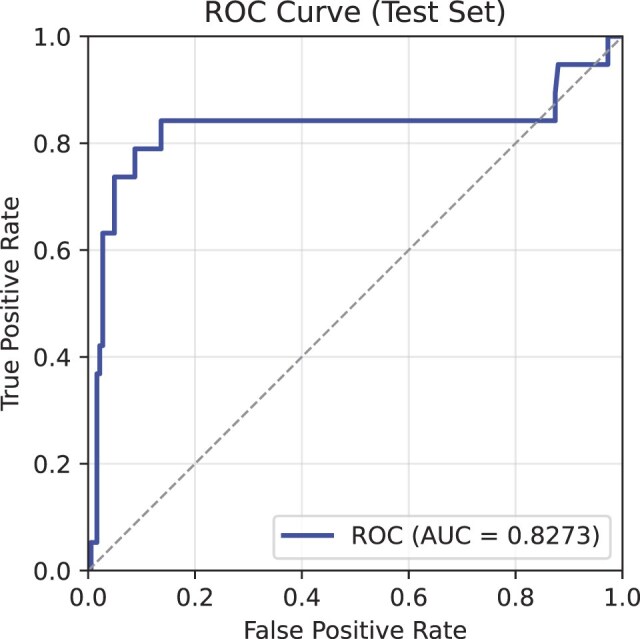
Receiver operating characteristic (ROC) curve for the model, showing an AUROC curve of 0.8273. The curve plots the true-positive rate (sensitivity) against the false-positive rate (1 − specificity) across various classification thresholds, with an AUROC curve of 1 representing perfect classification and 0.5 indicating random chance.

A Transformer [74]–based classifier was trained using the extracted UNI patch embeddings. Details of the model parameters are provided in Table 5. Performance was evaluated primarily using the AUROC curve metric. Training was conducted on a single NVIDIA V100 32 GB GPU, completing in 3 hours, 2 minutes, and 36 seconds. The progression of the training and validation AUROC curve, as well as the loss over 200 epochs, is shown in Fig. 12. This figure highlights key performance metrics, including the highest validation AUROC curve and the lowest validation loss. Preliminary results indicate a validation AUROC curve of 0.9297 and a test AUROC curve of 0.8273 (see Fig. 13 for test AUROC curve). These results demonstrate the model’s potential for accurately predicting MMR status from WSIs. Future work could focus on fine-tuning hyperparameters and exploring the integration of state-of-the-art (SOTA) pretrained feature extractors to further improve model performance.

**Table 5:** Summary of model parameters used for MMR/MSI classification.

Parameter	Value
**Task**	MMR/MSI detection
**Cohort**	SurGen
**Feature extractor**	*UNI*
**Patch size**	224 × 224
**Microns per pixel (MPP)**	1.0
**Embedding dimension** ($d_{\text{model}}$)	512
**Transformer encoder layers** ($L$)	2
**Attention heads** ($H$)	2
**Feedforward dimension** ($d_{\text{ff}}$)	2,048
**Activation function**	ReLU
**Dropout rate**	0.15
**Layer norm epsilon**	$1 \times 10^{-5}$
**Loss function**	BCEWithLogitsLoss
**Optimiser**	Adam
**Learning rate**	$1 \times 10^{-4}$
**Batch size**	1
**Epochs**	200
**Automatic mixed precision (AMP)**	True
**GPU**	NVIDIA V100 32 GB

#### Model architecture

The model consists of a feature embedding layer, a transformer encoder, an aggregation layer, and a classification head. The feature extractor used was the UNI model, which produced 1,024-dimensional feature vectors for each patch. These were mapped to a 512-dimensional latent space via a fully connected layer and ReLU activation. The transformer encoder consisted of 2 layers, each with 2 attention heads, and a feedforward dimension of 2,048. After passing through the transformer encoder, the patch features were mean-pooled to obtain a slide-level feature representation. A final fully connected layer then mapped the pooled feature vector to the number of classes (for multiclass tasks) or to a single output (for binary classification). The full architecture configuration is detailed in Table 5.

#### Training configuration

The model was trained using patch embeddings extracted from WSIs at 1.0$\mu /pixel$ per pixel, with patch sizes of 224 × 224. As the number of patches per WSI varied based on the specimen size, we processed all patches in a single forward pass. The training was conducted on a single NVIDIA V100 32 GB GPU, with a batch size of 1 and a learning rate of $1 \times 10^{-4}$. The Adam optimise was used, and binary cross-entropy with logits loss (BCEWithLogitsLoss) was applied for binary classification tasks. No class balancing was performed. The model was trained for 200 epochs, and automatic mixed precision (AMP) was enabled to optimise GPU usage. Table 5 provides a summary of the key parameters used in the training process.

### Experiment results

The results underscore the strong utility of the SurGen dataset for developing predictive models in computational pathology. Compared with the previous work [51], which achieved an AUROC curve of 0.7136 on the smaller SR386 subset, the higher test AUROC curve of 0.8273 observed here suggests that SurGen’s broader scope and consistently high-quality images may foster more robust model performance. Although additional investigation is necessary to establish whether this improvement stems primarily from the expanded sample size and greater tumour heterogeneity, these findings emphasize the importance of a large, well-curated dataset for accurate MMR status prediction.

**Figure 14:**
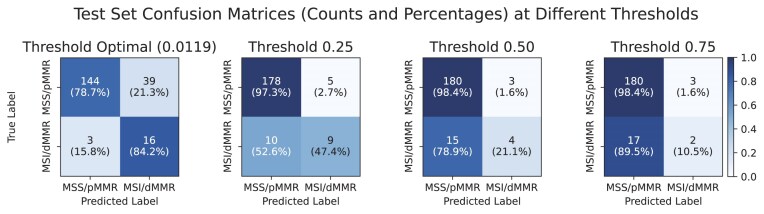
Confusion matrices for MMR status prediction at various classification thresholds on the test set. The confusion matrices show the classification results for MMR status prediction across 4 different decision thresholds (0.0119, 0.25, 0.50, and 0.75). Threshold 0.0119 represents the point at which 95% sensitivity on the validation set is reached. Each matrix shows the number and percentage of correct and incorrect predictions for the microsatellite-stable/proficient MMR (MSS/pMMR) and microsatellite-instable/deficient MMR (MSI/dMMR) classes.

The Transformer-based model demonstrated strong performance in predicting MMR status, achieving an AUROC curve of 0.9297 on the validation set and 0.8273 on the test set. To illustrate how well the model balances sensitivity and specificity, Fig. 14 shows the confusion matrices at 4 thresholds, optimal (0.0119), 0.25, 0.50, and 0.75, providing a detailed breakdown of the model’s classification performance. These matrices help reveal trade-offs between true positives and false positives under different decision criteria and indicate how threshold selection can be tailored for particular clinical aims. For instance, the 0.0119 threshold achieves 95% sensitivity on the validation set, which may be important in early-stage colorectal cancer to minimise the chance of missing diseased cases.

## Discussion

In this study, we introduce the SurGen dataset, a comprehensive collection of 1,020 H&E-stained WSIs from 843 colorectal cancer cases with detailed genetic and clinical annotations. This dataset addresses the critical need for extensive, high-quality datasets in computational pathology to advance cancer diagnosis and treatment. To demonstrate its utility, we developed a machine learning model capable of predicting MMR status from the SurGen dataset, achieving a test AUROC curve of 0.8273 with no hyperparameter tuning. This performance demonstrates a significant improvement over previous efforts, which, despite extensive hyperparameter optimisation on the SR386 subset, achieved an AUROC curve of only 0.7136 [51]. This further motivates the need for large and comprehensive WSI datasets to conduct robust and generalisable computational pathology research. The SurGen dataset directly addresses this need by providing a resource that complements existing datasets with its high-resolution WSIs, extensive annotations, and consistent imaging quality.

Unlike many existing datasets, which often suffer from inconsistent image quality that results in users removing subsets of cases [5, 75–78], SurGen offers over 1,000 consistently high-quality WSIs. This ensures researchers can develop and evaluate models on a dataset that reflects real-world high-quality diagnostic conditions.

The SurGen dataset’s extensive annotations and high-quality WSIs make it a valuable resource for developing foundational artificial intelligence models, enabling transfer learning and domain-specific fine-tuning across a wide range of computational pathology tasks.

By providing a robust foundation for algorithm development, the SurGen dataset supports ongoing efforts to personalise cancer diagnosis and treatment strategies at a global scale.

## Potential Implications

The SurGen dataset has the potential to impact various areas of cancer research and computational pathology.

In computational pathology, the dataset could serve as a valuable resource for developing and evaluating machine learning algorithms. The diversity of tumour sites and genetic annotations could help in creating more generalisable and robust models. One potential avenue for future research might be to integrate and compare tools such as GrandQC [79] and others [75, 80, 81], which can aid in performing quality control analysis and, in some instances, precise tissue segmentation. Additional research could be developed with the aim of exploring the clinical tabular data with respect to tumour staging, genetic mutation, and survival analysis. Further work could aim to integrate all of these aspects on top of a computer vision model.

While the dataset originates from a single geographical region, it offers an opportunity to study population-specific cancer characteristics. Comparing SurGen with datasets from other regions may help identify global cancer disparities and inform international research. SurGen, in combination with other international datasets, may offer a broad and comprehensive resource that enhances the generalisability of computational models across diverse populations. This integration can facilitate the development of more robust diagnostic tools that are effective in varied clinical settings, ultimately contributing to a more unified and global approach to cancer diagnosis and treatment. Additionally, leveraging SurGen alongside other datasets can support large-scale studies, enabling researchers to validate findings across different cohorts and improve the reliability of predictive models. Such efforts can drive advancements in personalised medicine, ensuring that computational pathology solutions are both accurate and universally applicable.

The SurGen dataset has already been adopted in an independent large-scale benchmark by [82]. In that study, a survival prediction pipeline was trained and evaluated across 7 public cohorts that span multiple tumour types. The resulting top CoXNet [83] and Supervised Cindices are reproduced in Table 6. SurGen ranks in the upper half of all cohorts and outperforms several CPTAC datasets of comparable size, thereby providing external evidence of its prognostic signal.

**Table 6:** Comparison of survival prediction performance across datasets. Results are reported as overall survival C-index (mean $\pm$ SE) over 5-fold cross-validation. Data are derived and collated from [82].

Dataset	Patients	CoxNet	Supervised
BOEHMK [84]	183	0.541 $\pm$ 0.013	0.575 $\pm$ 0.049
**SURGEN** (ours)	144	0.638 $\pm$ 0.014	0.632 $\pm$ 0.022
CPTAC-LUAD [85]	105	0.614 $\pm$ 0.032	0.576 $\pm$ 0.046
CPTAC-HNSC [85]	102	0.631 $\pm$ 0.076	0.514 $\pm$ 0.011
CPTAC-PDAC [85]	97	0.616 $\pm$ 0.031	0.611 $\pm$ 0.042
CPTAC-CCRCC [85]	94	0.675 $\pm$ 0.063	0.693 $\pm$ 0.043
MBC [86,87]	75	0.550 $\pm$ 0.027	0.608 $\pm$ 0.030

Ultimately, the SurGen dataset has the potential to accelerate innovations in cancer diagnostics, enhance treatmentpersonalisation, and contribute to reducing the global burden of colorectal cancer.

## Availability of Source Code and Requirements

Source code for data processing and stratification, background subtraction, feature extraction, model training, and evaluation is available via the project page link below.

- Project name: SurGen-Dataset
- Project page: https://github.com/CraigMyles/SurGen-Dataset
- Operating system: Ubuntu 20.04 LTS
- Programming language: Python
- Other requirements: Pytorch, pylibCZIrw, pandas, NumPy
- Source code license: GPL-3.0
- Software Heritage archive: accession swh:1:snp:39dc17fe24087df9ebae119d77d17398aa1ee25a [88]

## Compute Resource

In accordance with the recommended minimum documentation for computation time reporting [94], we have detailed the hardware specifications, computation time, and operating system used during the experiments.

Feature extraction from WSIs using the UNI foundation model took 2 days, 10 hours, 12 minutes, and 35 seconds on a system equipped with a Dual 20-Core Intel Xeon E5-2698 v4 2.2 GHz and a single NVIDIA Tesla V100 32GB GPU. Model training was completed in 3 hours, 2 minutes, and 36 seconds under the same hardware conditions.

- System: NVIDIA DGX-1
- Operating system: Ubuntu 20.04 LTS
- CPU: Dual 20-Core Intel Xeon E5-2698 v4 2.2 GHz
- GPU: NVIDIA Tesla V100 32GB (Utilised 1 of 8 available)
- RAM: 512 GB DDR4 RAM

## Additional Files

**Supplementary File S1:** Python code demonstrating how to extract a tile from the centre of a WSI using in Python 3.8.13 and pylibCZIrw v4.1.3. This example illustrates how to interact with high-resolution pathology images in CZI format. This method can be easily expanded to tessellate over an entire whole slide image for the purpose of patch-level feature extraction.

giaf086_Supplemental_Files

giaf086_Authors_Response_To_Reviewer_Comments_Revision_1

giaf086_Authors_Response_To_Reviewer_Comments_Revision_2

giaf086_Authors_Response_To_Reviewer_Comments_Revision_3

giaf086_GIGA-D-24-00594_Original_Submission

giaf086_GIGA-D-24-00594_Revision_1

giaf086_GIGA-D-24-00594_Revision_2

giaf086_GIGA-D-24-00594_Revision_3

giaf086_Reviewer_1_Report_Original_Submission Prof. Yuri Tolkach -- 2/2/2025

giaf086_Reviewer_2_Report_Original_Submission Dr. Oliver Lester Saldanha -- 10/2/2025

giaf086_Reviewer_2_Report_Revision_1 Dr. Oliver Lester Saldanha -- 6/4/2025

## Abbreviations

AUROC: area under the receiver operating characteristic; BRAF: v-Raf murine sarcoma viral oncogene homolog B; CRC: colorectal cancer; CZI: Carl Zeiss Image (file format); dMMR: deficient mismatch repair; FFPE: formalin-fixed, paraffin-embedded; H&E: hematoxylin and eosin; IHC: immunohistochemistry; KRAS: Kirsten rat sarcoma viral oncogene homolog; MMR: mismatch repair; MSI: microsatellite instability; MSS: microsatellite stable; NGS: next-generation sequencing; NRAS: neuroblastoma RAS viral oncogene homolog; TNM: Tumour, Node, Metastasis; WSI: whole-slide image.

## Consent for Publication

Not applicable. This manuscript does not contain any individual person’s data in a form that would require explicit consent for publication. Comprehensive efforts have been made to ensure patient anonymity. Identifiable information, such as dates of diagnosis, treatment details, and other specifics that could link specimens back to individual patients, have been removed. Furthermore, the dataset has undergone rigorous deidentification processes to aid the prevention reidentification.

## Data Availability

The dataset supporting this article is available in the European Molecular Biology Laboratory European Bioinformatics Institute (EMBL-EBI) BioImage Archive [89] repository and is available via [90] or accessible from within the GitHub README file. Patch embeddings generated during the preprocessing stages using the UNI foundation model have also been made available to reduce the barrier for entry to researchers wishing to utilise this dataset [91]. Dome-ML (Data, Optimisation, Model and Evaluation in Machine Learning) annotations are available via the DOME registry under accession vuknweu17e [92]. Supporting data are available via the *GigaScience* database, GigaDB [93].
