# Supplementary material for: SurGen: 1020 H&E-stained whole-slide images with survival and genetic markers
Source: Gigascience. 2025 Oct 8;14:giaf086. doi: 10.1093/gigascience/giaf086 (PMC12569769; doi:10.1093/gigascience/giaf086)
Supplement: giaf086_Supplemental_Files [file giaf086_supplemental_files.zip › Supplementary file.pdf]

Supplementary File S1 – Example Python script

```python
from pylibCZIrw import czi
# Path to the CZI file
path = "./SR1482_40X_HE_T232_01.czi"

# Open the CZI file and read a patch from the center
with czi.open_czi(path) as czidoc:
    bbox = czidoc.total_bounding_box
    x_min, x_max = bbox['X']
    y_min, y_max = bbox['Y']

    patch_size = 2048

    # Calculate the center coordinates
    center_x = (x_min + x_max) // 2
    center_y = (y_min + y_max) // 2

    # Calculate ROI coordinates
    roi_x = center_x - patch_size // 2
    roi_y = center_y - patch_size // 2

    # Read the patch at full resolution
    patch = czidoc.read(
        roi=(roi_x, roi_y, patch_size, patch_size),
        zoom=1.0 # Render at full (40X) resolution
    )
```

Listing 1. Python code demonstrating how to extract a tile from the centre of a WSI using in `Python 3.8.13` and `pylibCZIrw v4.1.3`. This example illustrates how to interact with high-resolution pathology images in CZI format. This method can be easily expanded to tessellate over an entire whole slide image for the purpose of patch-level feature extraction.
