# Supplementary material for: SurGen: 1020 H&E-stained whole-slide images with survival and genetic markers
Source: Gigascience. 2025 Oct 8;14:giaf086. doi: 10.1093/gigascience/giaf086 (PMC12569769; doi:10.1093/gigascience/giaf086)

GigaScience

SurGen: 1020 H&E-stained Whole Slide Images With Survival and Genetic Markers

--Manuscript Draft--

**Manuscript Number:**	GIGA-D-24-00594R2
**Full Title:**	SurGen: 1020 H&E-stained Whole Slide Images With Survival and Genetic Markers
**Article Type:**	Data Note
**Funding Information:**	UK Research and Innovation (104690) — Not applicable
**Abstract:**	Background: Cancer remains one of the leading causes of morbidity and mortality worldwide. Comprehensive datasets that combine histopathological images with genetic and survival data across various tumour sites are essential for advancing computational pathology and personalised medicine. Results: We present SurGen, a dataset comprising 1,020 H&E-stained whole slide images (WSIs) from 843 colorectal cancer cases. The dataset includes detailed annotations for key genetic mutations (KRAS, NRAS, BRAF) and mismatch repair status, as well as survival data for 426 cases. We illustrate SurGen's utility with a proof-of-concept model that predicts mismatch-repair status directly from WSIs, achieving a test AUROC of 0.8316. These preliminary results underscore the dataset's potential to facilitate research in biomarker discovery, prognostic modelling, and advanced machine learning applications in colorectal cancer and beyond. Conclusions: SurGen offers a valuable resource for the scientific community, enabling studies that require high-quality WSIs linked with comprehensive clinical and genetic information on colorectal cancer. Our initial findings affirm the dataset's capacity to advance diagnostic precision and foster the development of personalised treatment strategies in colorectal oncology. Data available online: https://doi.org/10.6019/S-BIAD1285.
**Corresponding Author:**	Craig Gregor Gilchrist Myles<br>University of St Andrews<br>St Andrews, UNITED KINGDOM
**Corresponding Author Secondary Information:**	
**Corresponding Author's Institution:**	University of St Andrews
**Corresponding Author's Secondary Institution:**	
**First Author:**	Craig Gregor Gilchrist Myles
**First Author Secondary Information:**	
**Order of Authors:**	Craig Gregor Gilchrist Myles
	In Hwa Um
	Craig Marshall
	David Cameron Christopher Harris-Birtill
	David James Harrison
**Order of Authors Secondary Information:**	
**Response to Reviewers:**	Completed requirements.
**Additional Information:**	
**Question**	**Response**
Are you submitting this manuscript to a special series or article collection?	No
**Experimental design and statistics**	Yes

Powered by Editorial Manager® and ProduXion Manager® from Aries Systems Corporation

Full details of the experimental design and statistical methods used should be given in the Methods section, as detailed in our Minimum Standards Reporting Checklist. Information essential to interpreting the data presented should be made available in the figure legends.<br><br>Have you included all the information requested in your manuscript?	
**Resources**<br><br>A description of all resources used, including antibodies, cell lines, animals and software tools, with enough information to allow them to be uniquely identified, should be included in the Methods section. Authors are strongly encouraged to cite Research Resource Identifiers (RRIDs) for antibodies, model organisms and tools, where possible.<br><br>Have you included the information requested as detailed in our Minimum Standards Reporting Checklist?	Yes
**Availability of data and materials**<br><br>All datasets and code on which the conclusions of the paper rely must be either included in your submission or deposited in publicly available repositories (where available and ethically appropriate), referencing such data using a unique identifier in the references and in the "Availability of Data and Materials" section of your manuscript.<br><br>Have you have met the above requirement as detailed in our Minimum Standards Reporting Checklist?	Yes
GigaScience has policies and guidelines in place for the use of generative AI-	No

writing tools such as ChatGPT. If you have used such writing tools to assist with writing the manuscript this must be declared and cited in the text. Authors should not list AI-writing tools and other AI-assisted technologies as an author or co-author and should acknowledge that they are fully responsible for text generated or refined by AI-writing tools.<p><br><br>A summary of use (particularly in the introduction or among methods) needs to be included at the end of the paper, and the outputs should also be included as a supplementary file hosted in GigaDB or other open repositories. Please read our guidelines for more information. <p><br><br>By submitting to GigaScience, you are aware of the journal's AI-writing tools policy, and if you have declared use of such tools below, you have acknowledged this where appropriate in your manuscript and have made a summary of use and outputs available. <p><br><b>AI-assisted writing tools have been used in the preparation of this manuscript?	

LaTeX - Main Document (TeX file)
Click here to access/download;LaTeX - Main Document (TeX file);main.tex

```
This is pdfTeX, Version 3.141592653-2.6-1.40.26 (TeX Live 2024)
(preloaded format=pdflatex 2024.8.2)  20 JUN 2025 07:09
entering extended mode
 restricted \write18 enabled.
 %&-line parsing enabled.
**main.tex
(./main.tex
LaTeX2e <2024-06-01> patch level 2
L3 programming layer <2024-05-27>
(./oup-contemporary.cls
Document Class: oup-contemporary 2023/06/12, v1.2
(c:/texlive/2024/texmf-dist/tex/latex/base/article.cls
Document Class: article 2024/02/08 v1.4n Standard LaTeX document class
(c:/texlive/2024/texmf-dist/tex/latex/base/size10.clo
File: size10.clo 2024/02/08 v1.4n Standard LaTeX file (size option)
)
\c@part=\count194
\c@section=\count195
\c@subsection=\count196
\c@subsubsection=\count197
\c@paragraph=\count198
\c@subparagraph=\count199
\c@figure=\count266
\c@table=\count267
\abovecaptionskip=\skip49
\belowcaptionskip=\skip50
\bibindent=\dimen141
) (c:/texlive/2024/texmf-dist/tex/latex/base/inputenc.sty
Package: inputenc 2024/02/08 v1.3d Input encoding file
\inpenc@prehook=\toks17
\inpenc@posthook=\toks18
) (c:/texlive/2024/texmf-dist/tex/latex/base/fontenc.sty
Package: fontenc 2021/04/29 v2.0v Standard LaTeX package
) (c:/texlive/2024/texmf-dist/tex/generic/iftex/ifpdf.sty
Package: ifpdf 2019/10/25 v3.4 ifpdf legacy package. Use iftex instead.
(c:/texlive/2024/texmf-dist/tex/generic/iftex/iftex.sty
Package: iftex 2022/02/03 v1.0f TeX engine tests
)) (c:/texlive/2024/texmf-dist/tex/latex/microtype/microtype.sty
Package: microtype 2024/03/29 v3.1b Micro-typographical refinements (RS)
(c:/texlive/2024/texmf-dist/tex/latex/graphics/keyval.sty
Package: keyval 2022/05/29 v1.15 key=value parser (DPC)
\KV@toks@=\toks19
) (c:/texlive/2024/texmf-dist/tex/latex/etoolbox/etoolbox.sty
Package: etoolbox 2020/10/05 v2.5k e-TeX tools for LaTeX (JAW)
\etb@tempcnta=\count268
)
\MT@toks=\toks20
\MT@tempbox=\box52
\MT@count=\count269
LaTeX Info: Redefining \noprotrusionifhmode on input line 1061.
LaTeX Info: Redefining \leftprotrusion on input line 1062.
\MT@prot@toks=\toks21
LaTeX Info: Redefining \rightprotrusion on input line 1081.
LaTeX Info: Redefining \textls on input line 1392.
```

```
\MT@outer@kern=\dimen142
LaTeX Info: Redefining \textmicrotypecontext on input line 2013.
\MT@listname@count=\count270
(c:/texlive/2024/texmf-dist/tex/latex/microtype/microtype-pdftex.def
File: microtype-pdftex.def 2024/03/29 v3.1b Definitions specific to
pdftex (RS)

LaTeX Info: Redefining \lsstyle on input line 902.
LaTeX Info: Redefining \lslig on input line 902.
\MT@outer@space=\skip51
)
Package microtype Info: Loading configuration file microtype.cfg.
(c:/texlive/2024/texmf-dist/tex/latex/microtype/microtype.cfg
File: microtype.cfg 2024/03/29 v3.1b microtype main configuration file
(RS)
)) (c:/texlive/2024/texmf-dist/tex/latex/euler/euler.sty
Package: euler 1995/03/05 v2.5
Package: `euler' v2.5 <1995/03/05> (FJ and FMi)
LaTeX Font Info:    Redeclaring symbol font `letters' on input line 35.
LaTeX Font Info:    Encoding `OML' has changed to `U' for symbol font
(Font)              `letters' in the math version `normal' on input line
35.
LaTeX Font Info:    Overwriting symbol font `letters' in version `normal'
(Font)                  OML/cmm/m/it --> U/eur/m/n on input line 35.
LaTeX Font Info:    Encoding `OML' has changed to `U' for symbol font
(Font)              `letters' in the math version `bold' on input line
35.
LaTeX Font Info:    Overwriting symbol font `letters' in version `bold'
(Font)                  OML/cmm/b/it --> U/eur/m/n on input line 35.
LaTeX Font Info:    Overwriting symbol font `letters' in version `bold'
(Font)                  U/eur/m/n --> U/eur/b/n on input line 36.
LaTeX Font Info:    Redeclaring math symbol \Gamma on input line 47.
LaTeX Font Info:    Redeclaring math symbol \Delta on input line 48.
LaTeX Font Info:    Redeclaring math symbol \Theta on input line 49.
LaTeX Font Info:    Redeclaring math symbol \Lambda on input line 50.
LaTeX Font Info:    Redeclaring math symbol \Xi on input line 51.
LaTeX Font Info:    Redeclaring math symbol \Pi on input line 52.
LaTeX Font Info:    Redeclaring math symbol \Sigma on input line 53.
LaTeX Font Info:    Redeclaring math symbol \Upsilon on input line 54.
LaTeX Font Info:    Redeclaring math symbol \Phi on input line 55.
LaTeX Font Info:    Redeclaring math symbol \Psi on input line 56.
LaTeX Font Info:    Redeclaring math symbol \Omega on input line 57.
\symEulerFraktur=\mathgroup4
LaTeX Font Info:    Overwriting symbol font `EulerFraktur' in version
`bold'
(Font)                  U/euf/m/n --> U/euf/b/n on input line 63.
LaTeX Info: Redefining \oldstylenums on input line 85.
\symEulerScript=\mathgroup5
LaTeX Font Info:    Overwriting symbol font `EulerScript' in version
`bold'
(Font)                  U/eus/m/n --> U/eus/b/n on input line 93.
LaTeX Font Info:    Redeclaring math symbol \aleph on input line 97.
LaTeX Font Info:    Redeclaring math symbol \Re on input line 98.
LaTeX Font Info:    Redeclaring math symbol \Im on input line 99.
```

```
LaTeX Font Info:    Redeclaring math delimiter \vert on input line 101.
LaTeX Font Info:    Redeclaring math delimiter \backslash on input line
103.
LaTeX Font Info:    Redeclaring math symbol \neg on input line 106.
LaTeX Font Info:    Redeclaring math symbol \wedge on input line 108.
LaTeX Font Info:    Redeclaring math symbol \vee on input line 110.
LaTeX Font Info:    Redeclaring math symbol \setminus on input line 112.
LaTeX Font Info:    Redeclaring math symbol \sim on input line 113.
LaTeX Font Info:    Redeclaring math symbol \mid on input line 114.
LaTeX Font Info:    Redeclaring math delimiter \arrowvert on input line
116.
LaTeX Font Info:    Redeclaring math symbol \mathsection on input line
117.
\symEulerExtension=\mathgroup6
LaTeX Font Info:    Redeclaring math symbol \coprod on input line 125.
LaTeX Font Info:    Redeclaring math symbol \prod on input line 125.
LaTeX Font Info:    Redeclaring math symbol \sum on input line 125.
LaTeX Font Info:    Redeclaring math symbol \intop on input line 130.
LaTeX Font Info:    Redeclaring math symbol \ointop on input line 131.
LaTeX Font Info:    Redeclaring math symbol \braceld on input line 132.
LaTeX Font Info:    Redeclaring math symbol \bracerd on input line 133.
LaTeX Font Info:    Redeclaring math symbol \bracelu on input line 134.
LaTeX Font Info:    Redeclaring math symbol \braceru on input line 135.
LaTeX Font Info:    Redeclaring math symbol \infty on input line 136.
LaTeX Font Info:    Redeclaring math symbol \nearrow on input line 153.
LaTeX Font Info:    Redeclaring math symbol \searrow on input line 154.
LaTeX Font Info:    Redeclaring math symbol \nwarrow on input line 155.
LaTeX Font Info:    Redeclaring math symbol \swarrow on input line 156.
LaTeX Font Info:    Redeclaring math symbol \Leftrightarrow on input line
157.
LaTeX Font Info:    Redeclaring math symbol \Leftarrow on input line 158.
LaTeX Font Info:    Redeclaring math symbol \Rightarrow on input line
159.
LaTeX Font Info:    Redeclaring math symbol \leftrightarrow on input line
160.
LaTeX Font Info:    Redeclaring math symbol \leftarrow on input line 161.
LaTeX Font Info:    Redeclaring math symbol \rightarrow on input line
163.
LaTeX Font Info:    Redeclaring math delimiter \uparrow on input line
166.
LaTeX Font Info:    Redeclaring math delimiter \downarrow on input line
168.
LaTeX Font Info:    Redeclaring math delimiter \updownarrow on input line
170.
LaTeX Font Info:    Redeclaring math delimiter \Uparrow on input line
172.
LaTeX Font Info:    Redeclaring math delimiter \Downarrow on input line
174.
LaTeX Font Info:    Redeclaring math delimiter \Updownarrow on input line
176.
LaTeX Font Info:    Redeclaring math symbol \leftharpoonup on input line
177.
LaTeX Font Info:    Redeclaring math symbol \leftharpoondown on input
line 178.
```

```
LaTeX Font Info:    Redeclaring math symbol \rightharpoonup on input line
179.
LaTeX Font Info:    Redeclaring math symbol \rightharpoondown on input
line 180
.
LaTeX Font Info:    Redeclaring math delimiter \lbrace on input line 182.
LaTeX Font Info:    Redeclaring math delimiter \rbrace on input line 184.
\symcmmigroup=\mathgroup7
LaTeX Font Info:    Overwriting symbol font `cmmigroup' in version `bold'
(Font)                  OML/cmm/m/it --> OML/cmm/b/it on input line 200.
LaTeX Font Info:    Redeclaring math accent \vec on input line 201.
LaTeX Font Info:    Redeclaring math symbol \triangleleft on input line
202.
LaTeX Font Info:    Redeclaring math symbol \triangleright on input line
203.
LaTeX Font Info:    Redeclaring math symbol \star on input line 204.
LaTeX Font Info:    Redeclaring math symbol \lhook on input line 205.
LaTeX Font Info:    Redeclaring math symbol \rhook on input line 206.
LaTeX Font Info:    Redeclaring math symbol \flat on input line 207.
LaTeX Font Info:    Redeclaring math symbol \natural on input line 208.
LaTeX Font Info:    Redeclaring math symbol \sharp on input line 209.
LaTeX Font Info:    Redeclaring math symbol \smile on input line 210.
LaTeX Font Info:    Redeclaring math symbol \frown on input line 211.
LaTeX Font Info:    Redeclaring math accent \grave on input line 245.
LaTeX Font Info:    Redeclaring math accent \acute on input line 246.
LaTeX Font Info:    Redeclaring math accent \tilde on input line 247.
LaTeX Font Info:    Redeclaring math accent \ddot on input line 248.
LaTeX Font Info:    Redeclaring math accent \check on input line 249.
LaTeX Font Info:    Redeclaring math accent \breve on input line 250.
LaTeX Font Info:    Redeclaring math accent \bar on input line 251.
LaTeX Font Info:    Redeclaring math accent \dot on input line 252.
LaTeX Font Info:    Redeclaring math accent \hat on input line 254.
) (c:/texlive/2024/texmf-dist/tex/latex/merriweather/merriweather.sty
Package: merriweather 2022/09/20 (Bob Tennent) Supports
Merriweather(Sans) font
s for all LaTeX engines.
(c:/texlive/2024/texmf-dist/tex/generic/iftex/ifxetex.sty
Package: ifxetex 2019/10/25 v0.7 ifxetex legacy package. Use iftex
instead.
) (c:/texlive/2024/texmf-dist/tex/generic/iftex/ifluatex.sty
Package: ifluatex 2019/10/25 v1.5 ifluatex legacy package. Use iftex
instead.
) (c:/texlive/2024/texmf-dist/tex/latex/base/textcomp.sty
Package: textcomp 2024/04/24 v2.1b Standard LaTeX package
) (c:/texlive/2024/texmf-dist/tex/latex/xkeyval/xkeyval.sty
Package: xkeyval 2022/06/16 v2.9 package option processing (HA)
(c:/texlive/2024/texmf-dist/tex/generic/xkeyval/xkeyval.tex
(c:/texlive/2024/te
xmf-dist/tex/generic/xkeyval/xkvutils.tex
\XKV@toks=\toks22
\XKV@tempa@toks=\toks23
)
\XKV@depth=\count271
```

```
File: xkeyval.tex 2014/12/03 v2.7a key=value parser (HA)
)) (c:/texlive/2024/texmf-dist/tex/latex/base/fontenc.sty
Package: fontenc 2021/04/29 v2.0v Standard LaTeX package
) (c:/texlive/2024/texmf-dist/tex/latex/fontaxes/fontaxes.sty
Package: fontaxes 2020/07/21 v1.0e Font selection axes
LaTeX Info: Redefining \upshape on input line 29.
LaTeX Info: Redefining \itshape on input line 31.
LaTeX Info: Redefining \slshape on input line 33.
LaTeX Info: Redefining \swshape on input line 35.
LaTeX Info: Redefining \scshape on input line 37.
LaTeX Info: Redefining \sscshape on input line 39.
LaTeX Info: Redefining \ulcshape on input line 41.
LaTeX Info: Redefining \textsw on input line 47.
LaTeX Info: Redefining \textssc on input line 48.
LaTeX Info: Redefining \textulc on input line 49.
)) (c:/texlive/2024/texmf-dist/tex/latex/mathastext/mathastext.sty
Package: mathastext 2024/07/27 v1.4b Use the text font in math mode (JFB)

Package mathastext Info: Starting the math mode configuration.
\mst@exists@muskip=\muskip17
\mst@forall@muskip=\muskip18
\mst@prime@muskip=\muskip19
\mst@do@nonletters=\toks24
\mst@undo@nonletters=\toks25
\mst@do@easynonletters=\toks26
\mst@undo@easynonletters=\toks27
\symmtoperatorfont=\mathgroup8
\symmtletterfont=\mathgroup9
(   mathastext:   ) ! and ?
(   mathastext:   ) punctuation: , . : ; and \colon
LaTeX Info: Redefining \relbar on input line 1201.
LaTeX Info: Redefining \rightarrowfill on input line 1202.
LaTeX Info: Redefining \leftarrowfill on input line 1205.
(   mathastext:   ) + and =
LaTeX Info: Redefining \Relbar on input line 1298.
(   mathastext:   ) adding = ; and + to \nfss@catcodes
(   mathastext:   ) parentheses ( ) [ ] and slash /
(   mathastext:   ) alldelims: < > \backslash \setminus | \vert \mid \{
\}
LaTeX Font Info:    Redeclaring math symbol \setminus on input line 1364.
LaTeX Info: Redefining \models on input line 1383.
(   mathastext:   ) \# \mathdollar \% \&
(   mathastext:   ) \imath and \jmath
LaTeX Font Info:    Overwriting math alphabet `\Mathnormalbold' in
version `nor
mal'
(Font)                  T1/Merriwthr-OsF/b/it --> T1/Merriwthr-OsF/b/it
on inpu
t line 2863.
LaTeX Font Info:    Overwriting math alphabet `\Mathnormalbold' in
version `bol
d'
(Font)                  T1/Merriwthr-OsF/b/it --> T1/Merriwthr-OsF/b/it
on inpu
```

```
t line 2863.
LaTeX Font Info:    Overwriting symbol font `mtletterfont' in version
`normal'
(Font)                  T1/Merriwthr-OsF/m/it --> T1/Merriwthr-OsF/m/it
on inpu
t line 2863.
LaTeX Font Info:    Overwriting symbol font `mtletterfont' in version
`bold'
(Font)                  T1/Merriwthr-OsF/m/it --> T1/Merriwthr-OsF/b/it
on inpu
t line 2863.
LaTeX Font Info:    Overwriting symbol font `mtoperatorfont' in version
`normal
'
(Font)                  T1/Merriwthr-OsF/m/n --> T1/Merriwthr-OsF/m/n on
input
line 2863.
LaTeX Font Info:    Overwriting symbol font `mtoperatorfont' in version
`bold'
(Font)                  T1/Merriwthr-OsF/m/n --> T1/Merriwthr-OsF/b/n on
input
line 2863.
LaTeX Font Info:    Overwriting math alphabet `\Mathbf' in version
`normal'
(Font)                  T1/Merriwthr-OsF/b/n --> T1/Merriwthr-OsF/b/n on
input
line 2863.
LaTeX Font Info:    Overwriting math alphabet `\Mathbf' in version `bold'
(Font)                  T1/Merriwthr-OsF/b/n --> T1/Merriwthr-OsF/b/n on
input
line 2863.
LaTeX Font Info:    Overwriting math alphabet `\Mathit' in version
`normal'
(Font)                  T1/Merriwthr-OsF/m/it --> T1/Merriwthr-OsF/m/it
on inpu
t line 2863.
LaTeX Font Info:    Overwriting math alphabet `\Mathit' in version `bold'
(Font)                  T1/Merriwthr-OsF/m/it --> T1/Merriwthr-OsF/b/it
on inpu
t line 2863.
LaTeX Font Info:    Overwriting math alphabet `\Mathsf' in version
`normal'
(Font)                  T1/MerriwthrSans-OsF/m/n --> T1/MerriwthrSans-
OsF/m/n o
n input line 2863.
LaTeX Font Info:    Overwriting math alphabet `\Mathsf' in version `bold'
(Font)                  T1/MerriwthrSans-OsF/m/n --> T1/MerriwthrSans-
OsF/b/n o
n input line 2863.
LaTeX Font Info:    Overwriting math alphabet `\Mathtt' in version
`normal'
(Font)                  T1/lmtt/m/n --> T1/lmtt/m/n on input line 2863.
LaTeX Font Info:    Overwriting math alphabet `\Mathtt' in version `bold'
(Font)                  T1/lmtt/m/n --> T1/lmtt/b/n on input line 2863.
```

```
(   mathastext:   ) Latin letters in the `normal', resp. `bold',
(   mathastext:   ) math versions are now set up to use the fonts
(   mathastext:   ) T1/Merriwthr-OsF/m/it, resp. T1/Merriwthr-OsF/b/it.
(   mathastext:   ) Other characters (digits, ...) and \log-like names
will be
(   mathastext:   ) typeset with the n shape.
(   mathastext:   ) \hbar
(   mathastext:   ) minus as endash
(   mathastext:   ) The italic option is in effect.
(   mathastext:   ) \HUGE has been (re)-defined.
(   mathastext:   ) mathastext has declared larger sizes for subscripts.
(   mathastext:   ) To keep LaTeX defaults, use option
`defaultmathsizes'.

Package mathastext Info: Loading is complete.  You can now use
\Mathastext to
(mathastext)             modify the normal and bold math versions.  Use
it
(mathastext)             with optional argument or use \MTDeclareVersion
to
(mathastext)             declare additional math versions.
) (c:/texlive/2024/texmf-dist/tex/latex/relsize/relsize.sty
Package: relsize 2013/03/29 ver 4.1
) (c:/texlive/2024/texmf-dist/tex/latex/ragged2e/ragged2e.sty
Package: ragged2e 2023/06/22 v3.6 ragged2e Package
\CenteringLeftskip=\skip52
\RaggedLeftLeftskip=\skip53
\RaggedRightLeftskip=\skip54
\CenteringRightskip=\skip55
\RaggedLeftRightskip=\skip56
\RaggedRightRightskip=\skip57
\CenteringParfillskip=\skip58
\RaggedLeftParfillskip=\skip59
\RaggedRightParfillskip=\skip60
\JustifyingParfillskip=\skip61
\CenteringParindent=\skip62
\RaggedLeftParindent=\skip63
\RaggedRightParindent=\skip64
\JustifyingParindent=\skip65
) (c:/texlive/2024/texmf-dist/tex/latex/xcolor/xcolor.sty
Package: xcolor 2023/11/15 v3.01 LaTeX color extensions (UK)
(c:/texlive/2024/texmf-dist/tex/latex/graphics-cfg/color.cfg
File: color.cfg 2016/01/02 v1.6 sample color configuration
)
Package xcolor Info: Driver file: pdftex.def on input line 274.
(c:/texlive/2024/texmf-dist/tex/latex/graphics-def/pdftex.def
File: pdftex.def 2024/04/13 v1.2c Graphics/color driver for pdftex
) (c:/texlive/2024/texmf-dist/tex/latex/graphics/mathcolor.ltx)
Package xcolor Info: Model `cmy' substituted by `cmy0' on input line
1350.
Package xcolor Info: Model `hsb' substituted by `rgb' on input line 1354.
Package xcolor Info: Model `RGB' extended on input line 1366.
Package xcolor Info: Model `HTML' substituted by `rgb' on input line
1368.
```

```
Package xcolor Info: Model `Hsb' substituted by `hsb' on input line 1369.
Package xcolor Info: Model `tHsb' substituted by `hsb' on input line
1370.
Package xcolor Info: Model `HSB' substituted by `hsb' on input line 1371.
Package xcolor Info: Model `Gray' substituted by `gray' on input line
1372.
Package xcolor Info: Model `wave' substituted by `hsb' on input line
1373.
) (c:/texlive/2024/texmf-dist/tex/latex/colortbl/colortbl.sty
Package: colortbl 2024/07/06 v1.0i Color table columns (DPC)
(c:/texlive/2024/texmf-dist/tex/latex/tools/array.sty
Package: array 2024/06/14 v2.6d Tabular extension package (FMi)
\col@sep=\dimen143
\ar@mcellbox=\box53
\extrarowheight=\dimen144
\NC@list=\toks28
\extratabsurround=\skip66
\backup@length=\skip67
\ar@cellbox=\box54
)
\everycr=\toks29
\minrowclearance=\skip68
\rownum=\count272
) (c:/texlive/2024/texmf-dist/tex/latex/graphics/graphicx.sty
Package: graphicx 2021/09/16 v1.2d Enhanced LaTeX Graphics (DPC,SPQR)
(c:/texlive/2024/texmf-dist/tex/latex/graphics/graphics.sty
Package: graphics 2024/05/23 v1.4g Standard LaTeX Graphics (DPC,SPQR)
(c:/texlive/2024/texmf-dist/tex/latex/graphics/trig.sty
Package: trig 2023/12/02 v1.11 sin cos tan (DPC)
) (c:/texlive/2024/texmf-dist/tex/latex/graphics-cfg/graphics.cfg
File: graphics.cfg 2016/06/04 v1.11 sample graphics configuration
)
Package graphics Info: Driver file: pdftex.def on input line 106.
)
\Gin@req@height=\dimen145
\Gin@req@width=\dimen146
) (c:/texlive/2024/texmf-dist/tex/latex/xpatch/xpatch.sty
(c:/texlive/2024/texm
f-dist/tex/latex/l3kernel/expl3.sty
Package: expl3 2024-05-27 L3 programming layer (loader)
(c:/texlive/2024/texmf-dist/tex/latex/l3backend/l3backend-pdftex.def
File: l3backend-pdftex.def 2024-05-08 L3 backend support: PDF output
(pdfTeX)
\l__color_backend_stack_int=\count273
\l__pdf_internal_box=\box55
))
Package: xpatch 2020/03/25 v0.3a Extending etoolbox patching commands
(c:/texlive/2024/texmf-dist/tex/latex/l3packages/xparse/xparse.sty
Package: xparse 2024-05-08 L3 Experimental document command parser
)) (c:/texlive/2024/texmf-dist/tex/latex/environ/environ.sty
Package: environ 2014/05/04 v0.3 A new way to define environments
(c:/texlive/2024/texmf-dist/tex/latex/trimspaces/trimspaces.sty
Package: trimspaces 2009/09/17 v1.1 Trim spaces around a token list
)
```

```
\@envbody=\toks30
) (c:/texlive/2024/texmf-dist/tex/latex/lastpage/lastpage.sty
Package: lastpage 2024/07/07 v2.1c lastpage: 2.09 or 2e? (HMM)
(c:/texlive/2024/texmf-dist/tex/latex/lastpage/lastpage2e.sty
Package: lastpage2e 2024/07/07 v2.1c Decide which 2e lastpage version to
use (H
MM)
(c:/texlive/2024/texmf-dist/tex/latex/lastpage/lastpagemodern.sty
Package: lastpagemodern 2024-07-07 v2.1c Refers to last page's name (HMM;
JPG)
\c@lastpagecount=\count274
)
)) (c:/texlive/2024/texmf-dist/tex/latex/graphics/rotating.sty
Package: rotating 2016/08/11 v2.16d rotated objects in LaTeX
(c:/texlive/2024/texmf-dist/tex/latex/base/ifthen.sty
Package: ifthen 2024/03/16 v1.1e Standard LaTeX ifthen package (DPC)
)
\c@r@tfl@t=\count275
\rotFPtop=\skip69
\rotFPbot=\skip70
\rot@float@box=\box56
\rot@mess@toks=\toks31
) (c:/texlive/2024/texmf-dist/tex/latex/graphics/lscape.sty
Package: lscape 2020/05/28 v3.02 Landscape Pages (DPC)
) (c:/texlive/2024/texmf-dist/tex/latex/tools/afterpage.sty
Package: afterpage 2023/07/04 v1.08 After-Page Package (DPC)
\AP@output=\toks32
\AP@partial=\box57
\AP@footins=\box58
) (c:/texlive/2024/texmf-dist/tex/latex/textpos/textpos.sty
Package: textpos 2022/07/23 v1.10.1
Package textpos Info: choosing support for LaTeX3 on input line 60.
\TP@textbox=\box59
\TP@holdbox=\box60
\TPHorizModule=\dimen147
\TPVertModule=\dimen148
\TP@margin=\dimen149
\TP@absmargin=\dimen150
Grid set 16 x 16 = 37.34424pt x 52.81541pt
\TPboxrulesize=\dimen151
\TP@ox=\dimen152
\TP@oy=\dimen153
\TP@tbargs=\toks33
TextBlockOrigin set to 0pt x 0pt
) (c:/texlive/2024/texmf-dist/tex/latex/url/url.sty
\Urlmuskip=\muskip20
Package: url 2013/09/16  ver 3.4  Verb mode for urls, etc.
) (c:/texlive/2024/texmf-dist/tex/latex/newfloat/newfloat.sty
Package: newfloat 2023/10/01 v1.2 Defining new floating environments (AR)
Package newfloat Info: `rotating' package detected.
) (c:/texlive/2024/texmf-dist/tex/latex/mdframed/mdframed.sty
Package: mdframed 2013/07/01 1.9b: mdframed
(c:/texlive/2024/texmf-dist/tex/latex/kvoptions/kvoptions.sty
```

```
Package: kvoptions 2022-06-15 v3.15 Key value format for package options
(HO)
(c:/texlive/2024/texmf-dist/tex/generic/ltxcmds/ltxcmds.sty
Package: ltxcmds 2023-12-04 v1.26 LaTeX kernel commands for general use
(HO)
) (c:/texlive/2024/texmf-dist/tex/latex/kvsetkeys/kvsetkeys.sty
Package: kvsetkeys 2022-10-05 v1.19 Key value parser (HO)
)) (c:/texlive/2024/texmf-dist/tex/latex/zref/zref-abspage.sty
Package: zref-abspage 2023-09-14 v2.35 Module abspage for zref (HO)
(c:/texlive/2024/texmf-dist/tex/latex/zref/zref-base.sty
Package: zref-base 2023-09-14 v2.35 Module base for zref (HO)
(c:/texlive/2024/texmf-dist/tex/generic/infwarerr/infwarerr.sty
Package: infwarerr 2019/12/03 v1.5 Providing info/warning/error messages
(HO)
) (c:/texlive/2024/texmf-dist/tex/generic/kvdefinekeys/kvdefinekeys.sty
Package: kvdefinekeys 2019-12-19 v1.6 Define keys (HO)
) (c:/texlive/2024/texmf-dist/tex/generic/pdftexcmds/pdftexcmds.sty
Package: pdftexcmds 2020-06-27 v0.33 Utility functions of pdfTeX for
LuaTeX (HO
)
Package pdftexcmds Info: \pdf@primitive is available.
Package pdftexcmds Info: \pdf@ifprimitive is available.
Package pdftexcmds Info: \pdfdraftmode found.
) (c:/texlive/2024/texmf-dist/tex/generic/etexcmds/etexcmds.sty
Package: etexcmds 2019/12/15 v1.7 Avoid name clashes with e-TeX commands
(HO)
) (c:/texlive/2024/texmf-dist/tex/latex/auxhook/auxhook.sty
Package: auxhook 2019-12-17 v1.6 Hooks for auxiliary files (HO)
)
Package zref Info: New property list: main on input line 767.
Package zref Info: New property: default on input line 768.
Package zref Info: New property: page on input line 769.
)
\c@abspage=\count276
Package zref Info: New property: abspage on input line 67.
) (c:/texlive/2024/texmf-dist/tex/latex/needspace/needspace.sty
Package: needspace 2010/09/12 v1.3d reserve vertical space
)
\mdf@templength=\skip71
\c@mdf@globalstyle@cnt=\count277
\mdf@skipabove@length=\skip72
\mdf@skipbelow@length=\skip73
\mdf@leftmargin@length=\skip74
\mdf@rightmargin@length=\skip75
\mdf@innerleftmargin@length=\skip76
\mdf@innerrightmargin@length=\skip77
\mdf@innertopmargin@length=\skip78
\mdf@innerbottommargin@length=\skip79
\mdf@splittopskip@length=\skip80
\mdf@splitbottomskip@length=\skip81
\mdf@outermargin@length=\skip82
\mdf@innermargin@length=\skip83
\mdf@linewidth@length=\skip84
\mdf@innerlinewidth@length=\skip85
```

```
\mdf@middlelinewidth@length=\skip86
\mdf@outerlinewidth@length=\skip87
\mdf@roundcorner@length=\skip88
\mdf@footenotedistance@length=\skip89
\mdf@userdefinedwidth@length=\skip90
\mdf@needspace@length=\skip91
\mdf@frametitleaboveskip@length=\skip92
\mdf@frametitlebelowskip@length=\skip93
\mdf@frametitlerulewidth@length=\skip94
\mdf@frametitleleftmargin@length=\skip95
\mdf@frametitlerightmargin@length=\skip96
\mdf@shadowsize@length=\skip97
\mdf@extratopheight@length=\skip98
\mdf@subtitleabovelinewidth@length=\skip99
\mdf@subtitlebelowlinewidth@length=\skip100
\mdf@subtitleaboveskip@length=\skip101
\mdf@subtitlebelowskip@length=\skip102
\mdf@subtitleinneraboveskip@length=\skip103
\mdf@subtitleinnerbelowskip@length=\skip104
\mdf@subsubtitleabovelinewidth@length=\skip105
\mdf@subsubtitlebelowlinewidth@length=\skip106
\mdf@subsubtitleaboveskip@length=\skip107
\mdf@subsubtitlebelowskip@length=\skip108
\mdf@subsubtitleinneraboveskip@length=\skip109
\mdf@subsubtitleinnerbelowskip@length=\skip110
(c:/texlive/2024/texmf-dist/tex/latex/mdframed/md-frame-0.mdf
File: md-frame-0.mdf 2013/07/01\ 1.9b: md-frame-0
)
\mdf@frametitlebox=\box61
\mdf@footnotebox=\box62
\mdf@splitbox@one=\box63
\mdf@splitbox@two=\box64
\mdf@splitbox@save=\box65
\mdfsplitboxwidth=\skip111
\mdfsplitboxtotalwidth=\skip112
\mdfsplitboxheight=\skip113
\mdfsplitboxdepth=\skip114
\mdfsplitboxtotalheight=\skip115
\mdfframetitleboxwidth=\skip116
\mdfframetitleboxtotalwidth=\skip117
\mdfframetitleboxheight=\skip118
\mdfframetitleboxdepth=\skip119
\mdfframetitleboxtotalheight=\skip120
\mdffootnoteboxwidth=\skip121
\mdffootnoteboxtotalwidth=\skip122
\mdffootnoteboxheight=\skip123
\mdffootnoteboxdepth=\skip124
\mdffootnoteboxtotalheight=\skip125
\mdftotallinewidth=\skip126
\mdfboundingboxwidth=\skip127
\mdfboundingboxtotalwidth=\skip128
\mdfboundingboxheight=\skip129
\mdfboundingboxdepth=\skip130
\mdfboundingboxtotalheight=\skip131
```

```
\mdf@freevspace@length=\skip132
\mdf@horizontalwidthofbox@length=\skip133
\mdf@verticalmarginwhole@length=\skip134
\mdf@horizontalspaceofbox=\skip135
\mdfsubtitleheight=\skip136
\mdfsubsubtitleheight=\skip137
\c@mdfcountframes=\count278

****** mdframed patching \endmdf@trivlist

****** -- success******

\mdf@envdepth=\count279
\c@mdf@env@i=\count280
\c@mdf@env@ii=\count281
\c@mdf@zref@counter=\count282
Package zref Info: New property: mdf@pagevalue on input line 895.
) (c:/texlive/2024/texmf-dist/tex/latex/titlesec/titlesec.sty
Package: titlesec 2023/10/27 v2.16 Sectioning titles
\ttl@box=\box66
\beforetitleunit=\skip138
\aftertitleunit=\skip139
\ttl@plus=\dimen154
\ttl@minus=\dimen155
\ttl@toksa=\toks34
\titlewidth=\dimen156
\titlewidthlast=\dimen157
\titlewidthfirst=\dimen158
) (c:/texlive/2024/texmf-dist/tex/latex/koma-script/scrextend.sty
Package: scrextend 2023/07/07 v3.41 KOMA-Script package (extend other
classes w
ith features of KOMA-Script classes)
(c:/texlive/2024/texmf-dist/tex/latex/koma-script/scrkbase.sty
Package: scrkbase 2023/07/07 v3.41 KOMA-Script package (KOMA-Script-
dependent b
asics and keyval usage)
(c:/texlive/2024/texmf-dist/tex/latex/koma-script/scrbase.sty
Package: scrbase 2023/07/07 v3.41 KOMA-Script package (KOMA-Script-
independent
basics and keyval usage)
(c:/texlive/2024/texmf-dist/tex/latex/koma-script/scrlfile.sty
Package: scrlfile 2023/07/07 v3.41 KOMA-Script package (file load hooks)
(c:/texlive/2024/texmf-dist/tex/latex/koma-script/scrlfile-hook.sty
Package: scrlfile-hook 2023/07/07 v3.41 KOMA-Script package (using LaTeX
hooks)

(c:/texlive/2024/texmf-dist/tex/latex/koma-script/scrlogo.sty
Package: scrlogo 2023/07/07 v3.41 KOMA-Script package (logo)
)))
Applying: [2021/05/01] Usage of raw or classic option list on input line
252.
Already applied: [0000/00/00] Usage of raw or classic option list on
input line
 368.
```

```
))
Package scrextend Info: unexpected definition of `\@makefnmark'.
(scrextend)             Trying to patch it on input line 1762.
Package scrextend Info: patch seems to be successfull on input line 1762.
)

LaTeX Font Warning: Font shape `T1/cmr/m/n' in size <7.5> not available
(Font)              size <7> substituted on input line 69.

(c:/texlive/2024/texmf-dist/tex/latex/tools/calc.sty
Package: calc 2023/07/08 v4.3 Infix arithmetic (KKT,FJ)
\calc@Acount=\count283
\calc@Bcount=\count284
\calc@Adimen=\dimen159
\calc@Bdimen=\dimen160
\calc@Askip=\skip140
\calc@Bskip=\skip141
LaTeX Info: Redefining \setlength on input line 80.
LaTeX Info: Redefining \addtolength on input line 81.
\calc@Ccount=\count285
\calc@Cskip=\skip142
) (c:/texlive/2024/texmf-dist/tex/latex/geometry/geometry.sty
Package: geometry 2020/01/02 v5.9 Page Geometry
(c:/texlive/2024/texmf-dist/tex/generic/iftex/ifvtex.sty
Package: ifvtex 2019/10/25 v1.7 ifvtex legacy package. Use iftex instead.
)
\Gm@cnth=\count286
\Gm@cntv=\count287
\c@Gm@tempcnt=\count288
\Gm@bindingoffset=\dimen161
\Gm@wd@mp=\dimen162
\Gm@odd@mp=\dimen163
\Gm@even@mp=\dimen164
\Gm@layoutwidth=\dimen165
\Gm@layoutheight=\dimen166
\Gm@layouthoffset=\dimen167
\Gm@layoutvoffset=\dimen168
\Gm@dimlist=\toks35
) (c:/texlive/2024/texmf-dist/tex/latex/preprint/authblk.sty
Package: authblk 2001/02/27 1.3 (PWD)
\affilsep=\skip143
\@affilsep=\skip144
\c@Maxaffil=\count289
\c@authors=\count290
\c@affil=\count291
) (c:/texlive/2024/texmf-dist/tex/latex/footmisc/footmisc.sty
Package: footmisc 2023/07/05 v6.0f a miscellany of footnote facilities
\FN@temptoken=\toks36
\footnotemargin=\dimen169
\@outputbox@depth=\dimen170
Package footmisc Info: Declaring symbol style bringhurst on input line
696.
Package footmisc Info: Declaring symbol style chicago on input line 704.
Package footmisc Info: Declaring symbol style wiley on input line 713.
```

```
Package footmisc Info: Declaring symbol style lamport-robust on input
line 724.

Package footmisc Info: Declaring symbol style lamport* on input line 744.
Package footmisc Info: Declaring symbol style lamport*-robust on input
line 765
.
) (c:/texlive/2024/texmf-dist/tex/latex/fancyhdr/fancyhdr.sty
Package: fancyhdr 2024/07/23 v4.3.1 Extensive control of page headers and
foote
rs
\f@nch@headwidth=\skip145
\f@nch@O@elh=\skip146
\f@nch@O@erh=\skip147
\f@nch@O@olh=\skip148
\f@nch@O@orh=\skip149
\f@nch@O@elf=\skip150
\f@nch@O@erf=\skip151
\f@nch@O@olf=\skip152
\f@nch@O@orf=\skip153
) (c:/texlive/2024/texmf-dist/tex/generic/alphalph/alphalph.sty
Package: alphalph 2019/12/09 v2.6 Convert numbers to letters (HO)
(c:/texlive/2024/texmf-dist/tex/generic/intcalc/intcalc.sty
Package: intcalc 2019/12/15 v1.3 Expandable calculations with integers
(HO)
))
\c@authorfn=\count292
(c:/texlive/2024/texmf-dist/tex/latex/abstract/abstract.sty
Package: abstract 2009/06/08 v1.2a configurable abstracts
\abstitleskip=\skip154
\absleftindent=\skip155
\absrightindent=\skip156
\absparindent=\skip157
\absparsep=\skip158
)
Package newfloat Info: New float `keypoints' with options
`placement=t!,name=kp
t' on input line 291.
\c@keypoints=\count293
\newfloat@ftype=\count294
Package newfloat Info: float type `keypoints'=8 on input line 291.
(c:/texlive/2024/texmf-dist/tex/latex/enumitem/enumitem.sty
Package: enumitem 2019/06/20 v3.9 Customized lists
\labelindent=\skip159
\enit@outerparindent=\dimen171
\enit@toks=\toks37
\enit@inbox=\box67
\enit@count@id=\count295
\enitdp@description=\count296
) (c:/texlive/2024/texmf-dist/tex/latex/quoting/quoting.sty
Package: quoting 2014/01/28 v0.1c Consolidated environment for displayed
text
\quo@toppartop=\skip160
) (c:/texlive/2024/texmf-dist/tex/latex/sttools/stfloats.sty
```

```
Package: stfloats 2017/03/27 v3.3 Improve float mechanism and
baselineskip sett
ings
\@dblbotnum=\count297
\c@dblbotnumber=\count298
) (c:/texlive/2024/texmf-dist/tex/latex/booktabs/booktabs.sty
Package: booktabs 2020/01/12 v1.61803398 Publication quality tables
\heavyrulewidth=\dimen172
\lightrulewidth=\dimen173
\cmidrulewidth=\dimen174
\belowrulesep=\dimen175
\belowbottomsep=\dimen176
\aboverulesep=\dimen177
\abovetopsep=\dimen178
\cmidrulesep=\dimen179
\cmidrulekern=\dimen180
\defaultaddspace=\dimen181
\@cmidla=\count299
\@cmidlb=\count300
\@aboverulesep=\dimen182
\@belowrulesep=\dimen183
\@thisruleclass=\count301
\@lastruleclass=\count302
\@thisrulewidth=\dimen184
) (c:/texlive/2024/texmf-dist/tex/latex/tools/tabularx.sty
Package: tabularx 2023/12/11 v2.12a `tabularx' package (DPC)
\TX@col@width=\dimen185
\TX@old@table=\dimen186
\TX@old@col=\dimen187
\TX@target=\dimen188
\TX@delta=\dimen189
\TX@cols=\count303
\TX@ftn=\toks38
)
\enitdp@tablenotes=\count304
(c:/texlive/2024/texmf-dist/tex/latex/caption/caption.sty
Package: caption 2023/08/05 v3.6o Customizing captions (AR)
(c:/texlive/2024/texmf-dist/tex/latex/caption/caption3.sty
Package: caption3 2023/07/31 v2.4d caption3 kernel (AR)
\caption@tempdima=\dimen190
\captionmargin=\dimen191
\caption@leftmargin=\dimen192
\caption@rightmargin=\dimen193
\caption@width=\dimen194
\caption@indent=\dimen195
\caption@parindent=\dimen196
\caption@hangindent=\dimen197
Package caption Info: Standard document class detected.
)
\c@caption@flags=\count305
\c@continuedfloat=\count306
Package caption Info: rotating package is loaded.
Package caption Info: scrextend package is loaded.
\caption@addmargin@hsize=\dimen198
```

```
\caption@addmargin@linewidth=\dimen199
) (c:/texlive/2024/texmf-dist/tex/latex/natbib/natbib.sty
Package: natbib 2010/09/13 8.31b (PWD, AO)
\bibhang=\skip161
\bibsep=\skip162
LaTeX Info: Redefining \cite on input line 694.
\c@NAT@ctr=\count307
)) (c:/texlive/2024/texmf-dist/tex/latex/amsfonts/amssymb.sty
Package: amssymb 2013/01/14 v3.01 AMS font symbols
(c:/texlive/2024/texmf-dist/tex/latex/amsfonts/amsfonts.sty
Package: amsfonts 2013/01/14 v3.01 Basic AMSFonts support
\@emptytoks=\toks39
\symAMSa=\mathgroup10
\symAMSb=\mathgroup11
LaTeX Font Info:    Redeclaring math symbol \hbar on input line 98.
LaTeX Info: Redefining \frak on input line 111.
)) (c:/texlive/2024/texmf-dist/tex/latex/psnfss/pifont.sty
Package: pifont 2020/03/25 PSNFSS-v9.3 Pi font support (SPQR)
LaTeX Font Info:    Trying to load font information for U+pzd on input
line 63.

(c:/texlive/2024/texmf-dist/tex/latex/psnfss/upzd.fd
File: upzd.fd 2001/06/04 font definitions for U/pzd.
)
LaTeX Font Info:    Trying to load font information for U+psy on input
line 64.

(c:/texlive/2024/texmf-dist/tex/latex/psnfss/upsy.fd
File: upsy.fd 2001/06/04 font definitions for U/psy.
)) (c:/texlive/2024/texmf-dist/tex/latex/minted/minted.sty
Package: minted 2023/12/18 v2.9 Yet another Pygments shim for LaTeX
(c:/texlive/2024/texmf-dist/tex/latex/fvextra/fvextra.sty
Package: fvextra 2024/05/16 v1.7.0 fvextra - extensions and patches for
fancyvr
b
(c:/texlive/2024/texmf-dist/tex/latex/fancyvrb/fancyvrb.sty
Package: fancyvrb 2024/01/20 4.5c verbatim text (tvz,hv)
\FV@CodeLineNo=\count308
\FV@InFile=\read2
\FV@TabBox=\box68
\c@FancyVerbLine=\count309
\FV@StepNumber=\count310
\FV@OutFile=\write3
) (c:/texlive/2024/texmf-dist/tex/latex/upquote/upquote.sty
Package: upquote 2012/04/19 v1.3 upright-quote and grave-accent glyphs in
verba
tim
) (c:/texlive/2024/texmf-dist/tex/latex/lineno/lineno.sty
Package: lineno 2023/05/20 line numbers on paragraphs v5.3
\linenopenalty=\count311
\output=\toks40
\linenoprevgraf=\count312
\linenumbersep=\dimen256
\linenumberwidth=\dimen257
```

```
\c@linenumber=\count313
\c@pagewiselinenumber=\count314
\c@LN@truepage=\count315
\c@internallinenumber=\count316
\c@internallinenumbers=\count317
\quotelinenumbersep=\dimen258
\bframerule=\dimen259
\bframesep=\dimen260
\bframebox=\box69
LaTeX Info: Redefining \\ on input line 3180.
)
\c@FancyVerbWriteLine=\count318
\c@FancyVerbBufferIndex=\count319
\c@FancyVerbBufferLength=\count320
\c@FancyVerbBufferLine=\count321
\c@FV@oldbufferlength=\count322
\c@FV@TrueTabGroupLevel=\count323
\c@FV@TrueTabCounter=\count324
\FV@TabBox@Group=\box70
\FV@TmpLength=\skip163
\c@FV@HighlightLinesStart=\count325
\c@FV@HighlightLinesStop=\count326
\FV@LoopCount=\count327
\FV@NCharsBox=\box71
\FV@BreakIndent=\dimen261
\FV@BreakIndentNChars=\count328
\FV@BreakSymbolSepLeft=\dimen262
\FV@BreakSymbolSepLeftNChars=\count329
\FV@BreakSymbolSepRight=\dimen263
\FV@BreakSymbolSepRightNChars=\count330
\FV@BreakSymbolIndentLeft=\dimen264
\FV@BreakSymbolIndentLeftNChars=\count331
\FV@BreakSymbolIndentRight=\dimen265
\FV@BreakSymbolIndentRightNChars=\count332
\c@FancyVerbLineBreakLast=\count333
\FV@LineBox=\box72
\FV@LineIndentBox=\box73
\c@FV@BreakBufferDepth=\count334
\FV@LineWidth=\dimen266
) (c:/texlive/2024/texmf-dist/tex/latex/tools/shellesc.sty
Package: shellesc 2023/07/08 v1.0d unified shell escape interface for
LaTeX
Package shellesc Info: Restricted shell escape enabled on input line 77.
) (c:/texlive/2024/texmf-dist/tex/latex/ifplatform/ifplatform.sty
Package: ifplatform 2017/10/13 v0.4a Testing for the operating system
(c:/texlive/2024/texmf-dist/tex/generic/catchfile/catchfile.sty
Package: catchfile 2019/12/09 v1.8 Catch the contents of a file (HO)
)

Package ifplatform Warning:
    shell escape is disabled, so I can only detect \ifwindows.

) (c:/texlive/2024/texmf-dist/tex/generic/xstring/xstring.sty
(c:/texlive/2024/
```

```
texmf-dist/tex/generic/xstring/xstring.tex
\xs_counta=\count335
\xs_countb=\count336
)
Package: xstring 2023/08/22 v1.86 String manipulations (CT)
) (c:/texlive/2024/texmf-dist/tex/latex/framed/framed.sty
Package: framed 2011/10/22 v 0.96: framed or shaded text with page breaks
\OuterFrameSep=\skip164
\fb@frw=\dimen267
\fb@frh=\dimen268
\FrameRule=\dimen269
\FrameSep=\dimen270
) (c:/texlive/2024/texmf-dist/tex/latex/float/float.sty
Package: float 2001/11/08 v1.3d Float enhancements (AL)
\c@float@type=\count337
\float@exts=\toks41
\float@box=\box74
\@float@everytoks=\toks42
\@floatcapt=\box75
)
\minted@appexistsfile=\read3
\minted@bgbox=\box76
\minted@code=\write4
\c@minted@FancyVerbLineTemp=\count338
\c@minted@pygmentizecounter=\count339
\@float@every@listing=\toks43
\c@listing=\count340
)
runsystem(if not exist _minted-main mkdir _minted-main)...disabled
(restricted)
.


! Package minted Error: You must invoke LaTeX with the -shell-escape
flag.

See the minted package documentation for explanation.
Type  H <return>  for immediate help.
 ...

l.27

Pass the -shell-escape flag to LaTeX. Refer to the minted.sty
documentation for
 more information.

(c:/texlive/2024/texmf-dist/tex/latex/siunitx/siunitx.sty
Package: siunitx 2024-06-24 v3.3.19 A comprehensive (SI) units package
\l__siunitx_number_uncert_offset_int=\count341
\l__siunitx_number_exponent_fixed_int=\count342
\l__siunitx_number_min_decimal_int=\count343
\l__siunitx_number_min_integer_int=\count344
\l__siunitx_number_round_precision_int=\count345
\l__siunitx_number_lower_threshold_int=\count346
```

```
\l__siunitx_number_upper_threshold_int=\count347
\l__siunitx_number_group_first_int=\count348
\l__siunitx_number_group_size_int=\count349
\l__siunitx_number_group_minimum_int=\count350
\l__siunitx_angle_tmp_dim=\dimen271
\l__siunitx_angle_marker_box=\box77
\l__siunitx_angle_unit_box=\box78
\l__siunitx_compound_count_int=\count351
(c:/texlive/2024/texmf-dist/tex/latex/translations/translations.sty
Package: translations 2022/02/05 v1.12 internationalization of LaTeX2e
packages
 (CN)
) (c:/texlive/2024/texmf-dist/tex/latex/amsmath/amstext.sty
Package: amstext 2021/08/26 v2.01 AMS text
(c:/texlive/2024/texmf-dist/tex/latex/amsmath/amsgen.sty
File: amsgen.sty 1999/11/30 v2.0 generic functions
\@emptytoks=\toks44
\ex@=\dimen272
))
\l__siunitx_table_tmp_box=\box79
\l__siunitx_table_tmp_dim=\dimen273
\l__siunitx_table_column_width_dim=\dimen274
\l__siunitx_table_integer_box=\box80
\l__siunitx_table_decimal_box=\box81
\l__siunitx_table_uncert_box=\box82
\l__siunitx_table_before_box=\box83
\l__siunitx_table_after_box=\box84
\l__siunitx_table_before_dim=\dimen275
\l__siunitx_table_carry_dim=\dimen276
\l__siunitx_unit_tmp_int=\count352
\l__siunitx_unit_position_int=\count353
\l__siunitx_unit_total_int=\count354
) (c:/texlive/2024/texmf-dist/tex/latex/lipsum/lipsum.sty
(c:/texlive/2024/texm
f-dist/tex/latex/l3packages/l3keys2e/l3keys2e.sty
Package: l3keys2e 2024-05-08 LaTeX2e option processing using LaTeX3 keys
)
Package: lipsum 2021-09-20 v2.7 150 paragraphs of Lorem Ipsum dummy text
\g__lipsum_par_int=\count355
\l__lipsum_a_int=\count356
\l__lipsum_b_int=\count357
(c:/texlive/2024/texmf-dist/tex/latex/lipsum/lipsum.ltd.tex))
(c:/texlive/2024/
texmf-dist/tex/latex/orcidlink/orcidlink.sty
Package: orcidlink 2024/06/26 v1.1.0 Support ORCID's three different ID
formats
.
(c:/texlive/2024/texmf-dist/tex/latex/hyperref/hyperref.sty
Package: hyperref 2024-07-10 v7.01j Hypertext links for LaTeX
(c:/texlive/2024/texmf-dist/tex/generic/pdfescape/pdfescape.sty
Package: pdfescape 2019/12/09 v1.15 Implements pdfTeX's escape features
(HO)
) (c:/texlive/2024/texmf-dist/tex/latex/hycolor/hycolor.sty
```

```
Package: hycolor 2020-01-27 v1.10 Color options for hyperref/bookmark
(HO)
) (c:/texlive/2024/texmf-dist/tex/latex/hyperref/nameref.sty
Package: nameref 2023-11-26 v2.56 Cross-referencing by name of section
(c:/texlive/2024/texmf-dist/tex/latex/refcount/refcount.sty
Package: refcount 2019/12/15 v3.6 Data extraction from label references
(HO)
) (c:/texlive/2024/texmf-
dist/tex/generic/gettitlestring/gettitlestring.sty
Package: gettitlestring 2019/12/15 v1.6 Cleanup title references (HO)
)
\c@section@level=\count358
) (c:/texlive/2024/texmf-dist/tex/generic/stringenc/stringenc.sty
Package: stringenc 2019/11/29 v1.12 Convert strings between diff.
encodings (HO
)
)
\@linkdim=\dimen277
\Hy@linkcounter=\count359
\Hy@pagecounter=\count360
(c:/texlive/2024/texmf-dist/tex/latex/hyperref/pd1enc.def
File: pd1enc.def 2024-07-10 v7.01j Hyperref: PDFDocEncoding definition
(HO)
Now handling font encoding PD1 ...
... no UTF-8 mapping file for font encoding PD1
)
\Hy@SavedSpaceFactor=\count361
(c:/texlive/2024/texmf-dist/tex/latex/hyperref/puenc.def
File: puenc.def 2024-07-10 v7.01j Hyperref: PDF Unicode definition (HO)
Now handling font encoding PU ...
... no UTF-8 mapping file for font encoding PU
)
Package hyperref Info: Option `colorlinks' set `true' on input line 4040.
Package hyperref Info: Hyper figures OFF on input line 4157.
Package hyperref Info: Link nesting OFF on input line 4162.
Package hyperref Info: Hyper index ON on input line 4165.
Package hyperref Info: Plain pages OFF on input line 4172.
Package hyperref Info: Backreferencing OFF on input line 4177.
Package hyperref Info: Implicit mode ON; LaTeX internals redefined.
Package hyperref Info: Bookmarks ON on input line 4424.
\c@Hy@tempcnt=\count362
LaTeX Info: Redefining \url on input line 4763.
\XeTeXLinkMargin=\dimen278
(c:/texlive/2024/texmf-dist/tex/generic/bitset/bitset.sty
Package: bitset 2019/12/09 v1.3 Handle bit-vector datatype (HO)
(c:/texlive/2024/texmf-dist/tex/generic/bigintcalc/bigintcalc.sty
Package: bigintcalc 2019/12/15 v1.5 Expandable calculations on big
integers (HO
)
))
\Fld@menulength=\count363
\Field@Width=\dimen279
\Fld@charsize=\dimen280
Package hyperref Info: Hyper figures OFF on input line 6042.
```

```
Package hyperref Info: Link nesting OFF on input line 6047.
Package hyperref Info: Hyper index ON on input line 6050.
Package hyperref Info: backreferencing OFF on input line 6057.
Package hyperref Info: Link coloring ON on input line 6060.
Package hyperref Info: Link coloring with OCG OFF on input line 6067.
Package hyperref Info: PDF/A mode OFF on input line 6072.
(c:/texlive/2024/texmf-dist/tex/latex/base/atbegshi-ltx.sty
Package: atbegshi-ltx 2021/01/10 v1.0c Emulation of the original atbegshi
package with kernel methods
)
\Hy@abspage=\count364
\c@Item=\count365
\c@Hfootnote=\count366
)
Package hyperref Info: Driver (autodetected): hpdftex.
(c:/texlive/2024/texmf-dist/tex/latex/hyperref/hpdftex.def
File: hpdftex.def 2024-07-10 v7.01j Hyperref driver for pdfTeX
(c:/texlive/2024/texmf-dist/tex/latex/base/atveryend-ltx.sty
Package: atveryend-ltx 2020/08/19 v1.0a Emulation of the original
atveryend pac
kage
with kernel methods
)
\HyAnn@Count=\count367
\Fld@listcount=\count368
\c@bookmark@seq@number=\count369
(c:/texlive/2024/texmf-dist/tex/latex/rerunfilecheck/rerunfilecheck.sty
Package: rerunfilecheck 2022-07-10 v1.10 Rerun checks for auxiliary files
(HO)
(c:/texlive/2024/texmf-dist/tex/generic/uniquecounter/uniquecounter.sty
Package: uniquecounter 2019/12/15 v1.4 Provide unlimited unique counter
(HO)
)
Package uniquecounter Info: New unique counter `rerunfilecheck' on input
line 2
85.
)
\Hy@SectionHShift=\skip165
) (c:/texlive/2024/texmf-dist/tex/latex/pgf/frontendlayer/tikz.sty
(c:/texlive/
2024/texmf-dist/tex/latex/pgf/basiclayer/pgf.sty (c:/texlive/2024/texmf-
dist/te
x/latex/pgf/utilities/pgfrcs.sty (c:/texlive/2024/texmf-
dist/tex/generic/pgf/ut
ilities/pgfutil-common.tex
\pgfutil@everybye=\toks45
\pgfutil@tempdima=\dimen281
\pgfutil@tempdimb=\dimen282
) (c:/texlive/2024/texmf-dist/tex/generic/pgf/utilities/pgfutil-latex.def
\pgfutil@abb=\box85
) (c:/texlive/2024/texmf-dist/tex/generic/pgf/utilities/pgfrcs.code.tex
(c:/tex
live/2024/texmf-dist/tex/generic/pgf/pgf.revision.tex)
Package: pgfrcs 2023-01-15 v3.1.10 (3.1.10)
```

```
))
Package: pgf 2023-01-15 v3.1.10 (3.1.10)
(c:/texlive/2024/texmf-dist/tex/latex/pgf/basiclayer/pgfcore.sty
(c:/texlive/20
24/texmf-dist/tex/latex/pgf/systemlayer/pgfsys.sty
(c:/texlive/2024/texmf-dist/
tex/generic/pgf/systemlayer/pgfsys.code.tex
Package: pgfsys 2023-01-15 v3.1.10 (3.1.10)
(c:/texlive/2024/texmf-dist/tex/generic/pgf/utilities/pgfkeys.code.tex
\pgfkeys@pathtoks=\toks46
\pgfkeys@temptoks=\toks47

(c:/texlive/2024/texmf-
dist/tex/generic/pgf/utilities/pgfkeyslibraryfiltered.co
de.tex
\pgfkeys@tmptoks=\toks48
))
\pgf@x=\dimen283
\pgf@y=\dimen284
\pgf@xa=\dimen285
\pgf@ya=\dimen286
\pgf@xb=\dimen287
\pgf@yb=\dimen288
\pgf@xc=\dimen289
\pgf@yc=\dimen290
\pgf@xd=\dimen291
\pgf@yd=\dimen292
\w@pgf@writea=\write5
\r@pgf@reada=\read4
\c@pgf@counta=\count370
\c@pgf@countb=\count371
\c@pgf@countc=\count372
\c@pgf@countd=\count373
\t@pgf@toka=\toks49
\t@pgf@tokb=\toks50
\t@pgf@tokc=\toks51
\pgf@sys@id@count=\count374
(c:/texlive/2024/texmf-dist/tex/generic/pgf/systemlayer/pgf.cfg
File: pgf.cfg 2023-01-15 v3.1.10 (3.1.10)
)
Driver file for pgf: pgfsys-pdftex.def
(c:/texlive/2024/texmf-dist/tex/generic/pgf/systemlayer/pgfsys-pdftex.def
File: pgfsys-pdftex.def 2023-01-15 v3.1.10 (3.1.10)
(c:/texlive/2024/texmf-dist/tex/generic/pgf/systemlayer/pgfsys-common-
pdf.def
File: pgfsys-common-pdf.def 2023-01-15 v3.1.10 (3.1.10)
)))
(c:/texlive/2024/texmf-
dist/tex/generic/pgf/systemlayer/pgfsyssoftpath.code.tex
File: pgfsyssoftpath.code.tex 2023-01-15 v3.1.10 (3.1.10)
\pgfsyssoftpath@smallbuffer@items=\count375
\pgfsyssoftpath@bigbuffer@items=\count376
)
```

```
(c:/texlive/2024/texmf-
dist/tex/generic/pgf/systemlayer/pgfsysprotocol.code.tex
File: pgfsysprotocol.code.tex 2023-01-15 v3.1.10 (3.1.10)
)) (c:/texlive/2024/texmf-
dist/tex/generic/pgf/basiclayer/pgfcore.code.tex
Package: pgfcore 2023-01-15 v3.1.10 (3.1.10)
(c:/texlive/2024/texmf-dist/tex/generic/pgf/math/pgfmath.code.tex
(c:/texlive/2
024/texmf-dist/tex/generic/pgf/math/pgfmathutil.code.tex)
(c:/texlive/2024/texm
f-dist/tex/generic/pgf/math/pgfmathparser.code.tex
\pgfmath@dimen=\dimen293
\pgfmath@count=\count377
\pgfmath@box=\box86
\pgfmath@toks=\toks52
\pgfmath@stack@operand=\toks53
\pgfmath@stack@operation=\toks54
) (c:/texlive/2024/texmf-
dist/tex/generic/pgf/math/pgfmathfunctions.code.tex)
(c:/texlive/2024/texmf-
dist/tex/generic/pgf/math/pgfmathfunctions.basic.code.te
x)
(c:/texlive/2024/texmf-
dist/tex/generic/pgf/math/pgfmathfunctions.trigonometric
.code.tex)
(c:/texlive/2024/texmf-
dist/tex/generic/pgf/math/pgfmathfunctions.random.code.t
ex)
(c:/texlive/2024/texmf-
dist/tex/generic/pgf/math/pgfmathfunctions.comparison.co
de.tex)
(c:/texlive/2024/texmf-
dist/tex/generic/pgf/math/pgfmathfunctions.base.code.tex
)
(c:/texlive/2024/texmf-
dist/tex/generic/pgf/math/pgfmathfunctions.round.code.te
x)
(c:/texlive/2024/texmf-
dist/tex/generic/pgf/math/pgfmathfunctions.misc.code.tex
)
(c:/texlive/2024/texmf-
dist/tex/generic/pgf/math/pgfmathfunctions.integerarithm
etics.code.tex) (c:/texlive/2024/texmf-
dist/tex/generic/pgf/math/pgfmathcalc.co
de.tex) (c:/texlive/2024/texmf-
dist/tex/generic/pgf/math/pgfmathfloat.code.tex
\c@pgfmathroundto@lastzeros=\count378
)) (c:/texlive/2024/texmf-dist/tex/generic/pgf/math/pgfint.code.tex)
(c:/texliv
e/2024/texmf-dist/tex/generic/pgf/basiclayer/pgfcorepoints.code.tex
File: pgfcorepoints.code.tex 2023-01-15 v3.1.10 (3.1.10)
\pgf@picminx=\dimen294
\pgf@picmaxx=\dimen295
\pgf@picminy=\dimen296
```

```
\pgf@picmaxy=\dimen297
\pgf@pathminx=\dimen298
\pgf@pathmaxx=\dimen299
\pgf@pathminy=\dimen300
\pgf@pathmaxy=\dimen301
\pgf@xx=\dimen302
\pgf@xy=\dimen303
\pgf@yx=\dimen304
\pgf@yy=\dimen305
\pgf@zx=\dimen306
\pgf@zy=\dimen307
)
(c:/texlive/2024/texmf-
dist/tex/generic/pgf/basiclayer/pgfcorepathconstruct.cod
e.tex
File: pgfcorepathconstruct.code.tex 2023-01-15 v3.1.10 (3.1.10)
\pgf@path@lastx=\dimen308
\pgf@path@lasty=\dimen309
)
(c:/texlive/2024/texmf-
dist/tex/generic/pgf/basiclayer/pgfcorepathusage.code.te
x
File: pgfcorepathusage.code.tex 2023-01-15 v3.1.10 (3.1.10)
\pgf@shorten@end@additional=\dimen310
\pgf@shorten@start@additional=\dimen311
) (c:/texlive/2024/texmf-
dist/tex/generic/pgf/basiclayer/pgfcorescopes.code.tex
File: pgfcorescopes.code.tex 2023-01-15 v3.1.10 (3.1.10)
\pgfpic=\box87
\pgf@hbox=\box88
\pgf@layerbox@main=\box89
\pgf@picture@serial@count=\count379
)
(c:/texlive/2024/texmf-
dist/tex/generic/pgf/basiclayer/pgfcoregraphicstate.code
.tex
File: pgfcoregraphicstate.code.tex 2023-01-15 v3.1.10 (3.1.10)
\pgflinewidth=\dimen312
)
(c:/texlive/2024/texmf-
dist/tex/generic/pgf/basiclayer/pgfcoretransformations.c
ode.tex
File: pgfcoretransformations.code.tex 2023-01-15 v3.1.10 (3.1.10)
\pgf@pt@x=\dimen313
\pgf@pt@y=\dimen314
\pgf@pt@temp=\dimen315
) (c:/texlive/2024/texmf-
dist/tex/generic/pgf/basiclayer/pgfcorequick.code.tex
File: pgfcorequick.code.tex 2023-01-15 v3.1.10 (3.1.10)
) (c:/texlive/2024/texmf-
dist/tex/generic/pgf/basiclayer/pgfcoreobjects.code.te
x
File: pgfcoreobjects.code.tex 2023-01-15 v3.1.10 (3.1.10)
)
```

```
(c:/texlive/2024/texmf-
dist/tex/generic/pgf/basiclayer/pgfcorepathprocessing.co
de.tex
File: pgfcorepathprocessing.code.tex 2023-01-15 v3.1.10 (3.1.10)
) (c:/texlive/2024/texmf-
dist/tex/generic/pgf/basiclayer/pgfcorearrows.code.tex
File: pgfcorearrows.code.tex 2023-01-15 v3.1.10 (3.1.10)
\pgfarrowsep=\dimen316
) (c:/texlive/2024/texmf-
dist/tex/generic/pgf/basiclayer/pgfcoreshade.code.tex
File: pgfcoreshade.code.tex 2023-01-15 v3.1.10 (3.1.10)
\pgf@max=\dimen317
\pgf@sys@shading@range@num=\count380
\pgf@shadingcount=\count381
) (c:/texlive/2024/texmf-
dist/tex/generic/pgf/basiclayer/pgfcoreimage.code.tex
File: pgfcoreimage.code.tex 2023-01-15 v3.1.10 (3.1.10)
)
(c:/texlive/2024/texmf-
dist/tex/generic/pgf/basiclayer/pgfcoreexternal.code.tex
File: pgfcoreexternal.code.tex 2023-01-15 v3.1.10 (3.1.10)
\pgfexternal@startupbox=\box90
) (c:/texlive/2024/texmf-
dist/tex/generic/pgf/basiclayer/pgfcorelayers.code.tex
File: pgfcorelayers.code.tex 2023-01-15 v3.1.10 (3.1.10)
)
(c:/texlive/2024/texmf-
dist/tex/generic/pgf/basiclayer/pgfcoretransparency.code
.tex
File: pgfcoretransparency.code.tex 2023-01-15 v3.1.10 (3.1.10)
)
(c:/texlive/2024/texmf-
dist/tex/generic/pgf/basiclayer/pgfcorepatterns.code.tex
File: pgfcorepatterns.code.tex 2023-01-15 v3.1.10 (3.1.10)
) (c:/texlive/2024/texmf-
dist/tex/generic/pgf/basiclayer/pgfcorerdf.code.tex
File: pgfcorerdf.code.tex 2023-01-15 v3.1.10 (3.1.10)
))) (c:/texlive/2024/texmf-
dist/tex/generic/pgf/modules/pgfmoduleshapes.code.te
x
File: pgfmoduleshapes.code.tex 2023-01-15 v3.1.10 (3.1.10)
\pgfnodeparttextbox=\box91
) (c:/texlive/2024/texmf-
dist/tex/generic/pgf/modules/pgfmoduleplot.code.tex
File: pgfmoduleplot.code.tex 2023-01-15 v3.1.10 (3.1.10)
)
(c:/texlive/2024/texmf-dist/tex/latex/pgf/compatibility/pgfcomp-version-
0-65.st
y
Package: pgfcomp-version-0-65 2023-01-15 v3.1.10 (3.1.10)
\pgf@nodesepstart=\dimen318
\pgf@nodesepend=\dimen319
)
```

```
(c:/texlive/2024/texmf-dist/tex/latex/pgf/compatibility/pgfcomp-version-
1-18.st
y
Package: pgfcomp-version-1-18 2023-01-15 v3.1.10 (3.1.10)
)) (c:/texlive/2024/texmf-dist/tex/latex/pgf/utilities/pgffor.sty
(c:/texlive/2
024/texmf-dist/tex/latex/pgf/utilities/pgfkeys.sty
(c:/texlive/2024/texmf-dist/
tex/generic/pgf/utilities/pgfkeys.code.tex)) (c:/texlive/2024/texmf-
dist/tex/la
tex/pgf/math/pgfmath.sty (c:/texlive/2024/texmf-
dist/tex/generic/pgf/math/pgfma
th.code.tex)) (c:/texlive/2024/texmf-
dist/tex/generic/pgf/utilities/pgffor.code
.tex
Package: pgffor 2023-01-15 v3.1.10 (3.1.10)
\pgffor@iter=\dimen320
\pgffor@skip=\dimen321
\pgffor@stack=\toks55
\pgffor@toks=\toks56
)) (c:/texlive/2024/texmf-
dist/tex/generic/pgf/frontendlayer/tikz/tikz.code.tex
Package: tikz 2023-01-15 v3.1.10 (3.1.10)

(c:/texlive/2024/texmf-
dist/tex/generic/pgf/libraries/pgflibraryplothandlers.co
de.tex
File: pgflibraryplothandlers.code.tex 2023-01-15 v3.1.10 (3.1.10)
\pgf@plot@mark@count=\count382
\pgfplotmarksize=\dimen322
)
\tikz@lastx=\dimen323
\tikz@lasty=\dimen324
\tikz@lastxsaved=\dimen325
\tikz@lastysaved=\dimen326
\tikz@lastmovetox=\dimen327
\tikz@lastmovetoy=\dimen328
\tikzleveldistance=\dimen329
\tikzsiblingdistance=\dimen330
\tikz@figbox=\box92
\tikz@figbox@bg=\box93
\tikz@tempbox=\box94
\tikz@tempbox@bg=\box95
\tikztreelevel=\count383
\tikznumberofchildren=\count384
\tikznumberofcurrentchild=\count385
\tikz@fig@count=\count386
(c:/texlive/2024/texmf-
dist/tex/generic/pgf/modules/pgfmodulematrix.code.tex
File: pgfmodulematrix.code.tex 2023-01-15 v3.1.10 (3.1.10)
\pgfmatrixcurrentrow=\count387
\pgfmatrixcurrentcolumn=\count388
\pgf@matrix@numberofcolumns=\count389
)
```

```
\tikz@expandcount=\count390

(c:/texlive/2024/texmf-
dist/tex/generic/pgf/frontendlayer/tikz/libraries/tikzli
brarytopaths.code.tex
File: tikzlibrarytopaths.code.tex 2023-01-15 v3.1.10 (3.1.10)
)))
(c:/texlive/2024/texmf-
dist/tex/generic/pgf/frontendlayer/tikz/libraries/tikzli
brarysvg.path.code.tex
File: tikzlibrarysvg.path.code.tex 2023-01-15 v3.1.10 (3.1.10)

(c:/texlive/2024/texmf-
dist/tex/generic/pgf/libraries/pgflibrarysvg.path.code.t
ex
File: pgflibrarysvg.path.code.tex 2023-01-15 v3.1.10 (3.1.10)
(c:/texlive/2024/texmf-
dist/tex/generic/pgf/modules/pgfmoduleparser.code.tex
File: pgfmoduleparser.code.tex 2023-01-15 v3.1.10 (3.1.10)
\pgfparserdef@arg@count=\count391
)
\pgf@lib@svg@last@x=\dimen331
\pgf@lib@svg@last@y=\dimen332
\pgf@lib@svg@last@c@x=\dimen333
\pgf@lib@svg@last@c@y=\dimen334
\pgf@lib@svg@count=\count392
\pgf@lib@svg@max@num=\count393
))
\@curXheight=\skip166
)
runsystem(for ^%i in (pygmentize.exe pygmentize.bat pygmentize.cmd) do
set > ma
in.aex <nul: /p x=^%~$PATH:i>> main.aex)...disabled (restricted).

! Emergency stop.
<read 3>

l.70 \begin{document}
                     ^^M
*** (cannot \read from terminal in nonstop modes)


Here is how much of TeX's memory you used:
 36127 strings out of 473583
 724181 string characters out of 5732343
 1926908 words of memory out of 5000000
 58445 multiletter control sequences out of 15000+600000
 560290 words of font info for 40 fonts, out of 8000000 for 9000
 1141 hyphenation exceptions out of 8191
 123i,0n,131p,472b,152s stack positions out of
10000i,1000n,20000p,200000b,200000s
!  ==> Fatal error occurred, no output PDF file produced!
```

Manuscript

Click here to access/download;Manuscript;GigaScience_datanote.pdf

(GIGA)^n SCIENCE

GigaScience, 2024, 1–15

doi: xx.xxxx/xxxx
Manuscript in Preparation
Data Note

DATA NOTE

SurGen: 1020 H&E-stained Whole Slide Images With Survival and Genetic Markers

Craig Myles[1,*], In Hwa Um[2], Craig Marshall[3], David Harris-Birtill[1] and David J Harrison[2,4]

[1]School of Computer Science, University of St Andrews, St Andrews, KY16 9SX, UK and [2]School of Medicine, University of St Andrews, St Andrews, KY16 9TF, UK and [3]Lothian Biorepository, NHS Lothian, Edinburgh EH16 4SA, UK and [4]NHS Lothian Pathology, Division of Laboratory Medicine, Royal Infirmary of Edinburgh, Edinburgh EH16 4SA, UK

[*]cggm1@st-andrews.ac.uk; https://orcid.org/0000-0002-2701-3149

Abstract

Background: Cancer remains one of the leading causes of morbidity and mortality worldwide. Comprehensive datasets that combine histopathological images with genetic and survival data across various tumour sites are essential for advancing computational pathology and personalised medicine. **Results**: We present SurGen, a dataset comprising 1,020 H&E-stained whole slide images (WSIs) from 843 colorectal cancer cases. The dataset includes detailed annotations for key genetic mutations (*KRAS*, *NRAS*, *BRAF*) and mismatch repair status, as well as survival data for 426 cases. We illustrate SurGen's utility with a proof-of-concept model that predicts mismatch-repair status directly from WSIs, achieving a test AUROC of 0.8273. These preliminary results underscore the dataset's potential to facilitate research in biomarker discovery, prognostic modelling, and advanced machine learning applications in colorectal cancer and beyond. **Conclusions**: SurGen offers a valuable resource for the scientific community, enabling studies that require high-quality WSIs linked with comprehensive clinical and genetic information on colorectal cancer. Our initial findings affirm the dataset's capacity to advance diagnostic precision and foster the development of personalised treatment strategies in colorectal oncology. Data available online: https://doi.org/10.6019/S-BIAD1285.

Key words: whole slide image (WSI); haematoxylin and eosin (H&E) stain; mismatch repair (MMR); microsatellite instability (MSI); KRAS mutation; NRAS mutation; BRAF mutation; colorectal cancer; digital pathology; dataset;

Background

Colorectal cancer is among the most common and lethal cancers worldwide with over 900,000 deaths occurring each year [1, 2]. Advances in computational pathology and machine learning have the potential to revolutionise cancer diagnosis and treatment by enabling the analysis of complex histopathological and genetic data across various tumour types [3, 4].

High-quality datasets that combine whole slide images (WSIs) with detailed clinical and genetic annotations are crucial for developing and validating computational models. However, the field currently faces significant limitations due to the scarcity of publicly available annotated datasets that integrate both imaging and non-imaging patient data [5]. Existing datasets often focus on specific cancer sites, such as breast [6, 7, 8], gastric and colorectal [9, 10], and lung [11], or lack comprehensive annotations necessary for advanced computational pathology research. Additionally, the quality of publicly available samples can be highly variable, potentially hindering the development of robust and generalisable models [5]. The SurGen dataset addresses these gaps by providing a diverse and high-quality collection of WSIs linked with genetic mutations, mismatch repair status, and cancer staging across colorectal and neighbouring sites. Additionally, it includes survival data specifically for the primary colorectal cancer cohort, enhancing its value for prognostic studies in this prevalent cancer.

This article reports on the composition, collection, and potential

Compiled on: June 20, 2025.
Draft manuscript prepared by the author.

Table 1. Comparative overview of publicly available formalin-fixed-paraffin-embedded (FFPE) H&E stained colorectal whole slide image datasets with relevant biomarker labels.

Dataset	Access	Origin	Cases	WSIs	Magnification	MPP	KRAS	NRAS	BRAF	MSI/MMR	Survival	Staging	Pathological	Segmentation
SurGen (Ours)	Public	GBR	843	1020	40X	0.1112	✓	✓	✓	✓	✓	✓	✓	✗
PAIP [10]	Upon Request	KOR	118	118	40X	0.2522	✗	✗	✗	✓	✗	✗	✗	✓
TCGA-COAD [13]	Public	USA	451	459	20X or 40X	*0.2436	✓	✓	✓	✓	✓	✓	✓	✗
TCGA-READ [13]	Public	USA	164	165	20X or 40X	*0.2427	✓	✓	✓	✓	✓	✓	✓	✗
CPTAC-COAD [14]	Public	USA	105	220	40X	0.2501	✓	✓	✓	✓	✗	✓	✓	✗
CRC-Orion [15]	Public	USA	40	42	20x	0.3250	✓	✓	✓	✓	✓	✓	✓	✓

Note: cases are only counted if at least one diagnostic whole slide image (WSI) is available per clinical record. Note that reported case counts may differ across publications due to varying inclusion criteria and filtering methods. This table does not include any tumour microarray (TMA) or patch-based datasets. MPP = Microns per pixel. MPP values marked with * are mean values across the cohort, with ranges: TCGA-COAD (0.2325-0.2527), TCGA-READ (0.2325-0.2520).

applications of the SurGen dataset, highlighting its utility for both focused studies specific to primary colorectal cancer and broader investigations into metastatic tumour sites. This is particularly pertinent given that up to 50% of patients with localised disease eventually develop metastases [12].

The SurGen dataset is a comprehensive digital pathology resource designed to support a wide range of cancer and computational pathology research initiatives. It consists of whole slide images (WSIs) coupled with detailed clinical and genetic data, spanning colorectal regions as well as neighbouring metastatic sites. See table 2 for a breakdown of tumour sites across the SurGen dataset. The dataset is divided into two distinct subsets:

i. **SR386** (**Colorectal Cohort with Survival Data**) focuses on primary colorectal cancer, consisting of 427 WSIs from 427 cases with a focus on colorectal tumour sites. This subset includes survival data in addition to biomarker labels, such as mutation status in the *KRAS*, *NRAS*, and *BRAF* genes, as well as mismatch repair (MMR) status. This makes it particularly valuable for research aimed at understanding the genetic and biomarker properties of colorectal cancer for the exploration and prediction of its clinical outcomes.

ii. **SR1482** (**Colorectal Cancer with Metastatic Sites**) is a subset that contains 593 WSIs from 416 colorectal cancer cases. This cohort includes WSIs from both primary colorectal tumours and metastatic lesions in sites such as the liver, lung, peritoneum, and others. While it does not include survival data, it offers extensive biomarker information, making it valuable for studies on genetic and molecular characteristics of colorectal cancer and its metastatic behaviour.

The SurGen dataset aims to facilitate research in oncology and digital pathology by providing high-quality, labelled WSIs that can be used for training and validating computational models, investigating tumour and oncological properties, and exploring biomarker-driven stratification in colorectal cancer. This article reports on the composition, collection, and potential applications of the SurGen dataset, highlighting its utility for both focused studies on colorectal cancer and broader investigations into neighbouring metastatic sites and generalised oncological understanding.

To highlight the comprehensive nature of the SurGen dataset, we compare it with several publicly available colorectal cancer datasets. Table 1 summarises key attributes such as the inclusion of genetic markers, survival data, and tumour staging.

Other digital-pathology collections also exist, for instance, the PLCO Cancer Screening Trial [16] offers controlled access to colorectal WSIs (approximately 2,800 images from 749 cases) but does not provide tumour level molecular annotation, while the open-access HunCRC [17] biopsy dataset contains 200 annotated slides yet lacks molecular and survival data.

As shown in Table 1, the SurGen dataset provides a valuable addition to publicly available resources, uniquely integrating high-resolution WSIs with detailed genetic, clinical, and survival data. While datasets such as TCGA-COAD, TCGA-READ, and CPTAC-CRC offer comprehensive genomic sequencing data, SurGen complements these resources by focusing on key colorectal cancer biomarkers (*KRAS*, *NRAS*, *BRAF*, MSI/MMR) and survival outcomes. Moreover, its consistent high-resolution scanning at 40× magnification across all slides ensures uniform image quality, addressing variability seen in some datasets, such as TCGA.

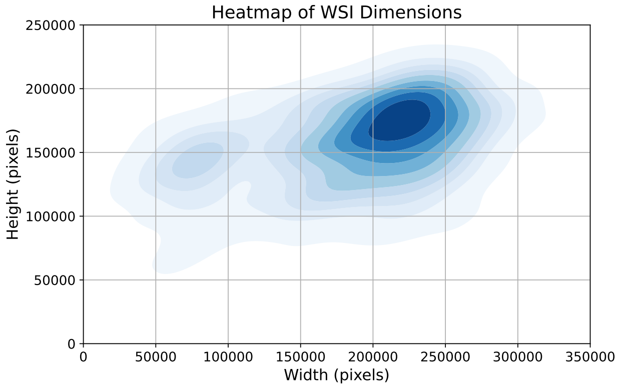

Figure 1. Heatmap of WSI dimensions (in pixels) across the SurGen dataset, illustrating the variability in image sizes due to differing tissue sample areas.

SurGen is among the largest publicly available colorectal cancer WSI datasets, with 1,020 slides from 843 cases, exceeding the combined slide count of TCGA-COAD, TCGA-READ, and CPTAC-CRC. While SurGen's genomic annotation is focused on specific biomarkers, its scale, resolution, and inclusion of survival data make it particularly well-suited for computational pathology research, prognostic modelling, and biomarker classification in colorectal cancer.

Data Description

This section provides an overview of the SurGen dataset, which includes whole slide images (WSIs) and corresponding clinical and genetic data. The dataset is intended to support research in cancer and computational pathology, offering a resource for studying genetic mutations, mismatch repair status, and patient survival outcomes. Below is a detailed description of the data and its collection process.

Each WSI in the SurGen dataset is scanned at 40× (0.1112μm per pixel) magnification, resulting in ultra-high-resolution images with pixel dimensions averaging 189,662 × 156,059 pixels. Figure 1 illustrates the spread of WSI dimensions across the SurGen dataset. The digital pathology images are stored in the CZI file format, which supports hierarchical pyramidal data structures for efficient storage and retrieval. Figure 2 demonstrates the level of granularity accessible via the ultra-high-resolution WSIs.

Patient Demographic

The SurGen dataset comprises clinical information from 843 cases, with patients ranging from 19 to 97 years of age (mean age = 64.58,

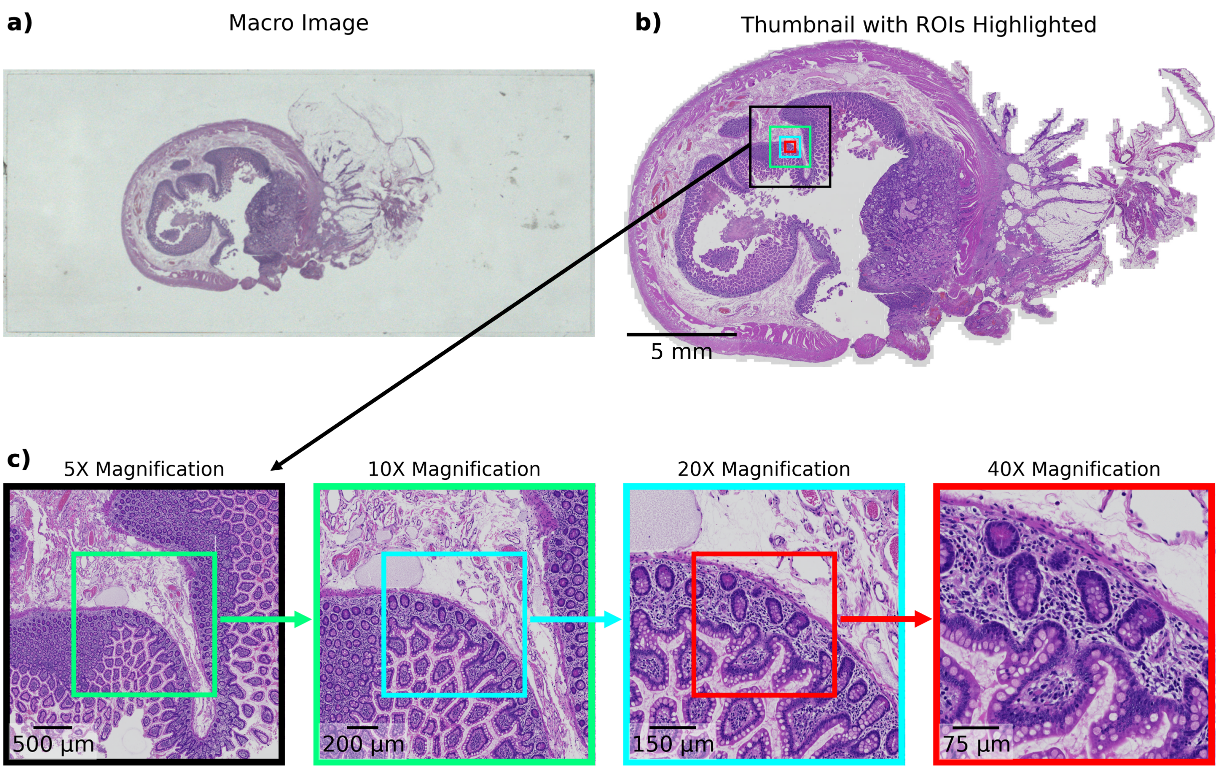

Figure 2. Hierarchical zoom visualisation of case SR1482_T412 with dimensions 242,506 × 134,026 pixels, corresponding to 26,974.20 × 14,907.85 μm. A) A low-resolution macro image of the whole slide, providing full anatomical context. B) Digitised whole slide image viewed at low-magnification. C) Successive zoom-ins of the selected region from b), providing increased granularity, enabling detailed examination of tissue structures while retaining the broader context. This hierarchical approach allows comprehensive visual exploration of tissue characteristics at varying scales. In practice, the pyramid levels are typically generated via Gaussian down-sampling to simulate various levels of magnification but enable an immediate interface for retrieving images at varying resolutions.

Table 2. Tumour Site Counts for SurGen, SR386, and SR1482

Tumour Site	SurGen	SR386	SR1482
Rectum	276	166	110
Sigmoid Colon	142	89	53
Caecum	118	64	54
Ascending Colon	99	43	56
Transverse Colon	46	25	21
Liver	38	0	38
Descending Colon	33	16	17
Splenic Flexure	22	14	8
Hepatic Flexure	16	7	9
Peritoneum/Omentum	16	0	16
Appendix	9	1	8
Lung	4	0	4
Lymph Nodes	4	0	4
Small Bowel	3	0	3
Bladder	3	0	3
Gall Bladder	2	0	2
Pelvis	2	0	2
Site Unknown	2	2	0
Kidney	1	0	1
Throat/Vocal Cords	1	0	1
Adrenal Gland	1	0	1
Umbilical Area	1	0	1
Spine	1	0	1
Perineal Area	1	0	1
Duodenum	1	0	1
Ureter	1	0	1

Note: Green-shaded cells indicate tumour sites within the top cumulative ranges of approximately 90% for each respective dataset (SurGen, SR386, and SR1482). The exact highlighted cumulative totals are 91.81%, 90.63%, and 91.83%, respectively. These sites collectively account for the majority of tumour occurrences in each dataset.

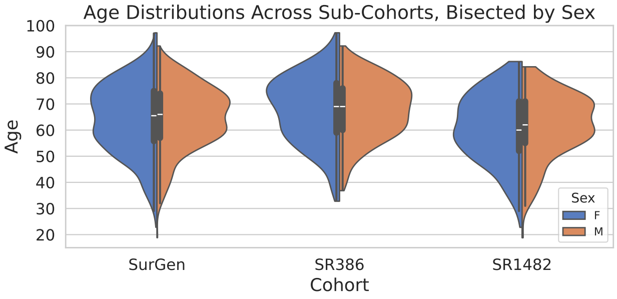

Figure 3. Illustration of the age distributions (in years) of patients in each cohort, split by sex. The width of each violin represents the density of data points at different ages, highlighting the distributions within and across the cohorts.

SD = ±12.73), as illustrated in Figure 3. The cohort comprises 46% females and 54% males.

Patient Survival

Survival data is available for the SR386 cohort, providing insights into patient outcomes over a five-year period following diagnosis. The dataset includes binary labels indicating whether a patient survived beyond the duration of the study, as well as the number of days until death for those who did not. For patients who outlived the study period or whose survival extends beyond the recorded date, their exact number of days until death is not captured, resulting in right-censoring.

Within the SR386 cohort, 161 patients (38%) died during the study period, while 264 patients (62%) were alive at the end of the study period. This distribution provides a general overview of pa-

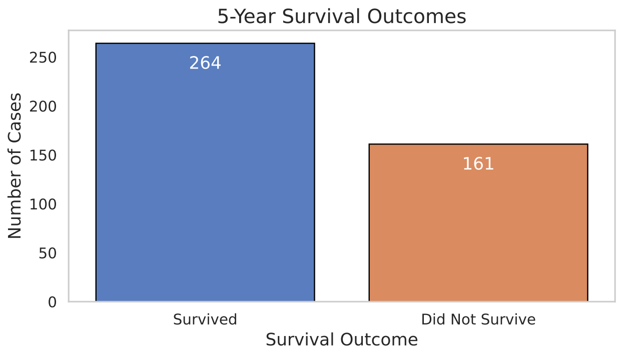

Figure 4. Bar chart depicting the 5-year survival outcomes of the SR386 cohort. The chart shows the number of individuals who survived (n=264) versus those who did not survive (n=161) within the 5-year period following diagnosis. The data excludes instances where survival status was not recorded (NULL values).

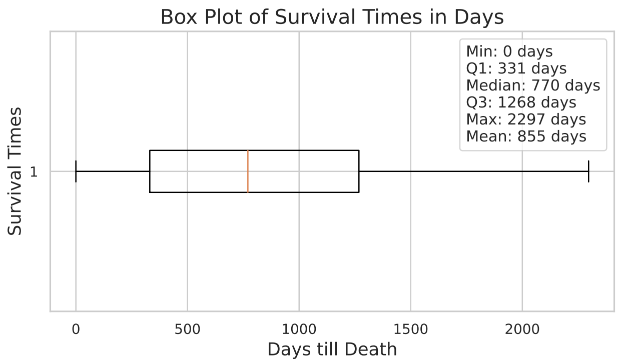

Figure 6. Box plot showing the distribution of survival times (in days) for cases in the SR386 cohort with recorded days till death. The plot illustrates key summary statistics, including the mean, minimum, first quartile (Q1), median, third quartile (Q3), and maximum survival times.

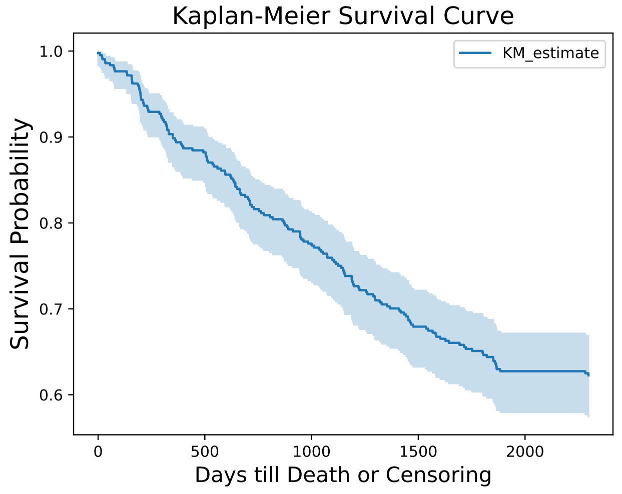

Figure 5. Kaplan-Meier survival curve illustrating estimated survival probabilities over time. Censoring occurred for patients who survived beyond the 5-year study duration, as they were not followed further. The curve reflects the proportion of individuals surviving at each time point, with 95% confidence intervals representing the uncertainty in these estimates.

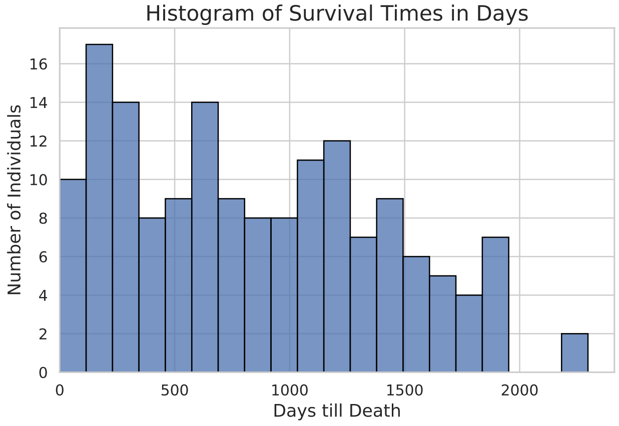

Figure 7. Histogram showing the distribution of survival times (in days) for cases in the SR386 cohort with recorded days until death. The x-axis represents the number of days until death, and the y-axis indicates the number of individuals who died within each time interval.

tient outcomes in the cohort. An overview of the binarised five-year survival outcomes is presented in Figure 4. CRC was the primary cause of death in 67 out of 161 deceased patients, accounting for 41.61% of all deaths in the cohort.

To visualise the survival probabilities over time, a Kaplan-Meier survival curve was constructed for the SR386 cohort, as shown in Figure 5. This curve illustrates the proportion of patients surviving at each time point during the study period. The gradual decline in the curve represents the decreasing number of patients alive as time progresses.

For the patients who did not survive beyond the study period, we analysed the distribution of their survival times. Figure 6 presents a box plot summarising key statistics of these survival times in days. The plot shows the minimum, first quartile (Q1), median, third quartile (Q3), maximum, and mean survival times. Specifically, the median survival time was 770 days, indicating that half of the patients who died did so within this number of days post-diagnosis.

Additionally, Figure 7 displays a histogram of the survival times for patients who died within the study period. The histogram shows how many patients died within specific time intervals, providing an overview of the distribution of survival times among these patients.

Due to quality control measures, missing information, or data inconsistencies, certain cases (i.e. 004, 208, 430) have been redacted or marked as '*NULL*' with respect to survival. However, these cases remain in the dataset as they contain valuable genetic information that can be utilised for separate predictive tasks.

Genetic Mutations

The SurGen dataset includes ground truth labels for key genetic mutations in the *KRAS*, *NRAS*, and *BRAF* genes, as well as mismatch repair (MMR) status and/or microsatellite instability (MSI). Figure 8 presents the distribution of these genetic mutations by sex. These genetic markers are crucial for understanding the molecular characteristics of tumours and their potential response to targeted therapies. Below, each mutation is discussed in detail.

BRAF Mutation: Present in 12.34% of SurGen cases, aligning with frequencies reported in the literature, which range from 3.5% to 13% [18, 19, 20, 21, 22]. BRAF mutations are critical in the MAPK/ERK signalling pathway and are significant targets for therapeutic intervention [23, 24].

KRAS Mutation: Present in 38.43% of SurGen cases, consistent with the range reported in other studies, from 37% to 46.4% [25, 18, 19, 20, 21]. KRAS is a proto-oncogene involved in cell signalling pathways that regulate cell growth and death. Mutations in KRAS are often linked to resistance to specific therapies, highlighting the importance of their identification for effective treatment planning [25].

NRAS Mutation: Observed in 3.80% of SurGen cases, this falls within the range of 2.6% to 9% reported across various studies [19,

22, 20, 21]. Like KRAS, NRAS mutations can influence treatment options and prognosis, though NRAS mutations are less common.

Mismatch Repair Deficiency and Microsatellite Instability

Mismatch repair deficiency (dMMR) and microsatellite instability (MSI) are critical genetic features in many cancers, particularly colorectal cancer [26]. dMMR occurs when the mismatch repair system, which normally corrects DNA replication errors, is compromised. This deficiency leads to an accumulation of mutations, particularly in regions of repetitive DNA known as microsatellites. When these microsatellites become unstable due to dMMR, the condition is termed microsatellite instability (MSI)[27, 26].

MSI is a key biomarker used to assess cancer prognosis and predict responses to certain therapies, such as immunotherapy. Tumours exhibiting high levels of MSI (MSI-high) are often associated with a better prognosis and may respond favourably to immune checkpoint inhibitors [28, 29]. Identifying MMR and MSI status is essential for developing targeted treatment strategies and improving patient outcomes.

Importantly, dMMR and MSI are hallmark features of Lynch syndrome (LS), the most common hereditary colorectal cancer predisposition syndrome, accounting for approximately 3% of all colorectal cancers [30, 31]. LS, also known as hereditary non-polyposis colorectal cancer (HNPCC), is caused by germline mutations in the MMR genes (*MLH1*, *MSH2*, *MSH6*, and *PMS2*) [32], leading to a higher risk of developing colorectal cancer and other cancers at a younger age. Identifying patients with dMMR/MSI can therefore aid in diagnosing Lynch syndrome and facilitating genetic counselling [33].

In our study, the assessment of MMR status and MSI status differed between the SR386 and SR1482 cohorts.

Assessment of MMR and MSI Status in Cohorts

While the SR386 cohort reports MMR status assessed through immunohistochemistry (IHC) for key MMR proteins, the SR1482 cohort reports both MMR and MSI status.

SR386 Cohort. In the SR386 cohort, MMR status was assessed exclusively using immunohistochemistry (IHC) for two key MMR proteins; MLH1 and PMS2. Cases were labelled according to the specific loss of expression observed. Primary antibodies against MLH1 and PMS2 were applied, and loss of nuclear staining in tumour cells for any of these MMR proteins was recorded.

SR1482 Cohort. In the SR1482 cohort, MSI status was determined using either immunohistochemistry (IHC) for MMR proteins (MLH1, MSH2, MSH6, PMS2) or PCR-based fragment analysis. For the PCR-based approach, the Promega Oncomate™ kit was utilised according to the manufacturer's recommended protocol. Cases were classified as MSI/dMMR if they showed evidence of microsatellite instability through PCR analysis or a loss of protein expression by IHC.

Mismatch Repair

Mismatch repair (MMR) status is available for most cases, with a distinction between microsatellite stable (MSS/pMMR) and microsatellite unstable (MSI/dMMR) tumours within the SR1482 dataset. This information is crucial for identifying patients who might benefit from immunotherapy [28, 27].

Microsatellites

Microsatellite instability (MSI) is a condition of genetic hypermutability that results from impaired DNA mismatch repair (MMR). Identifying MSI is important as it has implications for the prognosis and treatment of cancer.

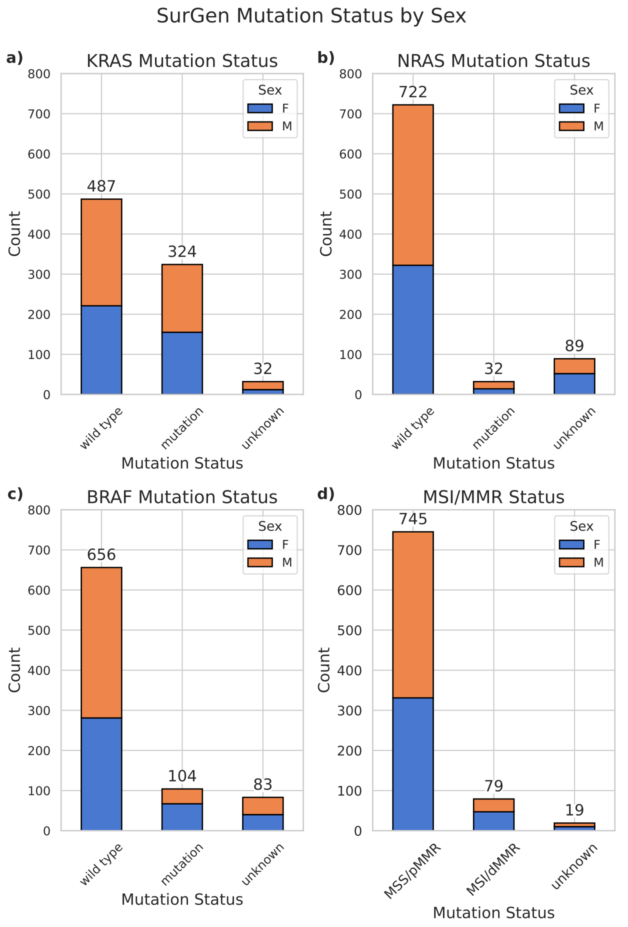

Figure 8. Bar chart depicting mutation prevalence across the SurGen dataset, highlighting mutation status of patients across a) KRAS, b) NRAS, c) BRAF, and d) MSI/MMR.

Staging

Tumour staging is a critical aspect of cancer diagnosis and treatment planning, providing a framework for assessing the extent of cancer spread within the body. Staging systems help in predicting patient prognosis, guiding treatment decisions, and enabling comparisons across clinical studies and populations [34]. Two widely used staging systems in colorectal cancer are the Dukes' staging system[35] and the TNM (Tumour, Node, Metastasis) staging system[36], each offering distinct advantages and serving different clinical needs.

The Dukes' staging system is one of the earliest methods used to classify the extent of colorectal cancer. It is relatively simple and easy to apply, making it useful for broad clinical assessments. However, although it includes stages for lymph node involvement (Stage C) and distant metastasis (Stage D), its simplicity limits its ability to provide more detailed, granular information on tumour characteristics [37].

The TNM staging system, in contrast, is more detailed and widely applicable across various cancer types. It provides a comprehensive classification based on the size and extent of the primary tumour (T), the involvement of regional lymph nodes (N), and the presence of distant metastasis (M). This system is advantageous for its specificity and adaptability to different cancers, though it can be more complex to use compared to the Dukes' system.

These staging systems are integral to clinical guidelines, informing treatment strategies such as surgical intervention, chemotherapy, and targeted therapies based on the stage of cancer.

The SurGen dataset includes tumour staging information using

Table 3. Overview of the SurGen dataset with respective technical, clinical, and mutational characteristic breakdown of the sub-sets SR386 and SR1482. Note: MSI/MMR ground truth was determined using Immunohistochemistry (IHC) or Polymerase Chain Reaction (PCR).

	**SurGen Dataset**	SR386	SR1482
Origin	Scotland	Scotland	Scotland
Number of cases	843	427	416
Number of WSIs	1020	427	593
WSI file format	.CZI	.CZI	.CZI
Magnification	40X	40X	40X
Microns per pixel (pixel width)	0.1112μm	0.1112μm	0.1112μm
Mean age (std. dev.)	64.58 (±12.73)	67.89 (±12.00)	61.20 (±12.59)
Female, n (%)	388 (46.03%)	197 (46.14%)	191 (45.91%)
Male, n (%)	455 (53.97%)	230 (53.86%)	225 (54.09%)
MSI/MMR ground truth	PCR/IHC	IHC	PCR/IHC
MSI/dMMR, n (%)	79 (9.37%)	32 (7.49%)	47 (11.30%)
MSS/pMMR, n (%)	745 (88.37%)	395 (92.51%)	350 (84.13%)
MSI/MMR status unknown, n (%)	19 (2.25%)	0 (0%)	19 (4.57%)
Five year survival (true), n (%)	264 (31.32%)	264 (61.83%)	0 (0%)
Five year survival (false), n (%)	162 (19.22%)	162 (37.94%)	0 (0%)
Five year survival (unreported), n (%)	417 (49.47%)	1 (0.23%)	416 (100%)
BRAF mutation, n (%)	104 (12.34%)	47 (11.00%)	57 (13.70%)
BRAF wild type, n (%)	656 (77.82%)	379 (88.76%)	277 (66.59%)
BRAF status unknown, n (%)	83 (9.85%)	1 (0.23%)	82 (19.71%)
KRAS mutation, n (%)	324 (38.43%)	147 (34.43%)	177 (42.55%)
KRAS wild type, n (%)	487 (57.77%)	266 (62.30%)	221 (53.12%)
KRAS status unknown, n (%)	32 (3.80%)	14 (3.26)	18 (4.33%)
NRAS mutation, n (%)	32 (3.80%)	16 (3.75%)	16 (3.85%)
NRAS wild type, n (%)	722 (85.65%)	399 (93.44%)	323 (77.64%)
NRAS status unknown, n (%)	89 (10.56%)	12 (2.81%)	77 (18.51%)

both the Dukes' and TNM staging systems, which are essential for correlating clinical outcomes with tumour progression. Understanding the distribution of these stages across the cohort can offer valuable insights into the disease dynamics within the study population.

Tumour Staging with Dukes'

The Dukes' staging system classifies colorectal cancer into four stages (A, B, C, and D), based on the extent of tumour invasion and the presence of lymph node involvement or distant metastasis [35]. Stage A represents the earliest form of cancer, confined to the mucosa, while Stage D indicates advanced disease with distant metastasis. This system, though less detailed than TNM, provides a quick and accessible way to gauge tumour progression and patient prognosis.

TNM Staging

The TNM staging system is a more granular approach that classifies cancer based on three key components: the size and extent of the primary tumour (T), the involvement of regional lymph nodes (N), and the presence of distant metastasis (M) [38]. Each of these components is assigned a score, and the combination of these scores determines the overall stage of the cancer, ranging from Stage 0 (in situ, non-invasive cancer) to Stage IV (advanced cancer with distant metastasis).

A comprehensive summary of the SurGen including survival data, genetic mutations, and image properties for both the SR386 and SR1482 sub-cohorts is provided in Table 3.

Data collection

Tissue Sample Preparation

Samples underwent formalin-fixed-paraffin-embedding (FFPE) processing. This involved fixing tissue specimen in formalin to preserve cellular structures and proteins, followed by embedding the samples in paraffin wax.

Once FFPE samples were prepared, they were processed using a microtome set to section at 5μm before being laid onto a glass slide. These slides were then subjected to routine haematoxylin and eosin (H&E) staining prior to their digitisation.

Slides were first immersed in haematoxylin, which stains the cell nuclei blue-purple. Following a rinse, slides were stained with eosin, which stains the cytoplasm and extracellular matrix pink. After staining, the slides underwent a dehydration process involving graded alcohols and xylene. Coverslips were subsequently applied with a mounting medium to preserve the stained sections.

Tissue Sample Digitisation

Prepared slides were digitised on-site using a *ZEISS Axio Scan.Z1 Microscopy Slide Scanner* at 40× magnification, equipped with a Plan-Apochromat 40x/0.95 Korr M27 objective lens. This combination produces a pixel size of 0.1112μm. The scans were performed using ZEN 2.6 (blue edition) software, capturing brightfield images with controlled transmitted light illumination. Digitised images were saved in 24-bit BGR format (BGR24) with a pixel size of 0.1112μm. A multi-resolution pyramidal image structure was generated, with each subsequent layer downsampled by a factor of 2 relative to the previous layer, using Gaussian filtering to maintain image quality. Figure 9 illustrates the WSI pixel counts across the SurGen dataset.

While this imaging setup yields an ultra-high resolution of 0.1112 μm per pixel, it is important to note that pixel size is not standardised across all digital pathology platforms. Scanners from other vendors (e.g., Philips, Hamamatsu, Leica) may report the same nominal magnification (e.g., 40×), yet produce images with coarser resolutions, typically around 0.25 μm per pixel, due to differences in objective lens, camera sensors, and internal optics. As such, magnification alone is an imprecise descriptor of image resolution. Reporting the true microns-per-pixel (MPP) value provides a more objective and reproducible measure of resolution, enabling proper normalisation across datasets acquired using differing hardware configurations.

For both cohorts, each whole slide image was digitised from the same formalin-fixed paraffin-embedded (FFPE) tissue block used for biomarker assessment, ensuring correspondence between the histological and molecular data.

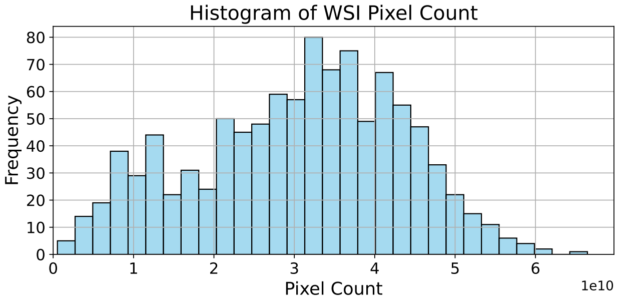

Figure 9. Histogram illustrating the scale of SurGen whole slide images with respect to the number of pixels per image. The x-axis represents the total number of pixels in each image (in tens of billions, 1×10^{10}), while the y-axis indicates the frequency of occurrence of images within each bin. The distribution shows the variability in the sizes of whole slide images across the dataset.

DNA Sequencing
Next Generation Sequencing (NGS) was performed to determine the mutation status of KRAS, NRAS, and BRAF using the Ion Torrent™ Cancer Hotspot Panel v2 (Thermo Fisher Scientific), following the manufacturer's protocol.

Data Curation and Quality Control

To ensure the quality and reliability of the SurGen dataset, we implemented several data curation and quality control measures.

Slide Quality Assessment
All WSIs were reviewed by specialised laboratory personnel trained in the preparation of tissue samples for microscopic examination. Each slide was assessed for staining quality, focus, and absence of artifacts. Slides that did not meet acceptable standards were re-scanned or re-prepared to improve image quality.

Data Alignment and Consistency
To maintain data integrity and maximise the utility of the dataset, we carefully matched each WSI with its corresponding clinical and genetic data. WSIs without any matching clinical data were excluded from the dataset, as clinical context is essential for meaningful analyses. However, clinical data entries were retained even if some fields were incomplete, provided they had a corresponding WSI. This approach ensured that all included WSIs had associated clinical information, enhancing the dataset's applicability while acknowledging that some clinical records might have missing data points.

Anonymisation and Ethical Considerations
Patient confidentiality was prioritised throughout the curation of the SurGen dataset. In line with contemporary data ethics in computational pathology [5, 39], we implemented deidentification protocols to ensure privacy while maximising data utility. Recognising that medical images potentially carry the risk of re-identification when combined with external data sources, our anonymisation strategy involved the removal or redaction of potentially identifiable information, including dates of diagnosis, date of death, and treatment details.

Data Use

Researchers can interact with the WSIs using tools such as OpenSlide [40], pylibCZIrw [41], and Bioformats [42]. The images are saved in a hierarchical pyramidal format, facilitating efficient viewing and processing at multiple resolutions. Software such as QuPath [43], Fiji [44], ImageJ [45], and others can be used to visualise and analyse these images.

To illustrate SurGen's practical utility, we provide a simple Python example for extracting a region of interest from a whole slide image. A Python script was implemented using `pylibCZIrw` (See Supplementary File S1). The script illustrates the process of identifying the centre of the WSI and extracting a 2048 × 2048 pixel region of interest (ROI) at full resolution. The extracted tile (Figure 10) provides a high-resolution view from the WSI, showcasing the potential for downstream analyses or tasks, such as patch-level feature extraction or visualisation.

Data re-use Potential

The SurGen dataset offers extensive opportunities for researchers in computational pathology and oncology. Its comprehensive collection of WSIs, coupled with genetic and other clinical annotations, makes it a valuable resource for various applications.

Firstly, the dataset can be utilised to train machine learning models for predicting mismatch repair (MMR) status and microsatellite instability (MSI). Given that existing publicly available datasets focusing on MSI/MMR prediction are limited, SurGen fills a crucial gap. Researchers can leverage this dataset to develop and validate models that may enhance diagnostic accuracy and inform treatment strategies, particularly in colorectal cancer where MSI status is a key prognostic and therapeutic marker.

Secondly, SurGen provides a rich resource for training models aimed at genomic mutation prediction, specifically for mutations in the KRAS, NRAS, and BRAF genes. Expanding the quantity of publicly available datasets with such detailed genetic information is immensely valuable, as it enables the development of models that can predict genetic mutations from histopathological images. This can potentially streamline the diagnostic process by reducing the need for costly and time-consuming genetic testing.

Furthermore, the high-quality WSIs in the SurGen dataset make it suitable for training foundation models in digital pathology. Existing works have demonstrated that the performance of these models improves with the availability of larger and more diverse datasets [46, 47, 48]. By contributing to the training of such models, SurGen can aid in advancing the field of computational pathology, facilitating the development of algorithms that are more robust and generalisable.

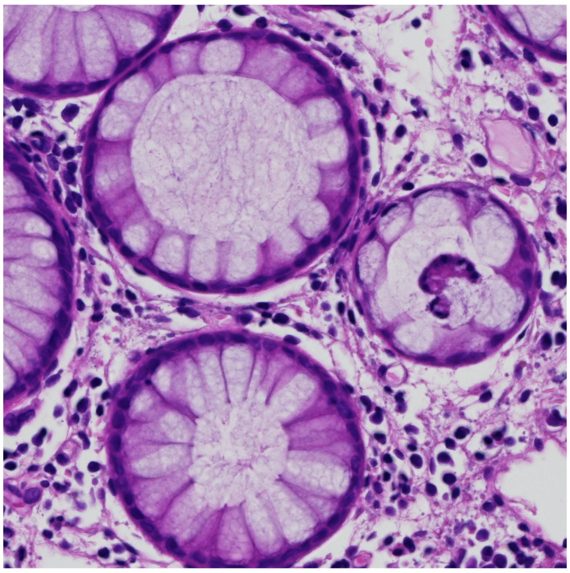

Figure 10. Example 2048 × 2048 pixel tile extracted from the centre of a whole slide image (WSI) using `pylibCZIrw`. This patch, from case SR1482_T232, illustrates the fine detail captured at 40X (0.1112μm per pixel) resolution. Extraction of patches, as demonstrated here, is an essential step in SOTA preprocessing pipelines.

Table 4. Breakdown of SR386 SurGen Colorectal Cohort data distribution for train, validate, and test sets. This stratification may act as an effective starting point for future analysis. Each patient has precisely one associated whole slide image. This breakdown was stratified by age, sex, MSI/MMR, RAS (KRAS or NRAS), and BRAF mutation.

Category	Total (SR386)	Train	Validate	Test
Origin	Scotland	Scotland	Scotland	Scotland
WSI file format	CZI	CZI	CZI	CZI
Magnification	×40	×40	×40	×40
Microns per pixel (pixel width)	0.1112μm	0.1112μm	0.1112μm	0.1112μm
Number of patients	423 (100%)	255 (60%)	84 (20%)	84 (20%)
Mean age at diagnosis (std. dev.)	67.89 (±11.97)	67.98 (±12.12)	67.71 (±11.40)	67.80 (±12.20)
Male, n (%)	228 (54%)	138 (54.1%)	46 (54.7%)	44 (52.3%)
Female, n (%)	195 (46.0%)	117 (45.8%)	38 (45.2%)	40 (47.6%)
MSS/pMMR, n (%)	391 (92%)	235 (92%)	78 (93%)	78 (93%)
MSI/dMMR, n (%)	32 (8%)	20 (8%)	6 (7%)	6 (7%)
Five year survival (true), n (%)	159 (38%)	100 (39%)	30 (36%)	29 (35%)
Five year survival (false), n (%)	264 (62%)	155 (61%)	54 (64%)	55 (65%)
RAS mutation, n (%)	158 (37%)	97 (38%)	31 (37%)	30 (36%)
RAS wild type, n (%)	265 (63%)	158 (62%)	53 (63%)	54 (64%)
BRAF mutation, n (%)	47 (11.1%)	29 (11.4%)	9 (10.7%)	9 (10.7%)
BRAF wild type, n (%)	375 (88.6%)	225 (88.2%)	75 (89.2%)	75 (89.2%)
BRAF fail, n (%)	1 (0.2%)	1 (0.4%)	0 (0%)	0 (0%)

The dataset's versatility allows it to be used in multiple ways:

- Researchers may choose to utilise the SR386 or SR1482 subsets independently, depending on their specific research questions. For instance, studies focusing on primary tumour characteristics and survival can benefit from the SR386 cohort's valuable genetic and survival data.
- Alternatively, the entire SurGen dataset can be employed collectively as a larger cohort for tasks such as staging or genetic slide-level classification, benefiting from the increased sample size and additional diversity from metastatic tumour sites.
- SurGen also holds significant potential as an external validation set for existing studies and algorithms. External validation is essential for assessing the generalisability of predictive models, and the dataset's comprehensive annotations make it particularly suitable for this purpose [49].

To support systematic benchmarking and methodological comparisons, we provide example stratified data-splits for the SR386 subset (see Table 4), as well as for the SR1482 subset and the combined SurGen dataset. Although detailed stratifications are only presented here for SR386, equivalent splits for the full SurGen dataset and the SR1482 subset are available in the accompanying GitHub repository. Each split is stratified to ensure balanced distributions of key variables such as genetic mutations, MMR/MSI status, and survival metrics. These data partitions establish a standardised, transparent framework for evaluating model performance and reproducibility when utilising the SurGen dataset.

An example of the dataset's utility is demonstrated in a study that explored the feasibility of digital pathology foundation models on the SR386 cohort. Using the UNI model [46], which was benchmarked against various other pathology-pretrained foundation models and an ImageNet-pretrained ResNet-50 [50], this work achieved a test AUROC of 0.7136 for slide-level classification of MMR status [51]. This underscores the dataset's potential in facilitating advanced machine learning applications.

Analyses

To further demonstrate the utility of the SurGen dataset, we conducted an experiment combining the SR386 and SR1482 cohorts to predict MMR status using a machine learning model. We utilised the existing training, validation, and test splits from each cohort and merged them to form unified training, validation, and test sets. This approach ensured that the combined SurGen dataset adhered to the 60:20:20 ratio for training, validation, and testing, respectively, while maintaining a balanced representation of mutation statuses across each split. By leveraging the predefined splits from both cohorts, we eliminated the need to generate a separate third split. The splits used in this experiment are provided in CSV format to ensure reproducibility.

Feature Extraction

A range of pre-trained foundation models have been developed for histopathological image analysis, each leveraging diverse self-supervised learning techniques and trained on extensive collections of WSIs. These models have demonstrated considerable success in capturing nuanced histopathological features [52, 53, 54, 55, 56, 57, 46, 58, 59, 60, 61, 62, 63, 64, 65, 66, 67, 68, 69, 70, 71, 72, 73].

For this study, we employed the UNI foundation model [46] for feature extraction from WSIs. UNI was selected due to its robust performance in representing histopathological features relevant to microsatellite instability (MMR status) within the SR386 cohort [51]. The model is a self-supervised vision encoder trained on over 100,000 H&E-stained WSIs across a wide variety of tumour sites, thereby providing a comprehensive representation of tissue morphology.

Feature extraction was performed on non-overlapping 224x224 tissue patches at a scale of 1.0 microns per pixel (MPP), yielding a 1024-dimensional embedding for each patch. Background subtraction was applied as illustrated in Figure 11. The entire process of patch extraction and feature embedding required 110.55 hours, utilising a single NVIDIA V100 32GB GPU. For convenience and reproducibility, these embeddings are made available online.

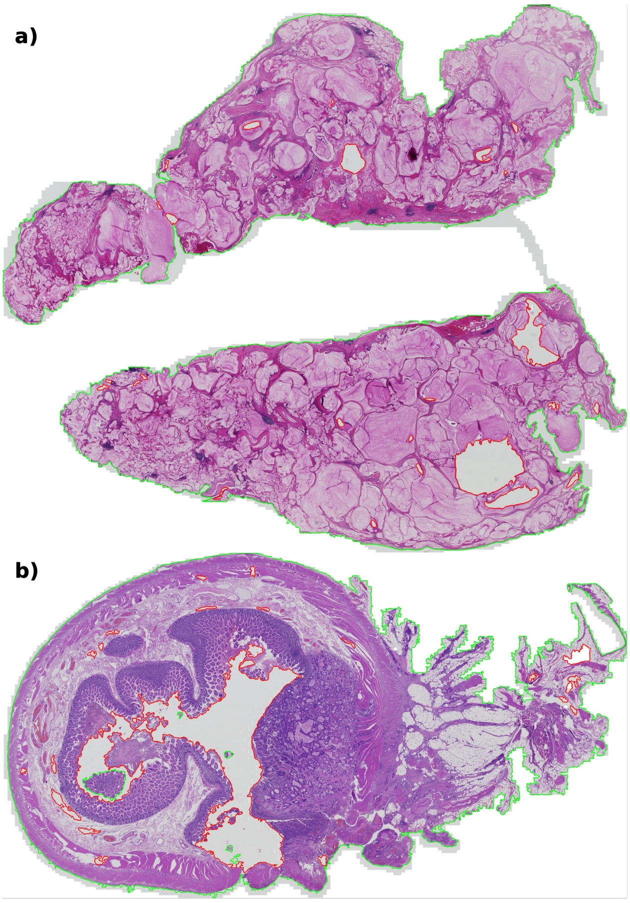

Figure 11. Background subtraction from a) case SR148_T230, peritoneal biopsy and b) case SR148_T412, small bowel resection. Tissue area is outlined in green with holes and background delineated in red.

Model Training and Evaluation

A Transformer [74] based classifier was trained using the extracted UNI patch embeddings. Details of the model parameters are provided in Table 5. Performance was evaluated primarily using the Area Under the Receiver Operating Characteristic curve (AUROC) metric. Training was conducted on a single NVIDIA V100 32GB GPU, completing in 3 hours, 2 minutes, and 36 seconds. The progression of the training and validation AUROC, as well as the loss over 200 epochs, is shown in Figure 12. This figure highlights key performance metrics, including the highest validation AUROC and the lowest validation loss. Preliminary results indicate a validation AUROC of 0.9297 and a test AUROC of 0.8273 (see Figure 13 for test AUROC curve). These results demonstrate the model's potential for accurately predicting MMR status from WSIs. Future work could focus on fine-tuning hyperparameters and exploring the integration of state-of-the-art (SOTA) pretrained feature extractors to further improve model performance.

Model Architecture

The model consists of a feature embedding layer, a transformer encoder, an aggregation layer, and a classification head. The feature extractor used was the UNI model, which produced 1024-dimensional feature vectors for each patch. These were mapped to a 512-dimensional latent space via a fully connected layer and ReLU activation. The transformer encoder consisted of 2 layers, each with 2 attention heads, and a feedforward dimension of 2048. After passing through the transformer encoder, the patch features were mean-pooled to obtain a slide-level feature representation. A final fully connected layer then mapped the pooled feature vector to the number of classes (for multi-class tasks) or to a single output (for binary classification). The full architecture configuration is detailed in table 5.

Training Configuration

The model was trained using patch embeddings extracted from WSIs at 1.0μ/*pixel* per pixel, with patch sizes of 224x224. As the number of patches per WSI varied based on the specimen size, we processed all patches in a single forward pass. The training was conducted on a single NVIDIA V100 32GB GPU, with a batch size of 1 and a learning rate of 1×10^{-4}. The Adam optimiser was used, and binary cross-entropy with logits loss (`BCEWithLogitsLoss`) was applied for binary classification tasks. No class balancing was performed. The model was trained for 200 epochs, and automatic mixed precision (AMP) was enabled to optimise GPU usage. Table 5 provides a summary of the key parameters used in the training process.

Table 5. Summary of model parameters used for MMR/MSI classification.

Parameter	Value
**Task**	MMR/MSI Detection
**Cohort**	SurGen
**Feature Extractor**	*UNI*
**Patch Size**	224x224
**Microns per Pixel (MPP)**	1.0
**Embedding Dimension** (d_{model})	512
**Transformer Encoder Layers** (L)	2
**Attention Heads** (H)	2
**Feedforward Dimension** (d_{ff})	2048
**Activation Function**	ReLU
**Dropout Rate**	0.15
**Layer Norm Epsilon**	1×10^{-5}
**Loss Function**	BCEWithLogitsLoss
**Optimiser**	Adam
**Learning Rate**	1×10^{-4}
**Batch Size**	1
**Epochs**	200
**Automatic Mixed Precision (AMP)**	True
**GPU**	NVIDIA V100 32GB

Experiment Results

The results underscore the strong utility of the SurGen dataset for developing predictive models in computational pathology. Compared with the previous work[51], which achieved a 0.7136 AUROC on the smaller SR386 subset, the higher test AUROC of 0.8273 observed here suggests that SurGen's broader scope and consistently high-quality images may foster more robust model performance. Although additional investigation is necessary to establish whether this improvement stems primarily from the expanded sample size, and greater tumour heterogeneity, these findings emphasise the importance of a large, well-curated dataset for accurate MMR status prediction.

The Transformer-based model demonstrated strong performance in predicting MMR status, achieving an AUROC of 0.9297 on the validation set and 0.8273 on the test set. To illustrate how well the model balances sensitivity and specificity, Figure 14 shows the confusion matrices at four thresholds, optimal (0.0119), 0.25, 0.50, and 0.75, providing a detailed breakdown of the model's classification performance. These matrices help reveal trade-offs between true positives and false positives under different decision criteria

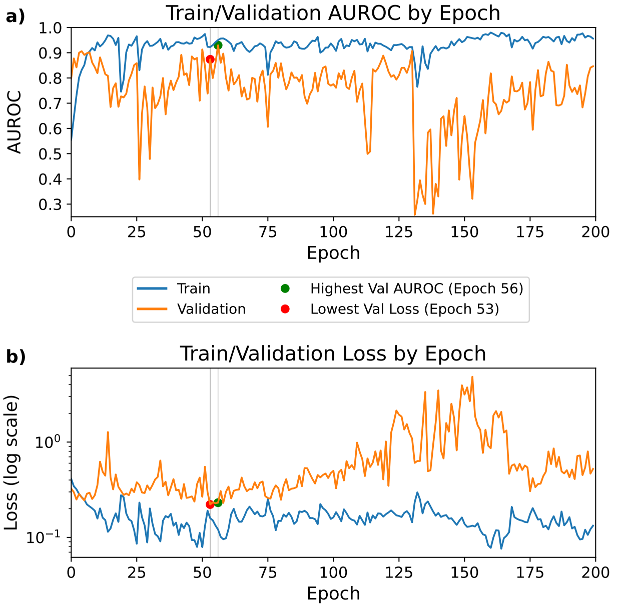

Figure 12. Train/Validation AUROC and Loss by Epoch: a) illustrates the train and validation AUROC progression over 200 epochs, with markers indicating the highest validation AUROC and the epoch with the lowest validation loss. b) shows the train and validation loss on a log scale, highlighting the convergence and divergence trends, with markers indicating key performance metrics such as the lowest validation loss and the epoch with the highest AUROC.

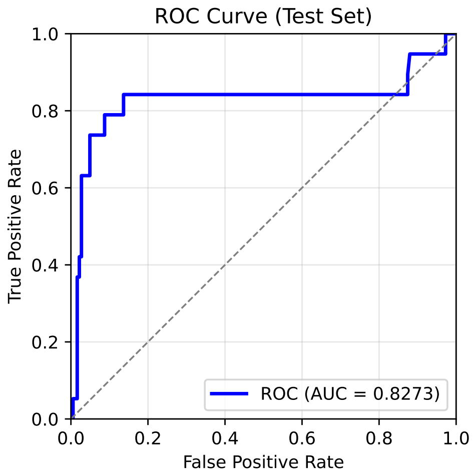

Figure 13. Receiver Operating Characteristic (ROC) curve for the model, showing an AUROC of 0.8273. The curve plots the true positive rate (sensitivity) against the false positive rate (1 - specificity) across various classification thresholds, with an AUROC of 1 representing perfect classification and 0.5 indicating random chance.

and indicate how threshold selection can be tailored for particular clinical aims. For instance, the 0.0119 threshold achieves 95% sensitivity on the validation set, which may be important in early-stage colorectal cancer to minimise the chance of missing diseased cases.

Discussion

In this study, we introduce the SurGen dataset, a comprehensive collection of 1020 H&E stained WSIs from 843 colorectal cancer cases with detailed genetic and clinical annotations. This dataset addresses the critical need for extensive, high-quality datasets in computational pathology to advance cancer diagnosis and treatment. To demonstrate its utility, we developed a machine learning model capable of predicting mismatch repair (MMR) status from the SurGen dataset, achieving a test AUROC of 0.8273 with no hyperparameter tuning. This performance demonstrates a significant improvement over previous efforts which, despite extensive hyperparameter optimisation on the SR386 subset, achieved an AUROC of only 0.7136 [51]. This further motivates the need for large and comprehensive WSI datasets to conduct robust and generalisable computational pathology research. The SurGen dataset directly addresses this need by providing a resource that complements existing datasets with its high-resolution WSIs, extensive annotations, and consistent imaging quality.

Unlike many existing datasets, which often suffer from inconsistent image quality which results in users removing subsets of cases [75, 76, 77, 78, 5], SurGen offers over 1000 consistently high-quality WSIs. This ensures researchers can develop and evaluate models on a dataset that reflects real-world high-quality diagnostic conditions.

The SurGen dataset's extensive annotations and high-quality WSIs make it a valuable resource for developing foundational AI models, enabling transfer learning and domain-specific fine-tuning across a wide range of computational pathology tasks.

By providing a robust foundation for algorithm development, the SurGen dataset supports ongoing efforts to personalise cancer diagnosis and treatment strategies at a global scale.

Potential implications

The SurGen dataset has the potential to impact various areas of cancer research and computational pathology.

In computational pathology, the dataset could serve as a valuable resource for developing and evaluating machine learning algorithms. The diversity of tumour sites and genetic annotations could help in creating more generalisable and robust models. One potential avenue for future research might be to integrate and compare tools such as GrandQC[79] and others [75, 80, 81] which can aid in performing quality control analysis, and in some instances, precise tissue segmentation. Additional research could be developed with the aim of exploring the clinical tabular data with respect to tumour staging, genetic mutation, and survival analysis. Further work could aim to integrate all of these aspects on top of a computer vision model.

While the dataset originates from a single geographical region, it offers an opportunity to study population-specific cancer characteristics. Comparing SurGen with datasets from other regions may help identify global cancer disparities and inform international research. SurGen, in combination with other international datasets, may offer a broad and comprehensive resource that enhances the generalisability of computational models across diverse populations. This integration can facilitate the development of more robust diagnostic tools that are effective in varied clinical settings, ultimately contributing to a more unified and global approach to cancer diagnosis and treatment. Additionally, leveraging SurGen alongside other datasets can support large-scale studies, enabling researchers to validate findings across different cohorts and improve the reliability of predictive models. Such efforts can drive advancements in personalised medicine, ensuring that computational pathology solutions are both accurate and universally applicable.

The SurGen dataset has already been adopted in an independent large-scale benchmark by [82]. In that study a survival prediction pipeline was trained and evaluated across seven public cohorts that span multiple tumour types. The resulting top CoXNet [83] and Supervised C-indices are reproduced in Table 6. SurGen ranks in the upper half of all cohorts and outperforms several CPTAC datasets of comparable size, thereby providing external evidence of its prognostic signal.

Ultimately, the SurGen dataset has the potential to accelerate innovations in cancer diagnostics, enhance treatment personali-

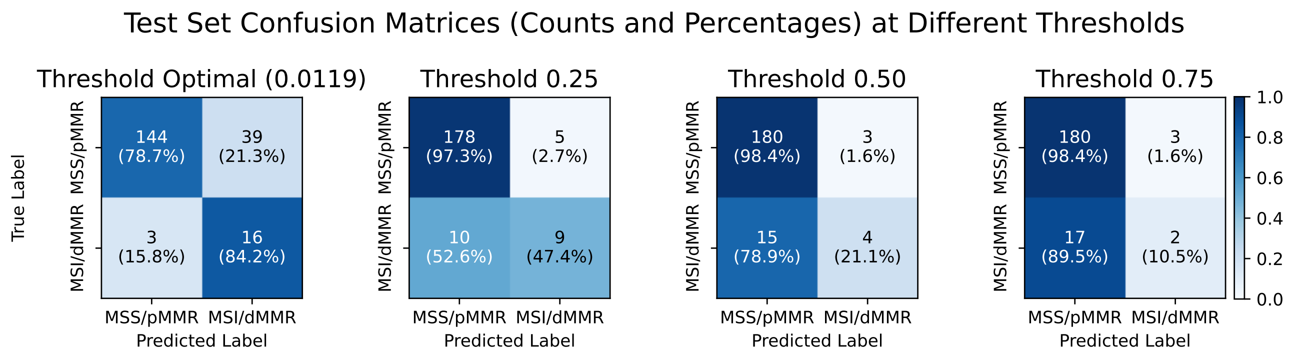

Figure 14. Confusion matrices for mismatch repair (MMR) status prediction at various classification thresholds on the test set. The confusion matrices show the classification results for mismatch repair (MMR) status prediction across four different decision thresholds (0.0119, 0.25, 0.50, and 0.75). Threshold 0.0119 represents the point at which 95% sensitivity on validation set is reached. Each matrix shows the number and percentage of correct and incorrect predictions for the microsatellite-stable/proficient MMR (MSS/pMMR) and microsatellite-instable/deficient MMR (MSI/dMMR) classes.

Table 6. Comparison of Survival Prediction Performance Across Datasets. Results are reported as overall survival C-index (mean ± SE) over 5-fold cross-validation. Data is derived and collated from [82].

Dataset	Patients	CoxNet	Supervised
BOEHMK [84]	183	0.541 ± 0.013	0.575 ± 0.049
**SURGEN** (ours)	144	0.638 ± 0.014	0.632 ± 0.022
CPTAC-LUAD [85]	105	0.614 ± 0.032	0.576 ± 0.046
CPTAC-HNSC [85]	102	0.631 ± 0.076	0.514 ± 0.011
CPTAC-PDAC [85]	97	0.616 ± 0.031	0.611 ± 0.042
CPTAC-CCRCC [85]	94	0.675 ± 0.063	0.693 ± 0.043
MBC [86, 87]	75	0.550 ± 0.027	0.608 ± 0.030

sation, and contribute to reducing the global burden of colorectal cancer.

Availability of Source Code and Requirements

Source code for data-processing and stratification, background subtraction, feature extraction, model training, and evaluation is available via https://github.com/CraigMyles/SurGen-Dataset.

- Project name: SurGen-Dataset
- Project page: https://github.com/CraigMyles/SurGen-Dataset
- Operating system: Ubuntu 20.04 LTS
- Programming language: Python
- Other requirements: Pytorch, pylibCZIrw, pandas, NumPy
- swh:1:snp:39dc17fe24087df9ebae119d77d17398aa1ee25a [88]

Data Availability

The dataset supporting this article is available in the European Molecular Biology Laboratory European Bioinformatics Institute (EMBL-EBI) BioImage Archive repository [89]; available via the following link https://doi.org/10.6019/S-BIAD1285 [90].

Patch embeddings generated during the preprocessing stages using the UNI foundation model have also been made available to reduce the barrier for entry to researchers wishing to utilise this dataset; available via the following link https://doi.org/10.5281/zenodo.14047723 [91].

Dome-ML (Data, Optimization, Model and Evaluation in Machine Learning) annotations are available via the DOME registry under accession vuknweu17e [92]. Supporting data is available via the GigaScience database GigaDB [93].

Compute Resource

In accordance with the recommended minimum documentation for computation time reporting [94], we have detailed the hardware specifications, computation time, and operating system used during the experiments.

Feature extraction from WSIs using the UNI foundation model took 2 days, 10 hours, 12 minutes, and 35 seconds on a system equipped with Dual 20-Core Intel Xeon E5-2698 v4 2.2 GHz and a single NVIDIA Tesla V100 32GB GPU. Model training was completed in 3 hours, 2 minutes, and 36 seconds under the same hardware conditions.

- System: NVIDIA DGX-1
- Operating System: Ubuntu 20.04 LTS
- CPU: Dual 20-Core Intel Xeon E5-2698 v4 2.2 GHz
- GPU: NVIDIA Tesla V100 32GB (Utilised 1 of 8 available)
- RAM: 512 GB DDR4 RAM

Declarations

List of Abbreviations

AUROC: Area Under the Receiver Operating Characteristic; BRAF: v-Raf Murine Sarcoma Viral Oncogene Homolog B; CRC: Colorectal Cancer; CZI: Carl Zeiss Image (file format); dMMR: Deficient Mismatch Repair; FFPE: Formalin-Fixed Paraffin-Embedded; H&E: Hematoxylin and Eosin; IHC: Immunohistochemistry; KRAS: Kirsten Rat Sarcoma Viral Oncogene Homolog; MMR: Mismatch Repair; MSI: Microsatellite Instability; MSS: Microsatellite Stable; NGS: Next Generation Sequencing; NRAS: Neuroblastoma RAS Viral Oncogene Homolog; PCR: Polymerase Chain Reaction; TNM: Tumour, Node, Metastasis; WSI: Whole Slide Image;

Ethical Approval

Ethical approval has been granted by University of St Andrews School of Computer Science Ethics Committee; approval code CS16553. Additionally, Lothian NRS BioResource RTB approval (REC ref – 20/ES/0061 & 13/ES/0126) has been granted.

Consent for Publication

Not applicable. This manuscript does not contain any individual person's data in a form that would require explicit consent for publication. Comprehensive efforts have been made to ensure patient anonymity. Identifiable information, such as dates of diagnosis,

treatment details, and other specifics that could link specimens back to individual patients, have been removed. Furthermore, the dataset has undergone rigorous deidentification processes to aid the prevention re-identification.

Competing Interests

The authors declare that they have no competing interests.

Funding

CM is supported by NHS Lothian. The authors would like to thank NHS Lothian for providing tissue specimens. This work is supported in part by the Industrial Centre for AI Research in Digital Diagnostics (iCAIRD) which is funded by Innovate UK on behalf of UK Research and Innovation (UKRI) (project number 104690).

Author's Contributions

C.M. (C. Myles) led the methodology, investigation, software development, analysis, data curation, and manuscript writing. I.H.U. performed key laboratory work and data acquisition, and contributed to manuscript editing. C.M. (C. Marshall) contributed to data acquisition, governance, and editing. D.H.-B. provided expertise in computational methods, project design, conceptualisation, and manuscript editing. D.J.H. contributed in conceptualisation, clinical insight, and manuscript editing.

Acknowledgements

The authors would like to thank NHS Lothian for supporting this research and NHS Lothian Biorepository for providing tissue specimens. Special thanks to The Harrison Lab team for their dedicated work in slide processing, digitisation, and genetic and biomarker testing. We also acknowledge the MedTech team in the School of Computer Science at the University of St Andrews for their valuable feedback and support throughout this project.

References

1. Sung H, Ferlay J, Siegel RL, Laversanne M, Soerjomataram I, Jemal A, et al. Global cancer statistics 2020: GLOBOCAN estimates of incidence and mortality worldwide for 36 cancers in 185 countries. CA: a cancer journal for clinicians 2021;71(3):209–249.
2. Bray F, Laversanne M, Sung H, Ferlay J, Siegel RL, Soerjomataram I, et al. Global cancer statistics 2022: GLOBOCAN estimates of incidence and mortality worldwide for 36 cancers in 185 countries. CA: a cancer journal for clinicians 2024;74(3):229–263.
3. Bera K, Schalper KA, Rimm DL, Velcheti V, Madabhushi A. Artificial intelligence in digital pathology—new tools for diagnosis and precision oncology. Nature reviews Clinical oncology 2019;16(11):703–715.
4. Niazi MKK, Parwani AV, Gurcan MN. Digital pathology and artificial intelligence. The lancet oncology 2019;20(5):e253–e261.
5. Abels E, Pantanowitz L, Aeffner F, Zarella MD, Van der Laak J, Bui MM, et al. Computational pathology definitions, best practices, and recommendations for regulatory guidance: a white paper from the Digital Pathology Association. The Journal of pathology 2019;249(3):286–294.
6. Litjens G, Bandi P, Ehteshami Bejnordi B, Geessink O, Balkenhol M, Bult P, et al. 1399 H&E-stained sentinel lymph node sections of breast cancer patients: the CAMELYON dataset. GigaScience 2018;7(6):giy065.
7. Spanhol FA, Oliveira LS, Petitjean C, Heutte L. A dataset for breast cancer histopathological image classification. Ieee transactions on biomedical engineering 2015;63(7):1455–1462.
8. Consortium NCICPTA, et al. The Clinical Proteomic Tumor Analysis Consortium Breast Invasive Carcinoma Collection (CPTAC-BRCA). The Cancer Imaging Archive 2020;.
9. Da Q, Huang X, Li Z, Zuo Y, Zhang C, Liu J, et al. DigestPath: A benchmark dataset with challenge review for the pathological detection and segmentation of digestive-system. Medical Image Analysis 2022;80:102485.
10. Kim K, Lee K, Cho S, Kang DU, Park S, Kang Y, et al. PAIP 2020: Microsatellite instability prediction in colorectal cancer. Medical Image Analysis 2023;89:102886.
11. Consortium NCICPTA, et al. The Clinical Proteomic Tumor Analysis Consortium Lung Adenocarcinoma Collection (CPTAC-LUAD). The Cancer Imaging Archive 2018;.
12. Ciardiello F, Ciardiello D, Martini G, Napolitano S, Tabernero J, Cervantes A. Clinical management of metastatic colorectal cancer in the era of precision medicine. CA: a cancer journal for clinicians 2022;72(4):372–401.
13. Weinstein JN, Collisson EA, Mills GB, Shaw KR, Ozenberger BA, Ellrott K, et al. The cancer genome atlas pan-cancer analysis project. Nature genetics 2013;45(10):1113–1120.
14. National Cancer Institute Clinical Proteomic Tumor Analysis Consortium (CPTAC), The Clinical Proteomic Tumor Analysis Consortium Colon Adenocarcinoma Collection (CPTAC-COAD). The Cancer Imaging Archive; 2020. https://doi.org/10.7937/TCIA.YZWQ-ZZ63.
15. Wala J, de Bruijn I, Coy S, Gagne A, Chan S, Chen YA, et al. Integrating spatial profiles and cancer genomics to identify immune-infiltrated mismatch repair proficient colorectal cancers. bioRxiv 2024;p. 2024–09.
16. Zhu CS, Pinsky PF, Kramer BS, Prorok PC, Purdue MP, Berg CD, et al. The prostate, lung, colorectal, and ovarian cancer screening trial and its associated research resource. Journal of the National Cancer Institute 2013;105(22):1684–1693.
17. Pataki BÁ, Olar A, Ribli D, Pesti A, Kontsek E, Gyöngyösi B, et al. HunCRC: annotated pathological slides to enhance deep learning applications in colorectal cancer screening. Scientific Data 2022;9(1):370.
18. Ogino S, Kawasaki T, Kirkner GJ, Kraft P, Loda M, Fuchs CS. Evaluation of markers for CpG island methylator phenotype (CIMP) in colorectal cancer by a large population-based sample. The Journal of molecular diagnostics 2007;9(3):305–314.
19. Mirzapoor Abbasabadi Z, Hamedi Asl D, Rahmani B, Shahbadori R, Karami S, Peymani A, et al. KRAS, NRAS, BRAF, and PIK3CA mutation rates, clinicopathological association, and their prognostic value in Iranian colorectal cancer patients. Journal of clinical laboratory analysis 2023;37(5):e24868.
20. Guo TA, Wu YC, Tan C, Jin YT, Sheng WQ, Cai SJ, et al. Clinicopathologic features and prognostic value of KRAS, NRAS and BRAF mutations and DNA mismatch repair status: a single-center retrospective study of 1,834 Chinese patients with stage I–IV colorectal cancer. International journal of cancer 2019;145(6):1625–1634.
21. De Roock W, Claes B, Bernasconi D, De Schutter J, Biesmans B, Fountzilas G, et al. Effects of KRAS, BRAF, NRAS, and PIK3CA mutations on the efficacy of cetuximab plus chemotherapy in chemotherapy-refractory metastatic colorectal cancer: a retrospective consortium analysis. The lancet oncology 2010;11(8):753–762.
22. Sclafani F, Wilson SH, Cunningham D, Gonzalez De Castro D, Kalaitzaki E, Begum R, et al. Analysis of KRAS, NRAS, BRAF, PIK3CA and TP53 mutations in a large prospective series of locally advanced rectal cancer patients. International Journal of Cancer 2020;146(1):94–102.

23. Burotto M, Chiou VL, Lee JM, Kohn EC. The MAPK pathway across different malignancies: a new perspective. Cancer 2014;120(22):3446–3456.
24. McCain J. The MAPK (ERK) pathway: investigational combinations for the treatment of BRAF-mutated metastatic melanoma. Pharmacy and Therapeutics 2013;38(2):96.
25. Li ZN, Zhao L, Yu LF, Wei MJ. BRAF and KRAS mutations in metastatic colorectal cancer: future perspectives for personalized therapy. Gastroenterology report 2020;8(3):192–205.
26. Boland CR, Goel A. Microsatellite instability in colorectal cancer. Gastroenterology 2010;138(6):2073–2087.
27. Vilar E, Gruber SB. Microsatellite instability in colorectal cancer—the stable evidence. Nature reviews Clinical oncology 2010;7(3):153–162.
28. Le DT, Durham JN, Smith KN, Wang H, Bartlett BR, Aulakh LK, et al. Mismatch repair deficiency predicts response of solid tumors to PD-1 blockade. Science 2017;357(6349):409–413.
29. Luchini C, Bibeau F, Ligtenberg M, Singh N, Nottegar A, Bosse T, et al. ESMO recommendations on microsatellite instability testing for immunotherapy in cancer, and its relationship with PD-1/PD-L1 expression and tumour mutational burden: a systematic review-based approach. Annals of Oncology 2019;30(8):1232–1243.
30. Lynch HT, Lynch P, Lanspa S, Snyder C, Lynch J, Boland C. Review of the Lynch syndrome: history, molecular genetics, screening, differential diagnosis, and medicolegal ramifications. Clinical genetics 2009;76(1):1–18.
31. Tiwari AK, Roy HK, Lynch H. Lynch syndrome in the 21st century: clinical perspectives. QJM: An International Journal of Medicine 2016;109(3):151–158.
32. Hampel H, Frankel WL, Martin E, Arnold M, Khanduja K, Kuebler P, et al. Screening for the Lynch syndrome (hereditary nonpolyposis colorectal cancer). New England Journal of Medicine 2005;352(18):1851–1860.
33. Lynch HT, Lynch JF, Lynch PM, Attard T. Hereditary colorectal cancer syndromes: molecular genetics, genetic counseling, diagnosis and management. Familial cancer 2008;7:27–39.
34. Amin MB, Greene FL, Edge SB, Compton CC, Gershenwald JE, Brookland RK, et al. The eighth edition AJCC cancer staging manual: continuing to build a bridge from a population-based to a more "personalized" approach to cancer staging. CA: a cancer journal for clinicians 2017;67(2):93–99.
35. Dukes CE. The classification of cancer of the rectum. The Journal of Pathology and Bacteriology 1932;35(3):323–332.
36. against Cancer Committee on TNM Classification IU. TNM classification of malignant tumours. International Union Against Cancer; 1974.
37. Haq AI, Schneeweiss J, Kalsi V, Arya M. The Dukes staging system: a cornerstone in the clinical management of colorectal cancer. The lancet oncology 2009;10(11):1128.
38. Sobin LH, Gospodarowicz MK, Wittekind C. TNM classification of malignant tumours. John Wiley & Sons; 2011.
39. Holub P, Müller H, Bíl T, Pireddu L, Plass M, Prasser F, et al. Privacy risks of whole-slide image sharing in digital pathology. Nature Communications 2023;14(1):2577.
40. Goode A, Gilbert B, Harkes J, Jukic D, Satyanarayanan M. OpenSlide: A vendor-neutral software foundation for digital pathology. Journal of pathology informatics 2013;4(1):27.
41. ZEISS, pylibczirw: A Python wrapper for libCZI. GitHub; 2024. Commit ID: 264fcb4ab95274e54433a0054d69f07c402582f4. https://github.com/ZEISS/pylibczirw.
42. Linkert M, Rueden CT, Allan C, Burel JM, Moore W, Patterson A, et al. Metadata matters: access to image data in the real world. Journal of Cell Biology 2010;189(5):777–782.
43. Bankhead P, Loughrey MB, Fernández JA, Dombrowski Y, McArt DG, Dunne PD, et al. QuPath: Open source software for digital pathology image analysis. Scientific reports 2017;7(1):1–7.
44. Schindelin J, Arganda-Carreras I, Frise E, Kaynig V, Longair M, Pietzsch T, et al. Fiji: an open-source platform for biological-image analysis. Nature methods 2012;9(7):676–682.
45. Schneider CA, Rasband WS, Eliceiri KW. NIH Image to ImageJ: 25 years of image analysis. Nature methods 2012;9(7):671–675.
46. Chen RJ, Ding T, Lu MY, Williamson DF, Jaume G, Song AH, et al. Towards a general-purpose foundation model for computational pathology. Nature Medicine 2024;30(3):850–862.
47. Oquab M, Darcet T, Moutakanni T, Vo H, Szafraniec M, Khalidov V, et al. Dinov2: Learning robust visual features without supervision. arXiv preprint arXiv:230407193 2023;.
48. Oliveira SP, Neto PC, Fraga J, Montezuma D, Monteiro A, Monteiro J, et al. CAD systems for colorectal cancer from WSI are still not ready for clinical acceptance. Scientific Reports 2021;11(1):14358.
49. Cui M, Zhang DY. Artificial intelligence and computational pathology. Laboratory Investigation 2021;101(4):412–422.
50. He K, Zhang X, Ren S, Sun J. Deep residual learning for image recognition. In: Proceedings of the IEEE conference on computer vision and pattern recognition; 2016. p. 770–778.
51. Myles C, Um IH, Harrison DJ, Harris-Birtill D. Leveraging Foundation Models for Enhanced Detection of Colorectal Cancer Biomarkers in Small Datasets. In: Annual Conference on Medical Image Understanding and Analysis Springer; 2024. p. 329–343.
52. Wang X, Yang S, Zhang J, Wang M, Zhang J, Yang W, et al. Transformer-based unsupervised contrastive learning for histopathological image classification. Medical image analysis 2022;81:102559.
53. Azizi S, Culp L, Freyberg J, Mustafa B, Baur S, Kornblith S, et al. Robust and data-efficient generalization of self-supervised machine learning for diagnostic imaging. Nature Biomedical Engineering 2023;7(6):756–779.
54. Chen RJ, Chen C, Li Y, Chen TY, Trister AD, Krishnan RG, et al. Scaling vision transformers to gigapixel images via hierarchical self-supervised learning. In: Proceedings of the IEEE/CVF Conference on Computer Vision and Pattern Recognition; 2022. p. 16144–16155.
55. Kang M, Song H, Park S, Yoo D, Pereira S. Benchmarking self-supervised learning on diverse pathology datasets. In: Proceedings of the IEEE/CVF Conference on Computer Vision and Pattern Recognition; 2023. p. 3344–3354.
56. Filiot A, Ghermi R, Olivier A, Jacob P, Fidon L, Mac Kain A, et al. Scaling self-supervised learning for histopathology with masked image modeling. medRxiv 2023;p. 2023–07.
57. Lu MY, Chen B, Williamson DF, Chen RJ, Liang I, Ding T, et al. A visual-language foundation model for computational pathology. Nature Medicine 2024;30(3):863–874.
58. Vorontsov E, Bozkurt A, Casson A, Shaikovski G, Zelechowski M, Severson K, et al. A foundation model for clinical-grade computational pathology and rare cancers detection. Nature medicine 2024;p. 1–12.
59. Campanella G, Kwan R, Fluder E, Zeng J, Stock A, Veremis B, et al. Computational Pathology at Health System Scale–Self-Supervised Foundation Models from Three Billion Images. arXiv preprint arXiv:231007033 2023;.
60. Lai J, Ahmed F, Vijay S, Jaroensri T, Loo J, Vyawahare S, et al. Domain-specific optimization and diverse evaluation of self-supervised models for histopathology. arXiv preprint arXiv:231013259 2023;.
61. Hua S, Yan F, Shen T, Ma L, Zhang X. PathoDuet: Foundation models for pathological slide analysis of H&E and IHC stains. Medical Image Analysis 2024;97:103289.
62. Dippel J, Feulner B, Winterhoff T, Milbich T, Tietz S, Schallenberg S, et al. RudolfV: a foundation model by pathologists for pathologists. arXiv preprint arXiv:240104079 2024;.
63. Aben N, de Jong ED, Gatopoulos I, Känzig N, Karasikov M, Lagré A, et al. Towards Large-Scale Training of Pathology Foundation

Models. arXiv preprint arXiv:240415217 2024;.
64. Juyal D, Padigela H, Shah C, Shenker D, Harguindeguy N, Liu Y, et al. PLUTO: Pathology-Universal Transformer. arXiv preprint arXiv:240507905 2024;.
65. Yang Z, Wei T, Liang Y, Yuan X, Gao R, Xia Y, et al. A foundation model for generalizable cancer diagnosis and survival prediction from histopathological images. bioRxiv 2024;p. 2024–05.
66. Xu H, Usuyama N, Bagga J, Zhang S, Rao R, Naumann T, et al. A whole-slide foundation model for digital pathology from real-world data. Nature 2024;p. 1–8.
67. Nechaev D, Pchelnikov A, Ivanova E. Hibou: A Family of Foundational Vision Transformers for Pathology. arXiv preprint arXiv:240605074 2024;.
68. Saillard C, Jenatton R, Llinares-López F, Mariet Z, Cahané D, Durand E, et al., H-optimus-0; 2024. https://github.com/bioptimus/releases/tree/main/models/h-optimus/v0.
69. Xu Y, Wang Y, Zhou F, Ma J, Yang S, Lin H, et al. A multimodal knowledge-enhanced whole-slide pathology foundation model. arXiv preprint arXiv:240715362 2024;.
70. Zimmermann E, Vorontsov E, Viret J, Casson A, Zelechowski M, Shaikovski G, et al. Virchow 2: Scaling Self-Supervised Mixed Magnification Models in Pathology. arXiv preprint arXiv:240800738 2024;.
71. Filiot A, Jacob P, Mac Kain A, Saillard C. Phikon-v2, A large and public feature extractor for biomarker prediction. arXiv preprint arXiv:240909173 2024;.
72. Wang X, Zhao J, Marostica E, Yuan W, Jin J, Zhang J, et al. A pathology foundation model for cancer diagnosis and prognosis prediction. Nature 2024;p. 1–9.
73. Ding T, Wagner SJ, Song AH, Chen RJ, Lu MY, Zhang A, et al. Multimodal Whole Slide Foundation Model for Pathology. arXiv preprint arXiv:241119666 2024;.
74. Vaswani A, Shazeer N, Parmar N, Uszkoreit J, Jones L, Gomez AN, et al. Attention is all you need. Advances in neural information processing systems 2017;30.
75. Haghighat M, Browning L, Sirinukunwattana K, Malacrino S, Khalid Alham N, Colling R, et al. Automated quality assessment of large digitised histology cohorts by artificial intelligence. Scientific Reports 2022;12(1):5002.
76. Jang HJ, Lee A, Kang J, Song IH, Lee SH. Prediction of clinically actionable genetic alterations from colorectal cancer histopathology images using deep learning. World Journal of Gastroenterology 2020;26(40):6207.
77. Lafarge MW, Domingo E, Sirinukunwattana K, Wood R, Samuel L, Murray G, et al. Image-based consensus molecular subtyping in rectal cancer biopsies and response to neoadjuvant chemoradiotherapy. NPJ precision oncology 2024;8(1):89.
78. Xu H, Cha YJ, Clemenceau JR, Choi J, Lee SH, Kang J, et al. Spatial analysis of tumor-infiltrating lymphocytes in histological sections using deep learning techniques predicts survival in colorectal carcinoma. The Journal of Pathology: Clinical Research 2022;8(4):327–339.
79. Weng Z, Seper A, Pryalukhin A, Mairinger F, Wickenhauser C, Bauer M, et al. GrandQC: A comprehensive solution to quality control problem in digital pathology. Nature Communications 2024;15(1):1–12.
80. Janowczyk A, Zuo R, Gilmore H, Feldman M, Madabhushi A. HistoQC: an open-source quality control tool for digital pathology slides. JCO clinical cancer informatics 2019;3:1–7.
81. Patil A, Diwakar H, Sawant J, Kurian NC, Yadav S, Rane S, et al. Efficient quality control of whole slide pathology images with human-in-the-loop training. Journal of Pathology Informatics 2023;14:100306.
82. Vaidya A, Zhang A, Jaume G, Song AH, Ding T, Wagner SJ, et al. Molecular-driven Foundation Model for Oncologic Pathology. arXiv preprint arXiv:250116652 2025;.
83. Pölsterl S. scikit-survival: A Library for Time-to-Event Analysis Built on Top of scikit-learn. Journal of Machine Learning Research 2020;21(212):1–6.
84. Boehm KM, Aherne EA, Ellenson L, Nikolovski I, Alghamdi M, Vázquez-García I, et al. Multimodal data integration using machine learning improves risk stratification of high-grade serous ovarian cancer. Nature cancer 2022;3(6):723–733.
85. Edwards NJ, Oberti M, Thangudu RR, Cai S, McGarvey PB, Jacob S, et al. The CPTAC data portal: a resource for cancer proteomics research. Journal of proteome research 2015;14(6):2707–2713.
86. Bergstrom EN, Abbasi A, Díaz-Gay M, Galland L, Ladoire S, Lippman SM, et al. Deep learning artificial intelligence predicts homologous recombination deficiency and platinum response from histologic slides. Journal of Clinical Oncology 2024;42(30):3550–3560.
87. Galland L, Ballot E, Mananet H, Boidot R, Lecuelle J, Albuisson J, et al. Efficacy of platinum-based chemotherapy in metastatic breast cancer and HRD biomarkers: utility of exome sequencing. NPJ breast cancer 2022;8(1):28.
88. Myles C, SurGen-Dataset (Version 1) [Computer software]; 2025. Software Heritage snapshot. https://archive.softwareheritage.org/swh:1:snp:39dc17fe24087df9ebae119d77d17398aa1ee25a.
89. Hartley M, Kleywegt GJ, Patwardhan A, Sarkans U, Swedlow JR, Brazma A. The BioImage archive–building a home for life-sciences microscopy data. Journal of Molecular Biology 2022;434(11):167505.
90. Myles C, Um IH, Marshall C, Harris-Birtill D, Harrison DJ, SurGen: 1020 H&E-stained Whole Slide Images With Survival and Genetic Markers. EMBL-EBI BioImage Archive; 2024. https://www.ebi.ac.uk/biostudies/bioimages/studies/S-BIAD1285.
91. Myles C, Um IH, Marshall C, Harris-Birtill D, Harrison DJ, Patch-level UNI feature embeddings from colorectal cancer whole slide images (WSIs) in the SurGen dataset. Zenodo; 2024. https://doi.org/10.5281/zenodo.14047723.
92. DOME-ML annotations for "SurGen: 1020 H&E-stained Whole Slide Images With Survival and Genetic Markers" [DOME registry]; 2025. DOME registry accession vuknweu17e. https://registry.dome-ml.org/review/vuknweu17e.
93. Myles C, Um IH, Marshall C, Harris-Birtill D, Harrison DJ, Supporting data for "SurGen: 1020 H&E-stained Whole Slide Images With Survival and Genetic Markers"; 2025. Dataset, GigaScience Database. http://gigadb.org/dataset/102725.
94. Harris-Birtill D, Harris-Birtill R. Understanding computation time: a critical discussion of time as a computational performance metric. In: Time in Variance Brill; 2021.p. 220–248.

Supplementary File S1 – Example Python script

```python
from pylibCZIrw import czi
# Path to the CZI file
path = "./SR1482_40X_HE_T232_01.czi"

# Open the CZI file and read a patch from the center
with czi.open_czi(path) as czidoc:
    bbox = czidoc.total_bounding_box
    x_min, x_max = bbox['X']
    y_min, y_max = bbox['Y']

    patch_size = 2048

    # Calculate the center coordinates
    center_x = (x_min + x_max) // 2
    center_y = (y_min + y_max) // 2

    # Calculate ROI coordinates
    roi_x = center_x - patch_size // 2
    roi_y = center_y - patch_size // 2

    # Read the patch at full resolution
    patch = czidoc.read(
        roi=(roi_x, roi_y, patch_size, patch_size),
        zoom=1.0 # Render at full (40X) resolution
    )
```

Listing 1. Python code demonstrating how to extract a tile from the centre of a WSI using in `Python 3.8.13` and `pylibCZIrw v4.1.3`. This example illustrates how to interact with high-resolution pathology images in CZI format. This method can be easily expanded to tessellate over an entire whole slide image for the purpose of patch-level feature extraction.

Figure

Click here to access/download;Figure;bar-chart-surv-dist.pdf

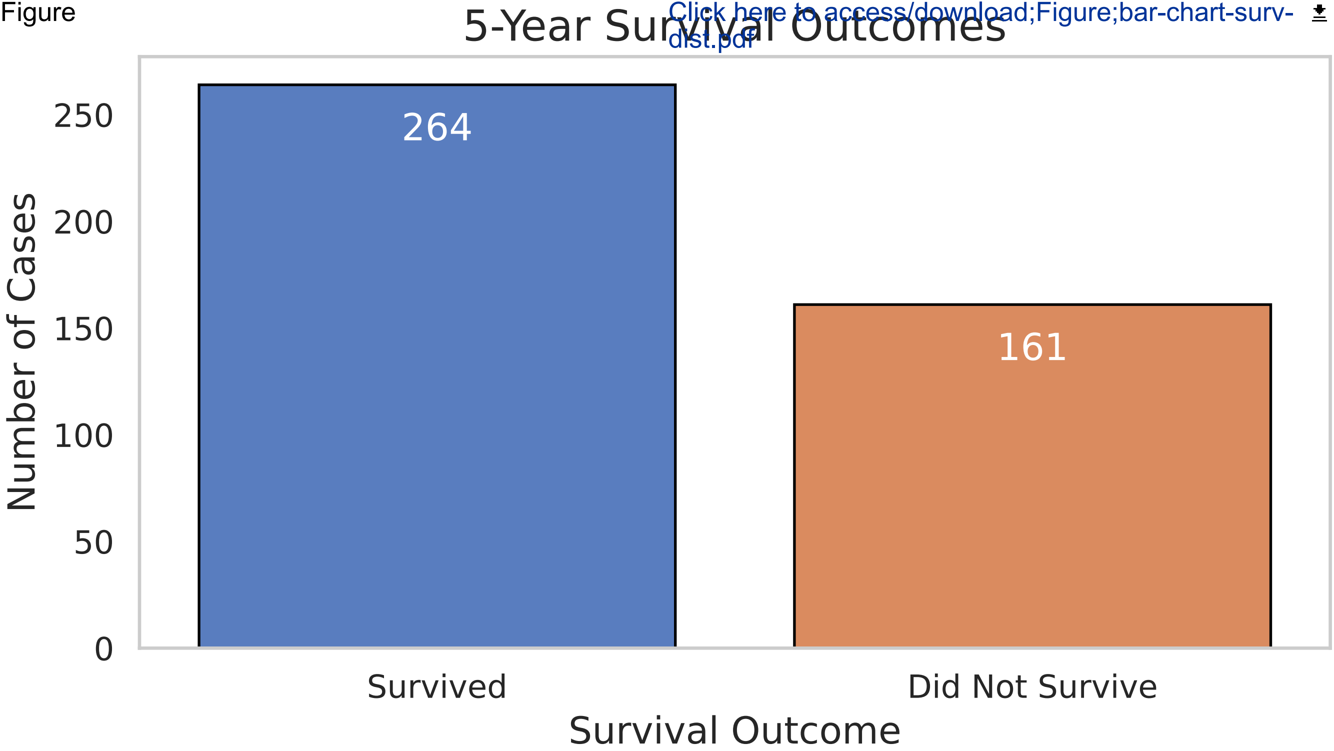

Figure

Click here to access/download;Figure;boxplot-survival-times.pdf

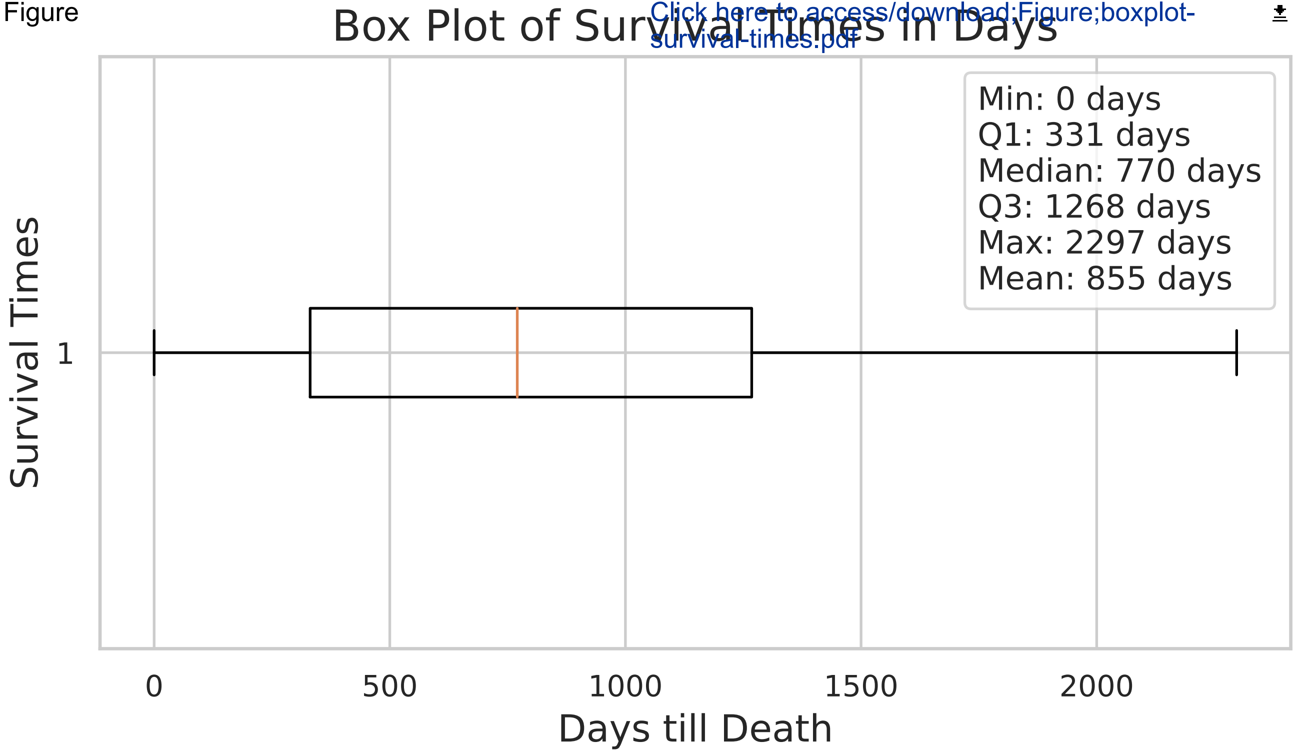

Figure

Click here to access/download;Figure;combined-auroc-loss-by-epoch.pdf

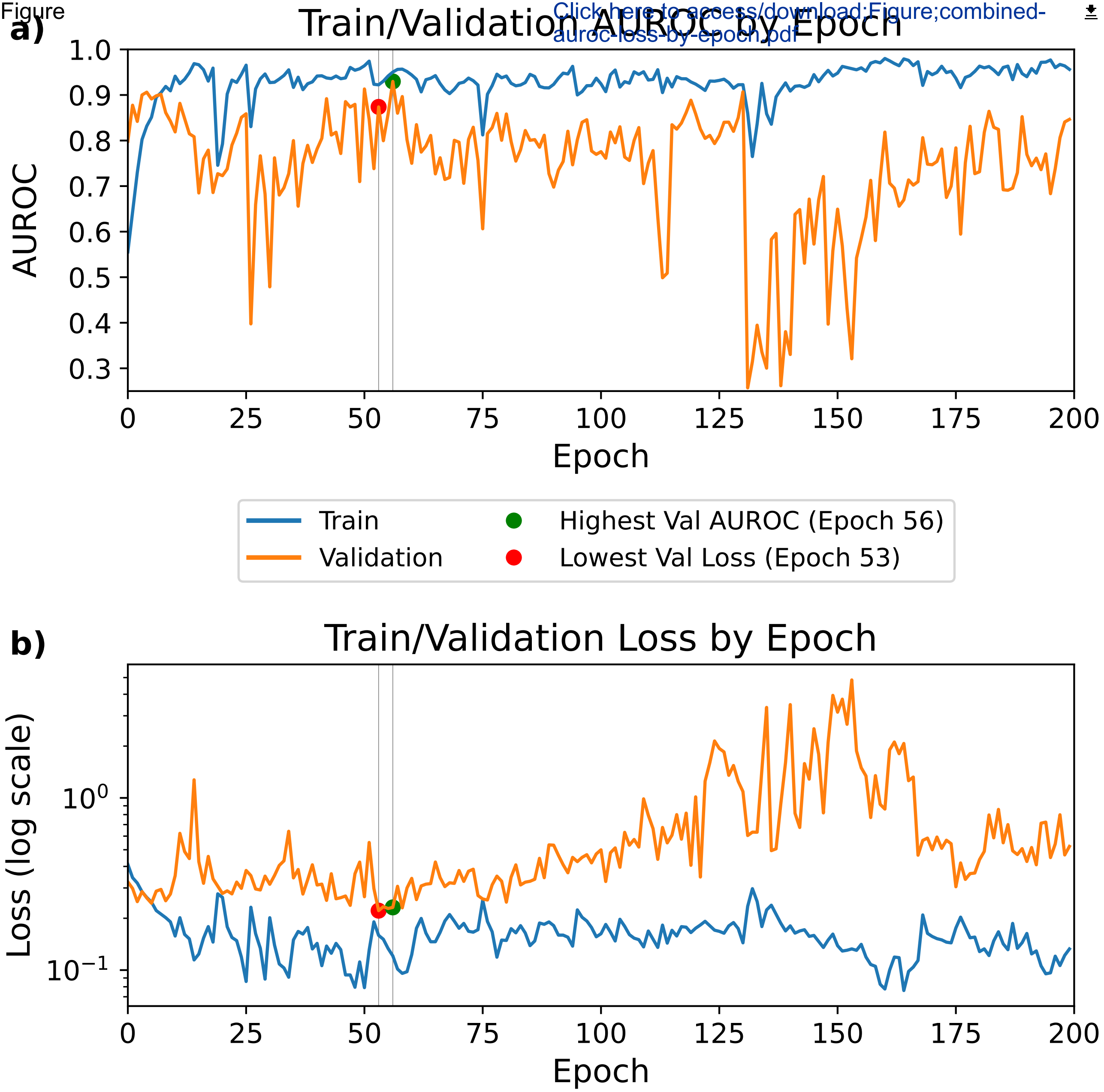

Figure

Click here to access/download;Figure;confusion-matrices.pdf

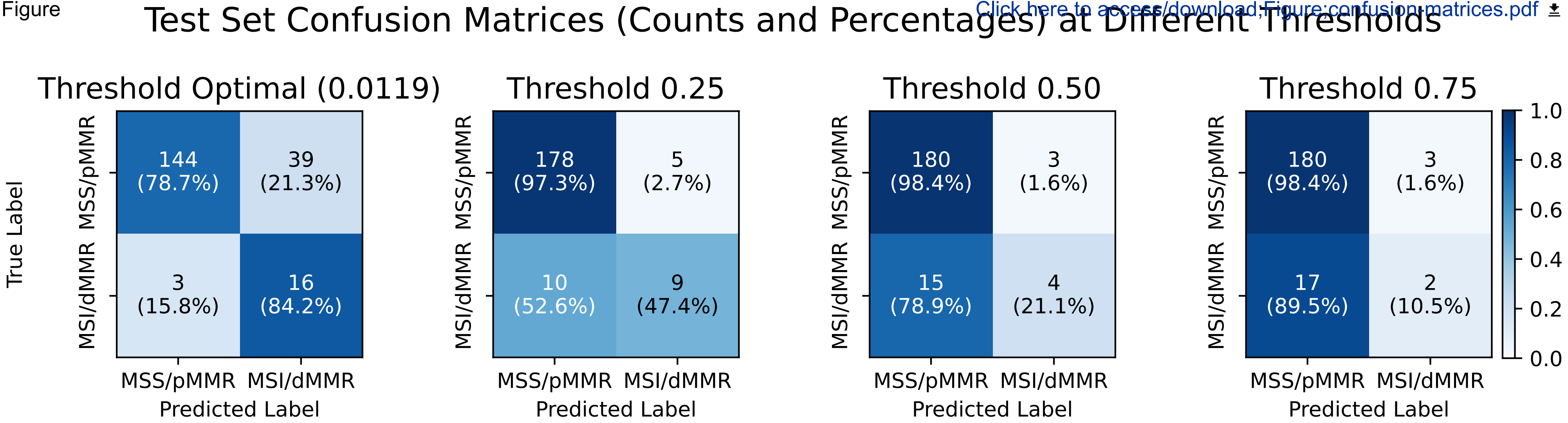

Figure

Click here to access/download;Figure;heatmap-dimensions.pdf

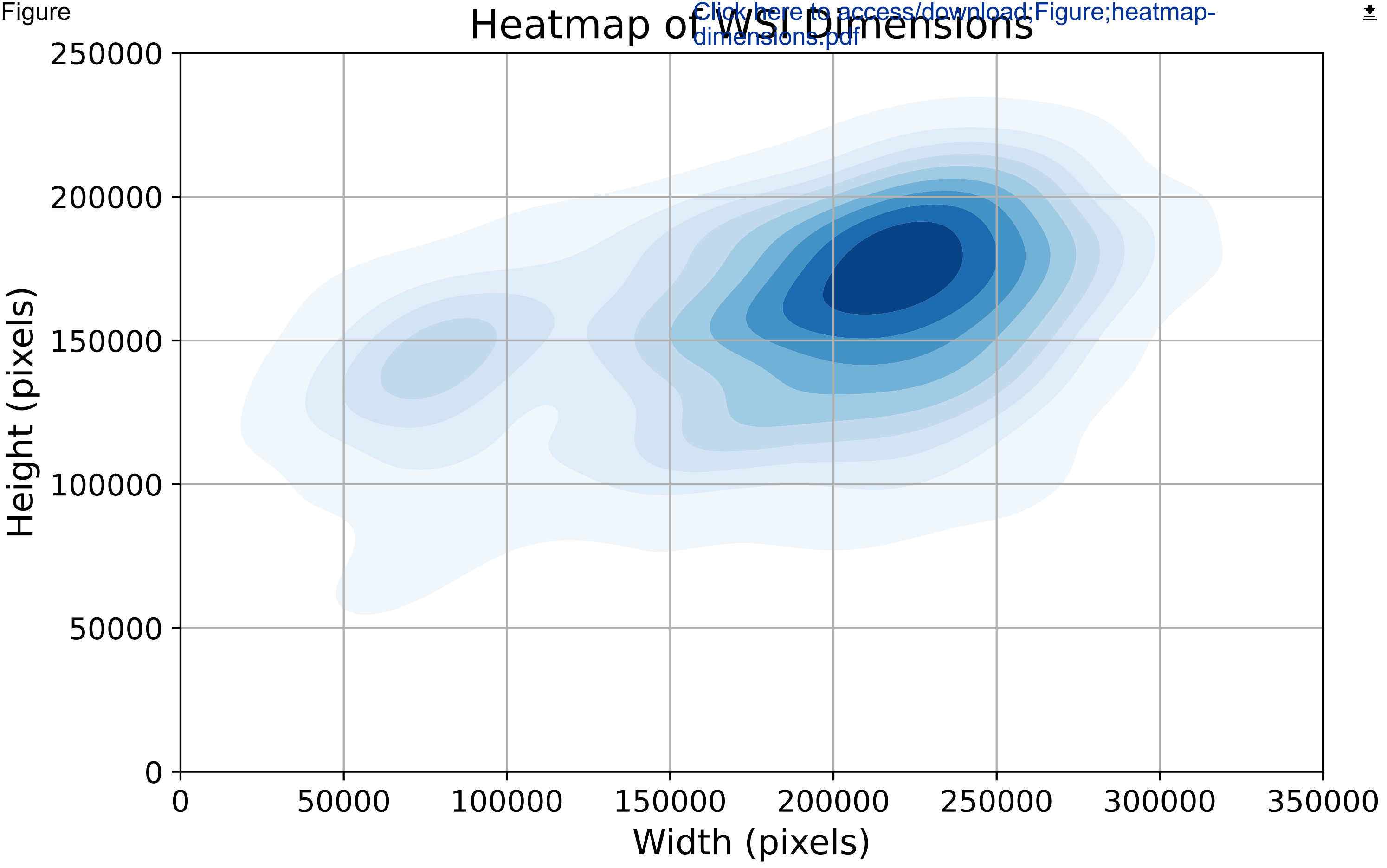

Figure

Click here to access/download;Figure;histogram-resolution.pdf

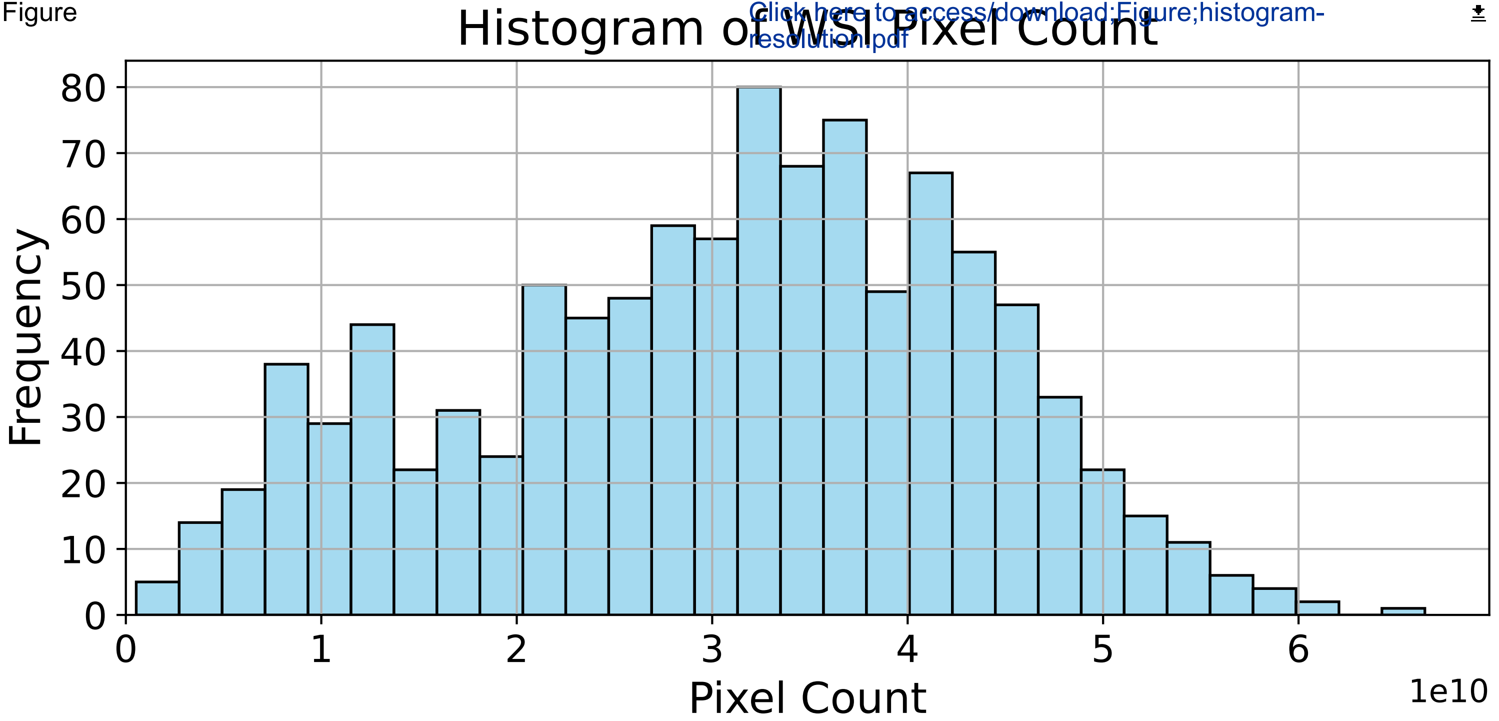

Figure

Click here to access/download;Figure;histogram-survival-times.pdf

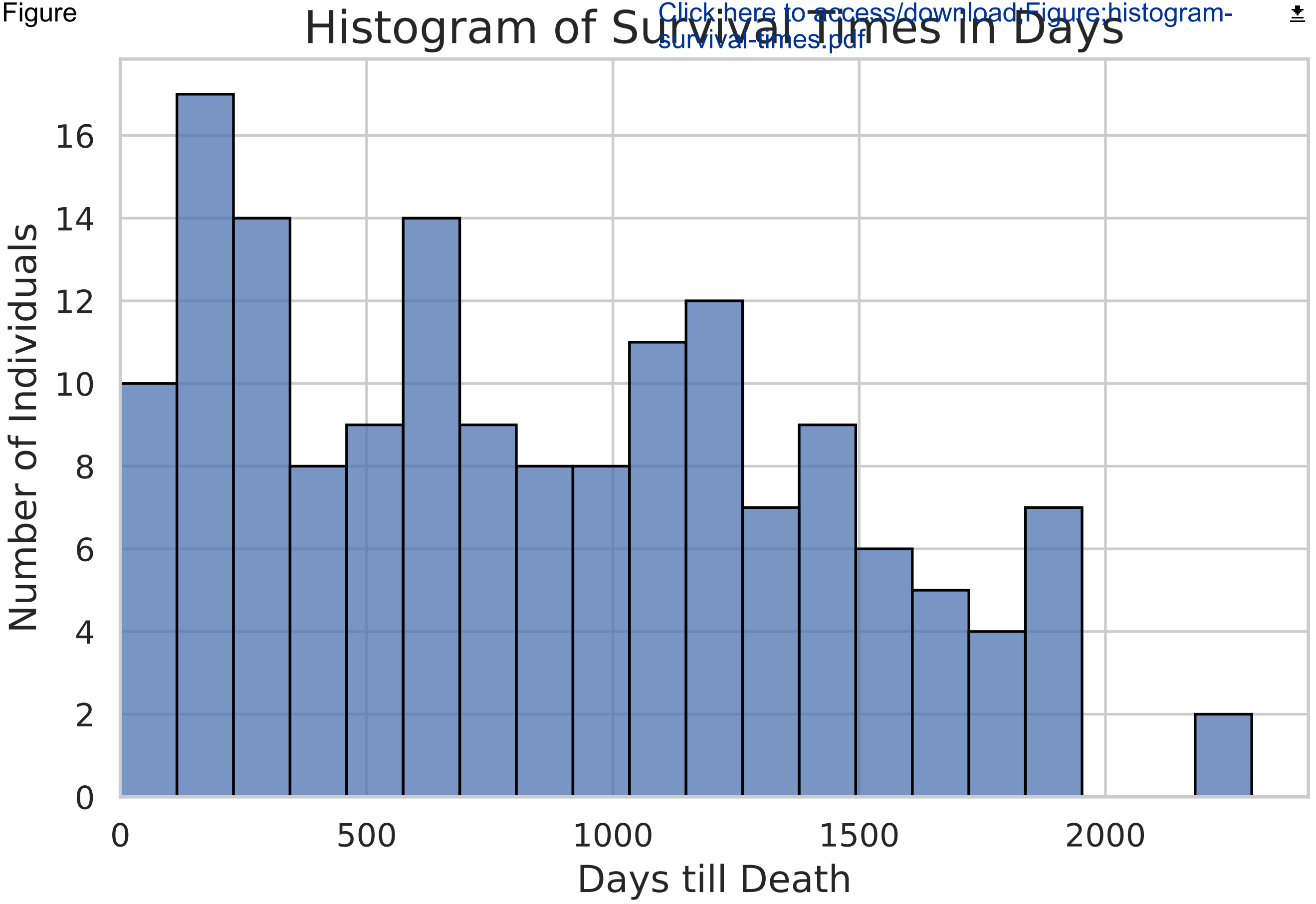

Figure

Click here to access/download;Figure;Kaplan-Meirer-

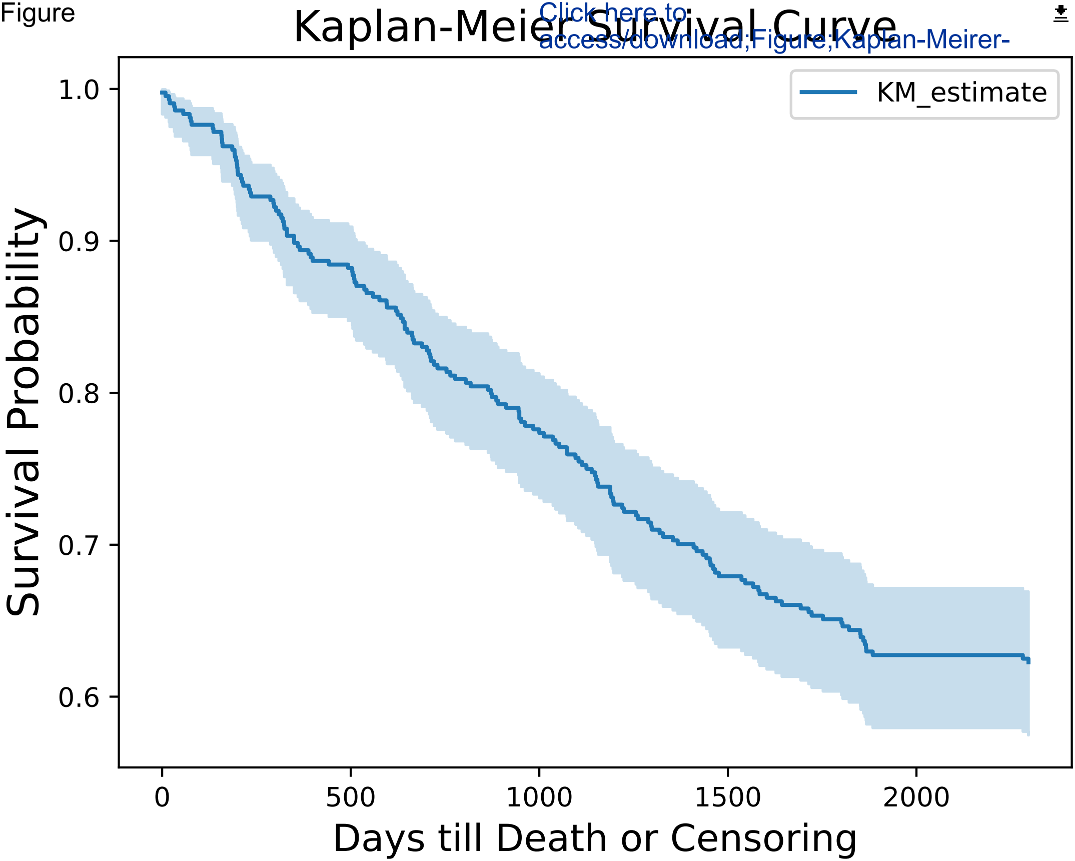

Figure

[Click here to access/download;Figure;mutation-bar-charts.pdf](#)

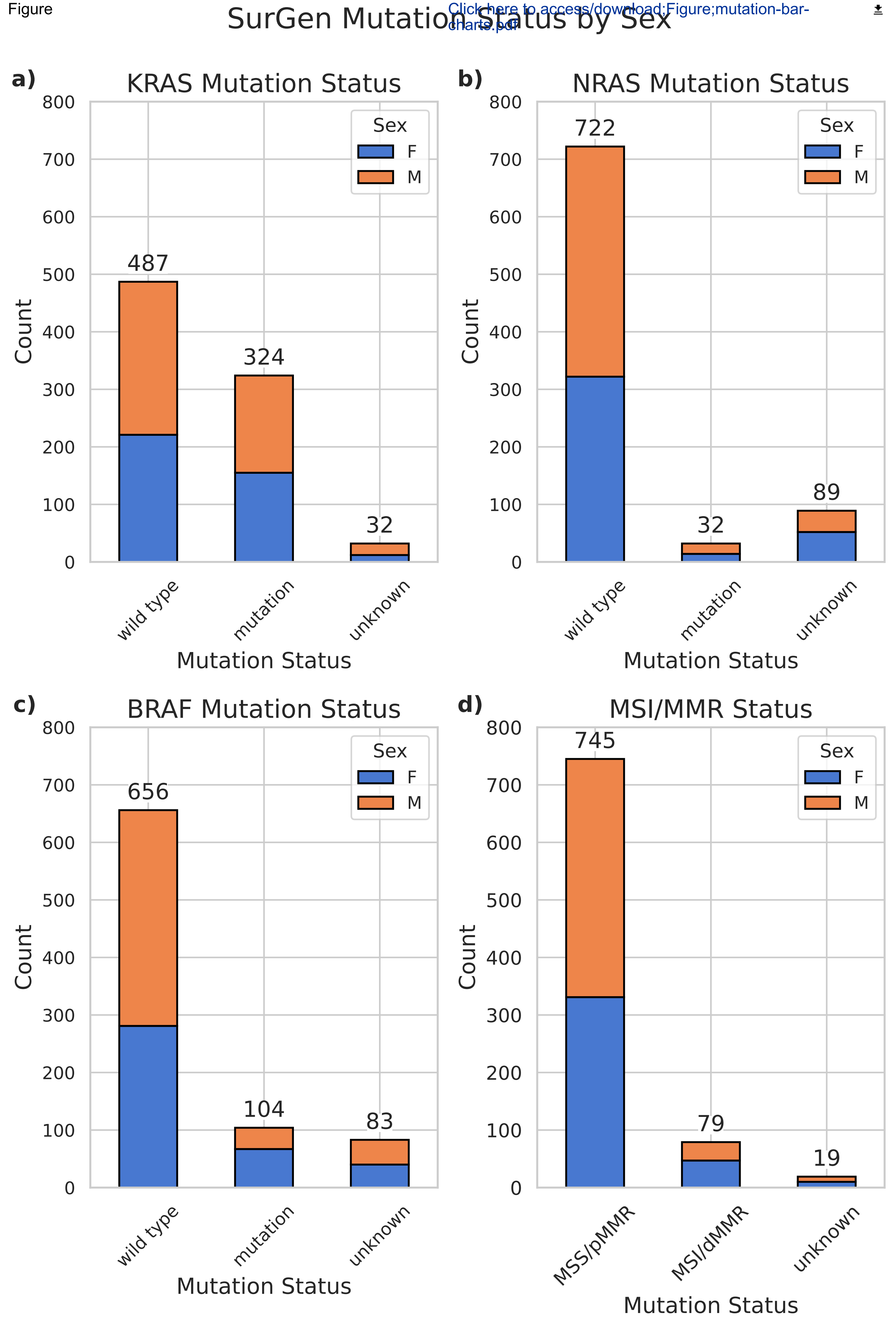

Figure

Click here to access/download;Figure;roc

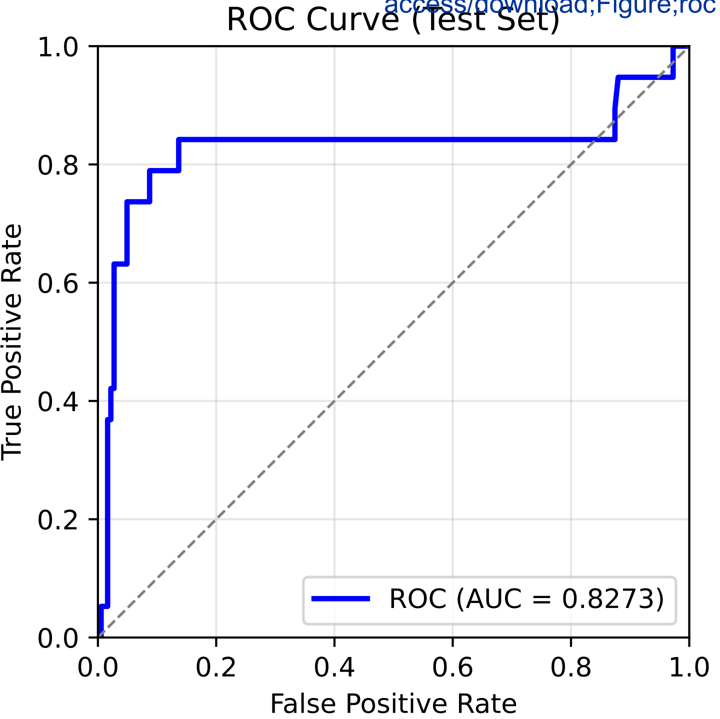

Figure

Click here to access/download;Figure;SurGen-age-dist-bisected-by-sex.pdf

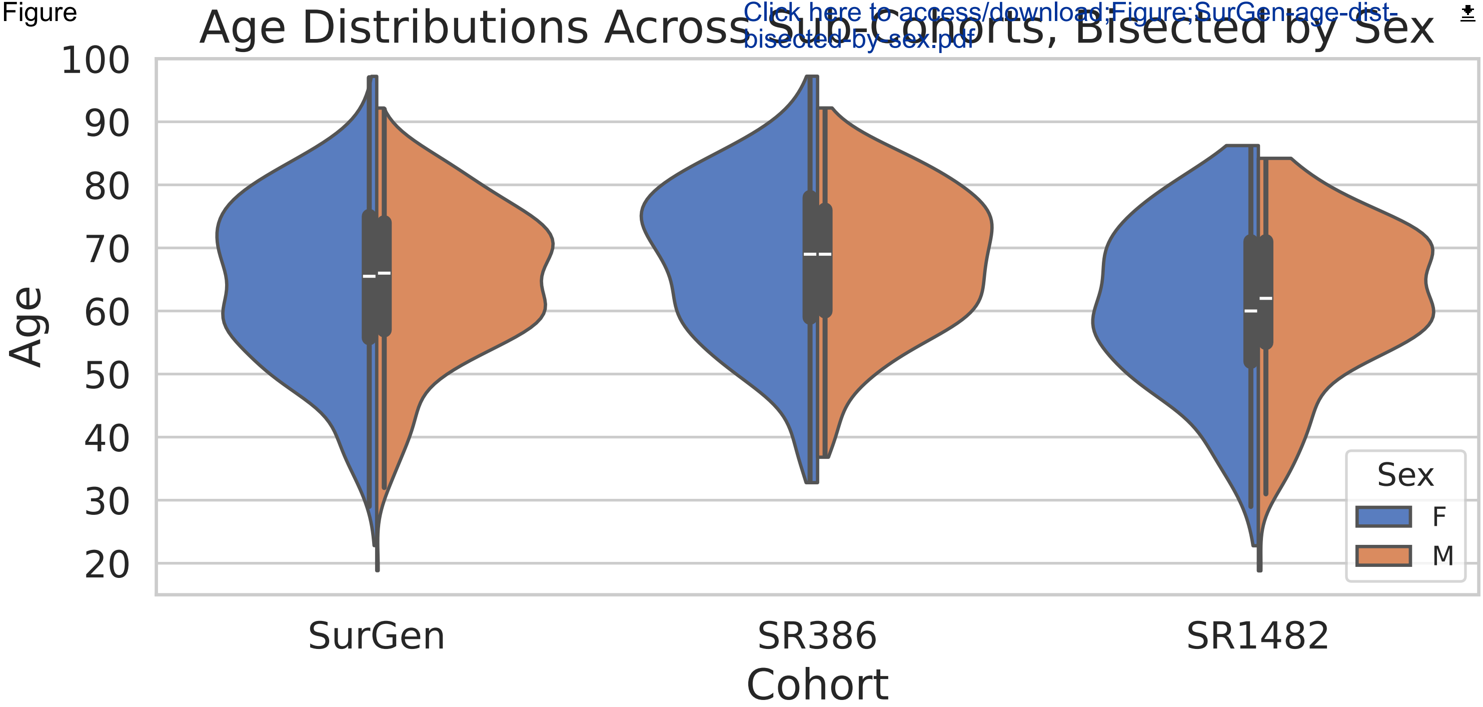

Supplement: giaf086_GIGA-D-24-00594_Revision_2 [file giaf086_giga-d-24-00594_revision_2.pdf]
