# Supplementary material for: SurGen: 1020 H&E-stained whole-slide images with survival and genetic markers
Source: Gigascience. 2025 Oct 8;14:giaf086. doi: 10.1093/gigascience/giaf086 (PMC12569769; doi:10.1093/gigascience/giaf086)
Supplement: giaf086_Authors_Response_To_Reviewer_Comments_Revision_1 [file giaf086_authors_response_to_reviewer_comments_revision_1.pdf]

Revision-round	1
File-type	author-comment
Role	author
Name	Mr Craig Myles
Date-type	author-comment-received
Date	5th May 2025

Author's Response to Reviewer Comments

Manuscript: SurGen: 1020 H&E-stained Whole Slide Images With Survival and Genetic Markers

Manuscript ID: GIGA-D-24-00594

We thank both reviewers for their detailed and constructive comments. Below, we address each point in turn, indicating what changes were made (or clarifications provided) in the manuscript.

Reviewer #1 Comments

> 1. Comment on MPP and magnification
> "MPP is approximately 0.11 µm/px. Authors state this is 400x magnification. However, typical MPP for 400x magnification is 0.25. Either it is digital zoom to 800x or real 800x magnification. This should be clarified by authors."

We confirmed with our scanning equipment specifications (Zeiss Axio Scan.Z1) that the lens is a 40× Plan-Apochromat objective, yielding 0.1112 µm/px natively. This resolution can differ from other scanners (e.g., Philips, Hamamatsu, Leica) where "40×" can in some cases correspond to ~0.25 µm/px. We now explicitly state in the "Tissue Sample Digitisation" subsection that our pixel size is 0.1112 µm/px and label it as such, and clarify that different manufacturers label nominal magnifications differently. Please see the revised text in Section "Data Description" (Tissue Sample Digitisation subsection).

> 2. WSI selection
> "The authors should clarify exactly how the selection of WSIs was done. In the survival subcohort they have 1 WSI/patient. E.g., TCGA cohort also has 1 WSI/patient known to contain significant biases."

Thank you for this suggestion. Each WSI in the SR386 subcohort is digitised from the

single formalin-fixed paraffin-embedded block used for genomic tests. We have now clarified this process in the Data Description (under Tissue Sample Digitisation).

> 3. Acknowledge PLCO dataset
> “Authors should acknowledge open-source PLCO cohort which is similarly large or even larger (but lacks molecular data).”

We have now added a brief mention of the Prostate, Lung, Colorectal and Ovarian (PLCO) dataset in our Background section. Although PLCO is large, it requires an application process and does not readily provide molecular data for CRC, so it is less directly comparable to SurGen. Nonetheless, we have actioned the reviewer’s recommendation to acknowledge its scale. (See Background.)

> 4. GrandQC
> “I highly recommend to run QC tool (e.g., GrandQC) on the dataset and provide tissue detection maps and QC masks (artifacts masked) in the repo along the images to make the dataset fully usable out-of-box.”

We fully agree that advanced automated QC, such as via GrandQC, are excellent tools. In our present release, we relied on manual slide review and were not in a position produce automated QC masks for each slide. To reflect this, we have:

1. Mentioned in the Potential Implications section how tools like GrandQC [Weng et al., 2024] and others (HistoQC, PathProfiler, HistoROI) could be integrated by future users to generate segmentation masks or filter artefacts.

2. Indicated that further providing QC masks is outside the immediate scope for this data note but that this may be an interesting area of investigation should some researchers wish to explore this.

We certainly appreciate the reviewer’s concern about immediate usability and encourage researchers who adopt SurGen to explore these automated QC frameworks. (See Section “Potential implications” in the revised manuscript.)

Reviewer #2 Comments

> 1. Training on external datasets
> “Training on External Datasets:To further validate the dataset's robustness, please consider training the models using publicly available datasets such as TCGA and CPTAC for colorectal cancer (CRC) and comparing their performance with models trained on SurGen. This will help highlight the generalizability and advantages of SurGen over existing datasets.”

We agree that cross-cohort benchmarking is beneficial. While we did not retrain fully on external datasets in this work, we have expanded our Potential implications section. We now cite Vaidya *et al.* (2025), who trained a single foundation-model pipeline on seven public cohorts, including four CPTAC datasets and SurGen, under an identical five-fold cross-validation protocol. Their published C-indices (reproduced in the revised manuscript, Table 6) show that SurGen performs competitively, surpassing CPTAC-PDAC, CPTAC-LUAD and two biopsy collections.

> 2. External validation or core validation
> “External Validation or Core Validation Approach:While the dataset is comprehensive, external validation using an independent cohort would strengthen its impact. If external validation is not feasible, a core validation approach within SurGen (e.g., cross-institutional validation or region-wise stratification) would also be a valuable alternative.”

Thank you for emphasising external validation. SurGen is primarily from a single region. We do outline a standard 60:20:20 train/validate/test approach within SurGen, ensuring we have minimal data leakage and robust performance estimates. We also invite future studies to integrate SurGen with additional cohorts (e.g. TCGA, CPTAC) for cross-institutional testing. To address this reviewer comment, a new table which includes external validation from recent works has been added. The Potential Implications section has been updated accordingly.

> 3. Enhanced comparison with existing datasets
> “Enhanced Comparison with Existing Datasets:The manuscript already provides a comparative table, but an additional quantitative performance comparison of SurGen with other datasets (e.g., TCGA, CPTAC) in terms of model performance would be beneficial.”

We thank the reviewer for their helpful comments. We have altered our revised manuscript to include a comparative table of open-source resources showing an in-depth multi-dataset performance benchmark. As mentioned, we have added references to recent large-scale benchmarks that do incorporate SurGen in addition to other open-datasets, giving readers an avenue to see how SurGen might stack up in cross-dataset performance. See changes reflected in Potential Implications section.

> 4. Supplementary code placement
> “Supplementary Code Placement:Some code is currently included in the main text. Moving it to the supplementary materials would improve the flow of the manuscript while ensuring accessibility for interested readers.”

We have moved the code snippet into Supplementary Materials (Supplementary File S1) and additionally into the GitHub repository, linking them from the main text. We

agree this streamlines the manuscript layout while preserving reproducibility. Thanks for the suggestion.

We hope these revisions address the reviewers' points thoroughly. We appreciate their insights, which have improved our presentation and highlighted additional avenues for using SurGen.

Sincerely,
Craig Myles (on behalf of all authors)
