# Supplementary material for: SurGen: 1020 H&E-stained whole-slide images with survival and genetic markers
Source: Gigascience. 2025 Oct 8;14:giaf086. doi: 10.1093/gigascience/giaf086 (PMC12569769; doi:10.1093/gigascience/giaf086)
Supplement: giaf086_Authors_Response_To_Reviewer_Comments_Revision_3 [file giaf086_authors_response_to_reviewer_comments_revision_3.pdf]

Revision-round	3
File-type	author-comment
Role	author
Name	Mr Craig Myles
Date-type	author-comment-received
Date	30th June 2025

Author's Response to Reviewer Comments

Updates applied.
