# Supplementary material for: SurGen: 1020 H&E-stained whole-slide images with survival and genetic markers
Source: Gigascience. 2025 Oct 8;14:giaf086. doi: 10.1093/gigascience/giaf086 (PMC12569769; doi:10.1093/gigascience/giaf086)
Supplement: giaf086_Reviewer_1_Report_Original_Submission — Prof. Yuri Tolkach -- 2/2/2025 [file giaf086_reviewer_1_report_original_submission.pdf]

Revision-round	0
File-type	reviewer-report
Role	reviewer 1
Name	Prof. Yuri Tolkach
Date-type	reviewer-report-received
Date	2nd February 2025

Reviewer Report

Title: SurGen: 1020 H&E-stained Whole Slide Images with Survival and Genetic Markers

Version: Original Submission **Date: 2/2/2025**

Reviewer name: Prof. Yuri Tolkach

Reviewer Comments to Author:

This is an article describing a new, comparably large dataset for colorectal cancer including digitized histological images, molecular-genetic data, and survival data (for one of two subcohorts). The dataset is already online. I was able to download exemplary files. They are of adequate quality. Authors provide a formal example of training a linear classifier for MSI. I have only few remarks that might improve the presentation of data and usability of the dataset: -MPP is approximately 0.11 µm/px. Authors state this is 400x magnification. However, typical MPP for 400x magnification is 0.25. Either it is digital zoom to 800x or real 800x magnification. This should be clarified by authors. -The authors should clarify exactly how the selection of WSIs was done. In the survival

subcohort they have 1 WSI/patient. E.g., TCGA cohort also has 1 WSI/patient known to contain significant biases. -Authors should acknowledge open-source PLCO cohort which is similarly large or even larger (but lacks molecular data). -I highly recommend to run QC tool (e.g., GrandQC) on the dataset and provide tissue detection maps and QC masks (artifacts masked) in the repo along the images to make the dataset fully usable out-of-box. Moreover, I would like to emphasise high importance of this dataset for the whole AI/pathology community and thank the authors for releasing it.
