# Supplementary material for: SurGen: 1020 H&E-stained whole-slide images with survival and genetic markers
Source: Gigascience. 2025 Oct 8;14:giaf086. doi: 10.1093/gigascience/giaf086 (PMC12569769; doi:10.1093/gigascience/giaf086)
Supplement: giaf086_Reviewer_2_Report_Original_Submission — Dr. Oliver Lester Saldanha -- 10/2/2025 [file giaf086_reviewer_2_report_original_submission.pdf]

Revision-round	0
File-type	reviewer-report
Role	reviewer 2
Name	Dr. Oliver Lester Saldanha
Date-type	reviewer-report-received
Date	10 Feb 2025

Reviewer Report

Title: SurGen: 1020 H&E-stained Whole Slide Images With Survival and Genetic Markers

Version: Original Submission Date: 2/10/2025

Reviewer name: Dr. Oliver Lester Saldanha

Reviewer Comments to Author:

1.Training on External Datasets:To further validate the dataset's robustness, please consider training the models using publicly available datasets such as TCGA and CPTAC for colorectal cancer (CRC) and comparing their performance with models trained on SurGen. This will help highlight the generalizability and advantages of SurGen over existing datasets.

2.External Validation or Core Validation Approach:While the dataset is comprehensive, external validation using an independent cohort would strengthen its impact. If external validation is not feasible, a core validation approach within SurGen (e.g., cross-institutional validation or region-wise stratification) would also be a valuable alternative.

3.Enhanced Comparison with Existing Datasets:The manuscript already provides a comparative table, but an additional quantitative performance comparison of SurGen with other datasets (e.g., TCGA, CPTAC) in terms of model performance would be beneficial.

4.Supplementary Code Placement:Some code is currently included in the main text. Moving it to the supplementary materials would improve the flow of the manuscript while ensuring accessibility for interested readers.
