# Supplementary material for: SurGen: 1020 H&E-stained whole-slide images with survival and genetic markers
Source: Gigascience. 2025 Oct 8;14:giaf086. doi: 10.1093/gigascience/giaf086 (PMC12569769; doi:10.1093/gigascience/giaf086)
Supplement: giaf086_Reviewer_2_Report_Revision_1 — Dr. Oliver Lester Saldanha -- 6/4/2025 [file giaf086_reviewer_2_report_revision_1.pdf]

Revision-round	1
File-type	reviewer-report
Role	reviewer 2
Name	Dr. Oliver Lester Saldanha
Date-type	reviewer-report-received
Date	4th June 2025

Reviewer Report

Title: SurGen: 1020 H&E-stained Whole Slide Images with Survival and Genetic Markers

Version: Revision 1 Date: 6/4/2025

Reviewer name: Dr. Oliver Lester Saldanha

Reviewer Comments to Author:

No Comments
